# The Chemistry of CO_2_ Conversion: A Review

**DOI:** 10.1021/acs.chemrev.5c00361

**Published:** 2026-04-21

**Authors:** R. Gary Grim, Alex Badgett, Wade A. Braunecker, Michael T. Guarnieri, Susan E. Habas, Christopher Hahn, Kenneth Neyerlin, Aditya Prajapati, Daniel A. Ruddy, Roxanne Z. Walker

**Affiliations:** 1 53405 National Laboratory of the Rockies, 15013 Denver W Pkwy, Golden, Colorado 80401, United States; 2 4578 Lawrence Livermore National Laboratory, 7000 East Ave, Livermore, California 94550, United States

## Abstract

For much of the past century, carbon dioxide (CO_2_) has received little attention scientifically outside of its role as a byproduct in the industrialization of the global economy. This trend has recently been upended where, due to mounting environmental concerns, CO_2_ has been brought squarely into the public consciousness. This surge in activity has contributed to a once unimaginable idea now pervading the scientific community: could CO_2_, a highly stable byproduct of hydrocarbon combustion, be recycled and converted back into useful chemicals and fuels? Owing to its ubiquitous nature and availability at truly massive quantities, it is thought that CO_2_-based products could offer a meaningful pathway toward lowering the environmental impact of many of the top industrial products while also enhancing supply chain diversification and resilience. In this manuscript we provide a holistic review of the pathways for CO_2_ conversion, the underlying chemistry and challenges involved in the transformation to products, and considerations for commercialization.

## Graphical abstract

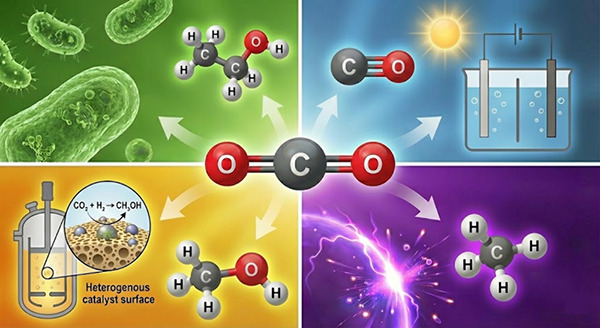

## 1 Introduction

Over the past decade, estimates suggest nearly 360 billion tonnes of carbon dioxide (CO_2_) were emitted as a result of human activities, pushing atmospheric concentrations in excess of 419 ppm, a level not seen on Earth in some three million years.^1,2^ As concerns over the impact of rising CO_2_ emissions continue to grow, policymakers and scientists around the globe are being called into action to pursue strategies for mitigation.^3^ Once unimaginable, one of the prevailing ideas now receiving widespread international attention is the concept using the CO_2_ molecule itself as a carbonaceous resource for producing the chemicals and fuels consumed in everyday life.^4–8^ Specifically, in processes colloquially referred to as “Power-to-X”, “e-fuels”, or “e-products”, by pairing electrons or other electricity-sourced energy carriers (e.g., H_2_) with novel conversion pathways across electrochemistry, photochemistry, plasma science, biology, and thermochemistry, recent studies have shown proof of concept for the transformation of CO_2_ into a variety of species spanning a range of chemical functionalities.^4,9–11^ Comprising predominantly lower molecular weight species such as carbon monoxide (CO), methanol (MeOH), methane (CH_4_), ethanol (EtOH), and ethylene (C_2_H_4_), it is proposed that many of the top industrial products could be synthesized from these platform molecules as e-products through technically mature routes such as Fischer–Tropsch (FT), methanol-to-olefins (MTO), and other established chemical pathways currently utilized across industry.^12^

Within the past several years support for CO_2_ conversion and e-products has moved from a fringe concept relegated to academic laboratories to now entering the mainstream where some companies are actively pursuing and/or operating pilot and commercial scale operations based around CO_2_ conversion. Governments worldwide have also begun to show support for CO_2_ conversion, highlighted by recent policies from the United States (e.g., SAF Grand Challenge, 45Z credits)^13^ and in the European Union. As part of the recently announced ReFuelEU aviation program, mandates are now in place that require fuel suppliers to gradually increase the amount of synthetic fuel (i.e., CO_2_-based e-fuels) across EU airports from 1.2% of total fuel supplied in 2030 to 35% in 2050.^14^

The conversion of CO_2_ is not without its challenges, however.^4^ Shown in Table 1, the CO_2_ molecule represents a significant departure from the conventional feedstocks on which current economies are based. Being itself a product of the hydrocarbon combustion reaction, CO_2_ has no intrinsic energy content (i.e., heating value), exists in a highly stable and fully oxidized state, and is comprised predominately of oxygen (∼73% by mass). By comparison, conventional feedstocks like crude oil and natural gas are comprised of less than 1.5% oxygen by mass and carry a significantly higher inherent energy content in the range of 42 – 47 MJ/kg, providing a favorable starting point for the synthesis of fuels and chemicals. Indeed, prior to 15–20 years ago, the idea of CO_2_ conversion to hydrocarbon products would be considered by most as nonsensical. In addition to the uphill energetic challenges of rebuilding a CO_2_ molecule, when powered by energy sources or reactants that are predominately fossil in origin, the conversion itself will, in most instances, be more energy intensive and costly and therefore generate even more CO_2_ emissions than simply making the products directly from conventional resources.^15–18^

**Table 1:** Heating Values and Oxygen Content Across CO_2_ and Common Feedstocks

	Lower Heating Value (MJ/kg)^a^	Oxygen Content (w/w%)
Carbon Dioxide	0.0	72.7
Natural Gas	47.1	0.01
Crude Oil	42.7	<1.5
Bituminous Coal	26.1	6–8
Corn Stover	16.4	41.5^b^
Sugar cane Bagasse	15.1	48.8^c^
Woody Biomass	19.6	41.2^d^
Hydrogen Gas	120.2	0.0

a Ref 19.

b Ref 20.

c Ref 21.

d Ref 22.

However, as the rate of renewable energy deployment has increased dramatically in recent years and the cost has, in some areas of the world, reached a point lower than conventional fossil energy,^23^ that paradigm is now beginning to shift. Some forward-looking analyses now find that despite being an inherently energy intensive process, if low carbon intensity electricity is made widely abundant at low enough cost, CO_2_ conversion pathways could potentially produce products at a price point at, or even below, that of conventional fossil-based products with, in most cases, a significantly lower carbon emissions profile.^24–26^ This realization combined with the explosive growth of renewable energy has led to a flurry of activity in CO_2_ conversion research where over the past 15 years the number of publications referencing “CO_2_ conversion” has increased exponentially from only ∼ 300 in 2010 to now over 4,700 per year in 2024 (Figure 1).

**Figure 1:**
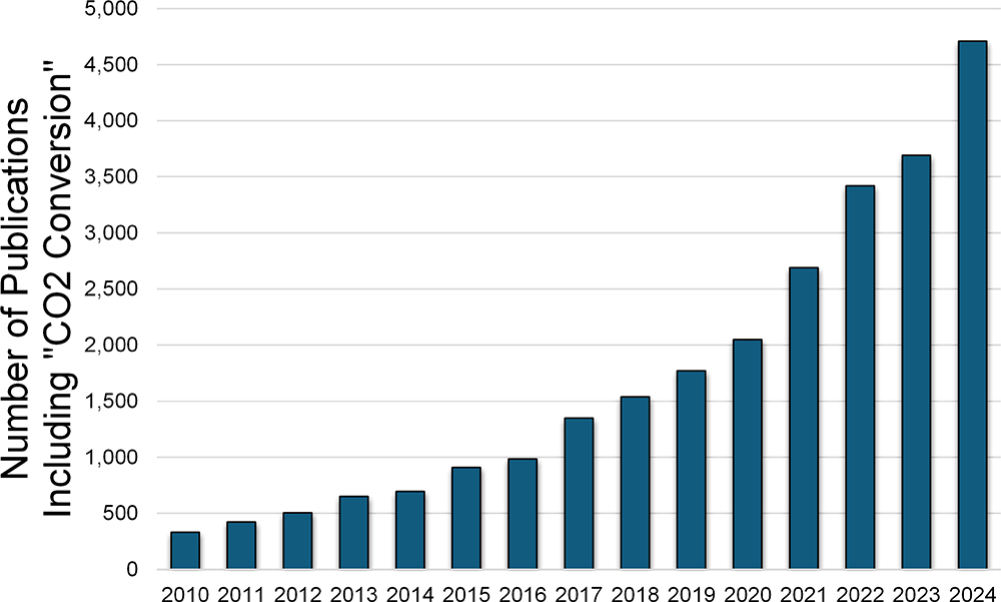
Number of publications by year mentioning the term “CO_2_ Conversion” as indexed by Google Scholar.

This emerging intersection of low-cost and low-carbon intensity electricity with CO_2_ conversion has significant potential implications for global carbon circularity and the future of sustainable products. Owing to its abundance in the carbon cycle, CO_2_ represents a sustainable carbon resource available in truly massive quantities, sufficient to easily meet the entire global demand for carbon-based products many times over.^4^ By reusing this abundant “waste” carbonaceous resource, it creates the opportunity for a closed-loop circular carbon economy whereby products with a near carbon-neutral footprint are possible. Another key differentiator of CO_2_ is that it is not geographically constrained to any one region but rather is ubiquitous across the globe. This widespread availability combined with the ability to potentially source directly from the atmosphere via direct air capture (DAC) without the need for extensive transportation infrastructure creates unique opportunities in siting and colocation that may offer strong economic incentives. This potential for a self-contained feedstock-to-product design increases the inherent resiliency of supply chains which in the future could serve to ease or avoid entirely disruptions like those felt in the early 2020s (e.g., global pandemic, geopolitical conflicts).^27,28^

Ultimately, unlocking the future potential of CO_2_ conversion and e-products will rely on solving two fundamental challenges: (1) the ability to source low-carbon intensity electricity at a sufficiently low price point and high capacity factor to provide compelling economic opportunities, and (2) the ability to efficiently and selectively channel that energy toward CO_2_ conversion via carbon-carbon and carbon-hydrogen bond formation. While renewable energy deployment is accelerating,^29^ conversion efficiencies remain a significant bottleneck.^30,31^ Recent studies estimate the current energy return on energy invested (EROEI) for some CO_2_-derived products at 0.33 or lower, meaning the net energy demand for synthesis, CO_2_ capture, and energy infrastructure is triple that of the energy ultimately delivered by the product.^32^ Compared to conventional products (e.g., system-level EROEI ∼ 11 – 14 for SMR-sourced methanol), these low values represent a significant thermodynamic gap. Given that an EROEI of > 3 is generally cited as the minimum threshold to be “useful” for society,^32^ low energy efficiency CO_2_ conversion puts e-products at risk of being “energy sinks” at a time when energy demand worldwide continues to accelerate meaningfully.^8^ While system-level EROEI of e-products is inextricably linked to the primary energy source—where high EROEI sources like nuclear power (>50) offer a robust baseline—this reality underscores a critical need in the field of CO_2_ conversion is an improved fundamental understanding of the chemical mechanisms and challenges in efficiently utilizing the reducing potential of electrons and other energy carriers to boost system-level efficiency.

In this review we discuss the recent advancements in the fundamental understanding in CO_2_ conversion chemistry across seven prominent conversion pathways and, importantly, assess how this knowledge contributes to the rational design of processes, improvements in technical performance, and ultimately the viability of CO_2_ conversion. To focus the scope of this review, we consider only reports published in the last six years (2020 – 2025) and cover only the processes associated with the conversion of CO_2_ to value-added products or intermediates. In other words, we highlight only the chemical pathways that directly convert CO_2_ and do not include discussion on subsequent downstream upgrading steps (e.g., syngas chemistry, alcohol upgrading, etc.) which have been reviewed elsewhere.^33,34^ Herein we structure the review based on the way in which energy is consumed. In the first part of the review, electron-mediated conversion chemistries such as electrolysis, photocatalysis, nonthermal plasma, and microbial electrochemical processes are reviewed. In the following section, we consider H_2_-mediated chemistries including CO_2_ hydrogenation via thermocatalysis and biological conversion of CO_2_ via fermentation. In the final sections of the manuscript, we review emerging trends across CO_2_ conversion chemistry, compare pathways using cross-cutting technical metrics, discuss the logistics and barriers to scaling up CO_2_ conversion, and highlight future R&D priorities for CO_2_ conversion technologies.

## 2 Electron-Mediated CO_2_ Conversion

### 2.1 Low-Temperature Electrolysis

Low-temperature electrolysis (LTE) for CO_2_ is an electrochemical process that converts CO_2_ into value-added chemical feedstocks using electrical energy.^35^ This pathway typically operates between 25–80 °C and in a pressure regime of 1–20 bar.^35^ These operating parameters distinguish it substantially from high-temperature solid-oxide electrolysis (SOEC), which requires temperatures in excess of 800 °C to utilize solid-state ion conductors.^35,36^

LTE presents a strategic pathway toward industrial decarbonization. It provides a direct electrochemical route to convert CO_2_ into value-added fuels and chemicals, offering an alternative pathway to replace the traditional energy and emissions-intense thermocatalytic, fossil-fuel-based processes. Furthermore, LTE systems can function as a flexible electrical load, capable of utilizing surplus renewable electricity from intermittent sources such as solar and wind. This capability addresses the challenge of grid instability from renewable curtailment and enables greater penetration of clean energy.^35,37^ The primary strengths of this pathway lie in its direct conversion of electrical energy into storable chemical bonds, its operation under mild conditions that are compatible with existing industrial infrastructure, its modularity that provides practical advantages for distribution site selection and operational flexibility, and its feedstock versatility in utilizing waste CO_2_ as a carbon source. The overall process of LTE is driven by a full electrochemical cell, which comprises two distinct half-reactions. At the cathode, the negative electrode, CO_2_ is reduced in the CO_2_ reduction reaction (CO_2_RR) to form a variety of chemical products such as CO, Formic acid, ethylene, etc. At the anode, the positive electrode, a corresponding oxidation reaction (typically, water oxidation) must occur to complete the electrical circuit.^38^ Detailed half reactions on cathode and anode are provided in the Supporting Information.

Currently, the high-rate LTE research for CO_2_RR is centered on the membrane electrode assembly (MEA), a zero-gap electrolyzer architecture adapted from fuel cell technology.^39^ This design minimizes ohmic resistance, enabling the high current densities required for commercial viability. While a detailed breakdown of device components is available in the Supporting Information, the main chemistry of a CO_2_ electrolyzer is centered at the cathode.^40^ Cathode is fabricated as a porous gas diffusion electrode (GDE), a complex structure engineered to create a stable three-phase interface for gaseous CO_2_ reactant, a liquid electrolyte or ionomer phase for ion transport, and the solid heterogeneous catalyst surface where the electrochemical reaction occurs. At the anode, oxygen evolution reaction (OER) occurs to complete the electrical circuit while an ion-conducting polymer membrane is positioned between the electrodes to transport ions and prevent reactant and product crossover.

To evaluate and compare the chemical performance of these systems, a set of key metrics is described here with further details on their calculations in Supporting Information. (1) Faradaic Efficiency (FE,%): This quantifies the selectivity of the process, defined as the fraction of total electrons that are used to form a specific, desired product. (2) Partial Current Density (j, mA/cm^2^): This represents the productivity or rate of product formation per geometric electrode area. (3) Full-Cell Energy Efficiency (EE,%): The overall energy conversion efficiency, representing the ratio of the Gibbs free energy stored in the chemical product to the total electrical energy consumed by the cell. (4) Single-Pass Carbon Conversion (SPC): The fraction of CO_2_ reactant converted into products in a single pass through the reactor.

The field CO_2_RR is chemically broad, spanning multiple classes of catalysts.^38,41,42^ We acknowledge that seminal advances in homogeneous catalysis using molecular catalysts (e.g., transition-metal complexes) dissolved in solution, and immobilized catalysis, have played a formative role in shaping the mechanistic understanding of CO_2_ activation and product selectivity. Ligand-controlled electronic structure, hydride transfer chemistry, and site isolation, have directly influenced heterogeneous motifs including Co-complex–derived methanol catalysts, atomically dispersed M-N-C sites, and other hybrid architectures.^42^ Consequently, heterogeneous catalysis, that utilizes solid-state materials such as metal nanoparticles (e.g., Cu, Ag, Sn), oxide-derived materials, and carbon-based catalysts,^38,39^ has shown a clearer translation of the chemistry advances toward scalable, high-current-density electrolyzer systems has been achieved using heterogeneous materials. Therefore, this section of the review will bound its discussion and focus exclusively on the advances in the chemistry of heterogeneous catalysis for CO_2_RR. We emphasize how reaction mechanisms, interfacial chemistry, and catalyst-ionomer interactions have guided the design of the modern design-principles of low temperature electrolyzers. In that framework, we have organized this discussion chronologically from foundational mechanistic insights prior to 2020, to the post-2020 advances in electrolyzer design and microenvironment control and concluded by identifying key opportunities that will define the field’s trajectory.

Prior to 2020, foundational discoveries established the core principles of reaction mechanisms, the critical influence of catalyst restructuring and the local chemical environment, and the emergence of the scale-up bottleneck inclusive of an energy penalty related to carbonate crossover. This period saw the field grow from fundamental studies in aqueous H-cells to the first high-rate gas-phase electrolyzers. The pioneering work of Hori et al. established that product selectivity in CO_2_RR is, to a first approximation, determined by the identity of the metal electrocatalyst.^43^ The selectivity of these metal electrocatalysts for CO_2_RR, later rationalized by computational density functional theory (DFT) studies,^38,44^ identified distinct product classes. In the case of C_1_ products (CO and Formate), the initial branching of CO_2_ reduction is dictated by the binding of the first intermediate. Metals such as Au, Ag, and Zn weakly bind carbon. They are believed to stabilize CO_2_ adsorption and the *COOH intermediate, which, after a second electron–proton transfer, desorbs as CO.^40^ In contrast, oxophilic metals like Sn, Bi, and In preferentially stabilize the oxygen-bound *OCHO intermediate,^45^ which is subsequently reduced to formate (HCOO^–^).^38,40^ For C_2+_ products, Cu is unique among elemental metals in its ability to catalyze the reduction of CO_2_ to valuable multicarbon products, such as ethylene (C_2_H_4_) and ethanol (C_2_H_5_OH).^46^ This multielectron (12e^–^ for ethylene), multiproton pathway is vastly more kinetically complex than the 2e^–^ pathways to C_1_ products.^44,47,48^

The activation of CO_2_ and subsequent reduction steps were often described within the framework of proton-coupled electron transfer (PCET), where an electron transfer from the electrode and a proton transfer from solution occur in a concerted fashion.^49^ While the PCET model is powerful, particularly for describing the initial CO_2_-to-*COOH step, it is now understood to be an oversimplification for the entire complex reaction network.^38,40^ A more nuanced understanding, developed through both computational and experimental work,^50^ posits that many key transformations may proceed via decoupled sequential pathways (e.g., an electron transfer followed by a separate proton transfer step) or as pure chemical steps (e.g., protonation of a surface intermediate without a simultaneous electron transfer).^40^ The dominant mechanism (concerted, sequential, or chemical) is highly dependent on the applied potential, local pH, and specific catalytic environment.^51^ A representative reaction network based on the aggregate view that the field proposes is shown in Figure 2. An important mechanistic challenge that emerged is the bifurcation between the ethylene and ethanol pathways. Extensive computational and *operando* studies have proposed competing routes involving *HCCO, HCCHO, or H_2_CCHO intermediates,^52–54^ yet no conclusion has been reached on the precise branching point. This uncertainty mirrors the broader ambiguity surrounding C_3+_ formation mechanisms, where multiple multielectron, multiproton pathways remain kinetically and spectroscopically indistinguishable. Additionally, the challenge of sequential and multiple C-C coupling steps lead to low selectivity of C_3+_ hydrocarbons.^38,39^ A more practical strategy, established in this period, is a two-step hybrid approach: (1) LTE is used to produce a simple, energy-dense building block like CO (or syngas) at high selectivity, and (2) this intermediate gas is then fed into established thermocatalytic processes like Fischer–Tropsch synthesis for upgrading.^38,55^ CO reduction in tandem with CO_2_RR emerged as another complementary route that enables higher selectivity for C_3+_ liquid products and oxygenates.^56^ These parallel two-step strategies broaden the design space for producing more complex hydrocarbons from CO_2_.

**Figure 2:**
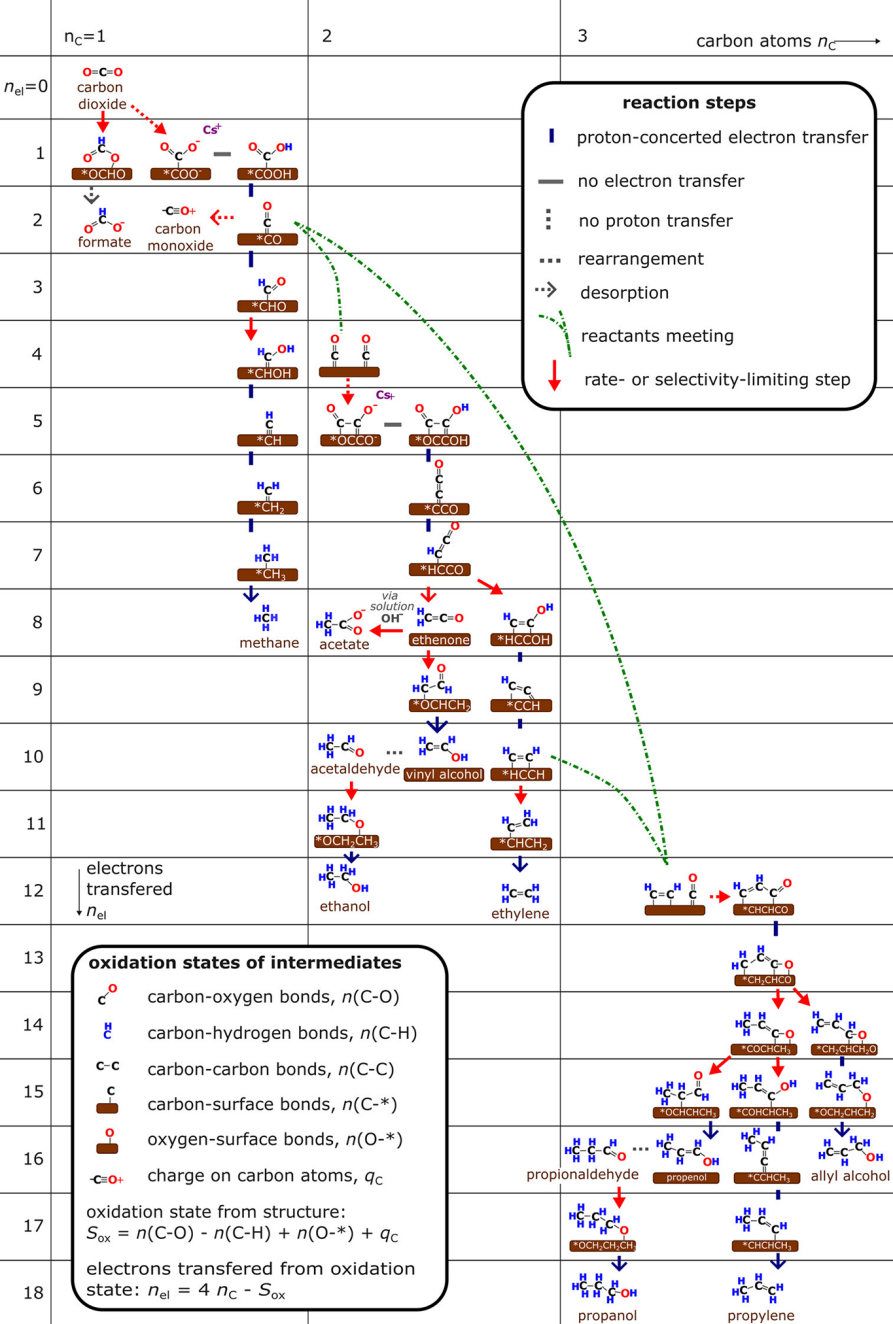
Proposed reaction network for the electrochemical reduction of CO_2_ to various C_1_, C_2_, and C_3+_ products. The formation of the *CO intermediate is a key branching point for many reaction pathways. Adapted from ref 40. Copyright 2025 American Chemical Society. Although the figure reflects the consolidated, post-2020 understanding of CO_2_RR pathways, many of the mechanistic elements that define this network originated from pre-2020 work as shown in ref 38.

Research prior to 2020 also established that beyond just catalyst metal identity, CO_2_RR is highly sensitive to more subtle factors such as the catalyst’s physical structure and its local chemical environment. Surface-science studies demonstrated that CO_2_RR on Cu is structure-sensitive.^57,58^ The Cu(100) facet was shown to be more selective for ethylene, whereas Cu(111) preferentially produced methane. This was attributed to the different adsorption energies of key intermediates on these distinct atomic arrangements.^35^ A breakthrough was the discovery of oxide-derived (OD) catalysts (e.g., Cu derived from Cu_2_O reduction).^59–61^ These high-surface-area, structured materials exhibited dramatically enhanced C_2+_ selectivity compared to their nanoparticle or foil counterparts.^39^ This enhancement was hypothesized to be due to a combination of high porosity, stable grain boundaries, and a high density of active defect sites.^57^ Similar structural engineering principle were observed in C_1_ catalysts such as Ag^62^ and Sn^63^ for enhanced activity and selectivity.

For the local chemical environment, two factors were identified as major modulators of reactivity. First, the local pH at the catalyst surface, which can be significantly higher than the bulk electrolyte during reaction, was shown to suppress the competing HER.^64–66^ Second, a strong promotional effect was identified for large alkali metal cations (e.g., Cs^+^, K^+^).^67,68^ The consensus mechanism proposes that these large, weakly hydrated cations accumulate at the outer Helmholtz plane, creating an intense local electric field.^69^ This field is believed to stabilize the negatively charged *CO_2_^–^ transition state, lowering the kinetic barrier for CO_2_ activation, while also structuring interfacial water to suppress HER.

By 2020, the field’s momentum for high FE and current density led to the widespread adoption of the Anion Exchange Membrane (AEM) electrolyzer.^35^ The chemical rationale was to use AEMs to selectively transport anions (OH^–^), creating a highly alkaline local environment at the cathode, thereby suppressing HER and promoting CO_2_RR. However, the same alkaline environment drives the rapid reaction of CO_2_ with OH^–^ to form carbonate: *CO*_2_ + 2*OH* ^–^ → *CO*_3_^2–^ + *H*_2_ *O*. This carbonate becomes the primary charge-carrying species and migrates across the AEM to the anode, where it is neutralized by protons from OER and rereleased as CO_2_ gas: *CO*_3_^2–^ + 2*H* ^+^ → *CO*_2_ + *H*_2_ *O*. This carbonate crossover problem establishes a stoichiometric limit on carbon conversion efficiency. For the 2-electron reduction to CO (which produces 2*OH* ^–^), a 2-charge *CO*_3_^2–^ must cross the membrane to balance the charge. Therefore, for every one mole of CO produced, one mole of CO_2_ is wastefully transported and lost to the anode. This fixes a theoretical maximum SPC of 50% for CO (and a similarly derived 25% for ethylene).^70^ It is important to note, however, that this theoretical limit is not universal. Under operating regimes where OH^–^ is a dominant charge carrier, the effective maximum SPC may exceed these constrained values. These observations remain anecdotal and system-dependent,^71,72^ but they indicate that charge-carrier identity is a function of local chemistry rather than a fixed boundary condition. In any case, the CO_2_ lost due to carbonate crossover to the anode must be separated from the oxygen stream (an energy-intensive process) and recycled, imposing a massive energy penalty that could undermine the entire economic viability of the technology. This realization was a critical inflection point, demonstrating that simply optimizing catalysts within the dominant AEM architecture was an insufficient and perhaps nonviable path to commercialization. It motivated the field to consider alternative electrolyzer designs where differences in ion transport and interfacial chemistry could open new pathways for improving performance in the coming years.

By 2020, increasing technological maturity and growing private-sector interest pushed the field toward more integrated MEA and device-level studies. This transition revealed a broader set of transport and interfacial challenges that emerge only under industrially relevant conditions. Research efforts rapidly diversified, moving beyond simple catalyst optimization in AEMs to two new, interconnected frontiers: 1) the rational design of novel electrolyzer architectures explicitly to mitigate or eliminate carbonate crossover,^73–75^ and 2) a more fundamental investigation into the catalytic microenvironment^76,77^ to gain new levers of control over reaction pathways. The primary response to the stoichiometric limitations of AEMs led to the development and optimization of systems based on bipolar and proton exchange membranes.

Bipolar Membranes (BPMs) are composite structures containing both an anion exchange layer (AEL) and a cation exchange layer (CEL). This structure acts as a physical barrier to anion transport from cathode to anode, chemically isolating the two compartments. In the reverse-bias configuration (with the CEL facing the cathode), the cathode becomes acidic, which reduces carbonate formation but introduces a substantial challenge: the high proton concentration favors HER, resulting in very low FE’s for CO_2_RR.^78,79^ Conversely, the forward-bias configuration (with the AEL facing the cathode) maintains a kinetically favorable alkaline environment at the cathode. Here, H^+^ from the anode and OH^–^ from the cathode migrate into the membrane junction where they recombine to form water, enabling a pure-water anolyte feed and avoiding salt precipitation and carbonate crossover. However, this architecture introduces a mechanical-integrity challenge of accumulation of CO_2_ and water at the internal membrane junction can lead to delamination or catastrophic cell failure,^73,79,80^ though recent approaches involving perforated layers incorporated into BPMs have the potential to mitigate these failure modes.^81^ BPM systems mitigate carbonate crossover by introducing an internal water-dissociation junction, but this approach can impose additional energy and transport penalties. As a result, parallel efforts have been explored in proton exchange membrane (PEM)-based architecture to eliminate the formation of carbonate.

Running CO_2_ electrolysis using a PEM eliminates carbonate formation but historically was considered impractical because the high H^+^ concentration at the cathode favors HER.^82^ Post-2020 work has shown that this limitation is not absolute: by engineering the local microenvironment, specifically introducing high concentrations of alkali metal cations into the acidic catalyst layer, the cation-effect can stabilize CO_2_RR intermediates and suppress HER,^82,83^ enabling meaningful CO_2_RR activity even under acidic conditions. Although still emerging, this approach offers a promising path toward carbonate-free CO_2_ electrolysis in proton-conducting architectures.

Since 2020, advances in device architecture have been accompanied by a more fundamental understanding of the catalytic interface where water activity, ionomer structure, and organic additives directly influence reaction pathways.^76,84^ The field has begun to appreciate water not just as a passive solvent, but as a tunable reactant and mediator. A notable 2023 study demonstrated that tuning the activity of water, by adding hygroscopic solutes to the electrolyte, is a powerful and previously unrecognized lever for controlling selectivity.^85^ The key finding was that lowering the water activity suppressed the formation of H_2_ and CO, but promoted the formation of desirable C_2+_ multicarbon products.^85^ Another study shows the deviations in water and solute activities within concentrated electrolytes alters reaction energetics and mass-transport profiles, directly influencing CO_2_RR product distributions and validating water-activity tuning as a mechanistic control parameter.^86^ This provides a novel strategy to modulate microenvironment to influence reaction selectivity.

A parallel strategy for engineering the microenvironment involves the use of organic additives or advanced polymer electrolytes.^87^ Recent work has shown that tailored organic films can assemble on Cu surfaces and act as selective proton-transport barriers, attenuating the flux of H^+^ to the catalytic interface and thereby suppressing HER even under acidic conditions.^88^ Building on this, a 2024 study demonstrated that engineered organic thin films can stabilize otherwise transient Cu^+^ species under CO_2_RR conditions and reshape the interfacial ionic environment, leading to enhanced C_2+_ selectivity.^89^ Collectively, these additive-based approaches provide a chemical route to modulate interfacial transport and reactivity, enabling > 70% FE to multicarbon products in environments that would otherwise be dominated by HER. Historically, the ionomer within the catalyst layer was viewed primarily as a structural binder and ionic conductor. However, a growing body of post-2020 literature has demonstrated that the ionomer has a direct and active influence on CO_2_RR kinetics and selectivity.^90^ Recent studies have shown that ionomer chemistry, including fixed charge, hydrophobicity, and molecular architecture, can substantially alter local solvation, interfacial pH, and the adsorption thermodynamics of key intermediates.^90–92^ *Operando* spectroscopic measurements have been instrumental in establishing these effects. Using *operando* Raman, FTIR, and synchrotron-based techniques, researchers have shown that anionic fluorinated ionomers such as Nafion and cationic AEMs such as Sustainion produce measurably different *CO adsorption behaviors at Cu and Ag surfaces, leading to divergent selectivity between CO and C_2+_ products.^93^ These measurements provide direct evidence that the ionomer controls local intermediate coverage rather than merely facilitating ion transport. In a notable recent study, the combination of operando Raman spectroscopy and differential electrochemical mass spectrometry yielded the first spectroscopic detection of a *CCO-type intermediate at the electrode/polyelectrolyte interface, confirming the role of the ionomer in stabilizing multicarbon pathways under realistic operating conditions.^94^ The convergence of these insights such as ionomer-induced local pH gradients, cation-ionomer interactions, solvation structure, and interfacial conformational effects, marks a substantive evolution in the mechanistic understanding of CO_2_RR. Rather than treating the electrode as immersed in a spatially uniform bulk electrolyte, current models recognize the reaction as occurring within a nanometer-scale, dynamic interphase governed by the specific arrangement of polymer chains, ions, and water molecules. This interphase-centric view has become foundational for the rational design of next-generation catalyst layers, ionomers, and MEAs optimized for high-rate, selective CO_2_RR. Figure 3 further shows the evidence that ionomer is not merely a passive binder but an active component that directly modulates the local environment.

**Figure 3:**
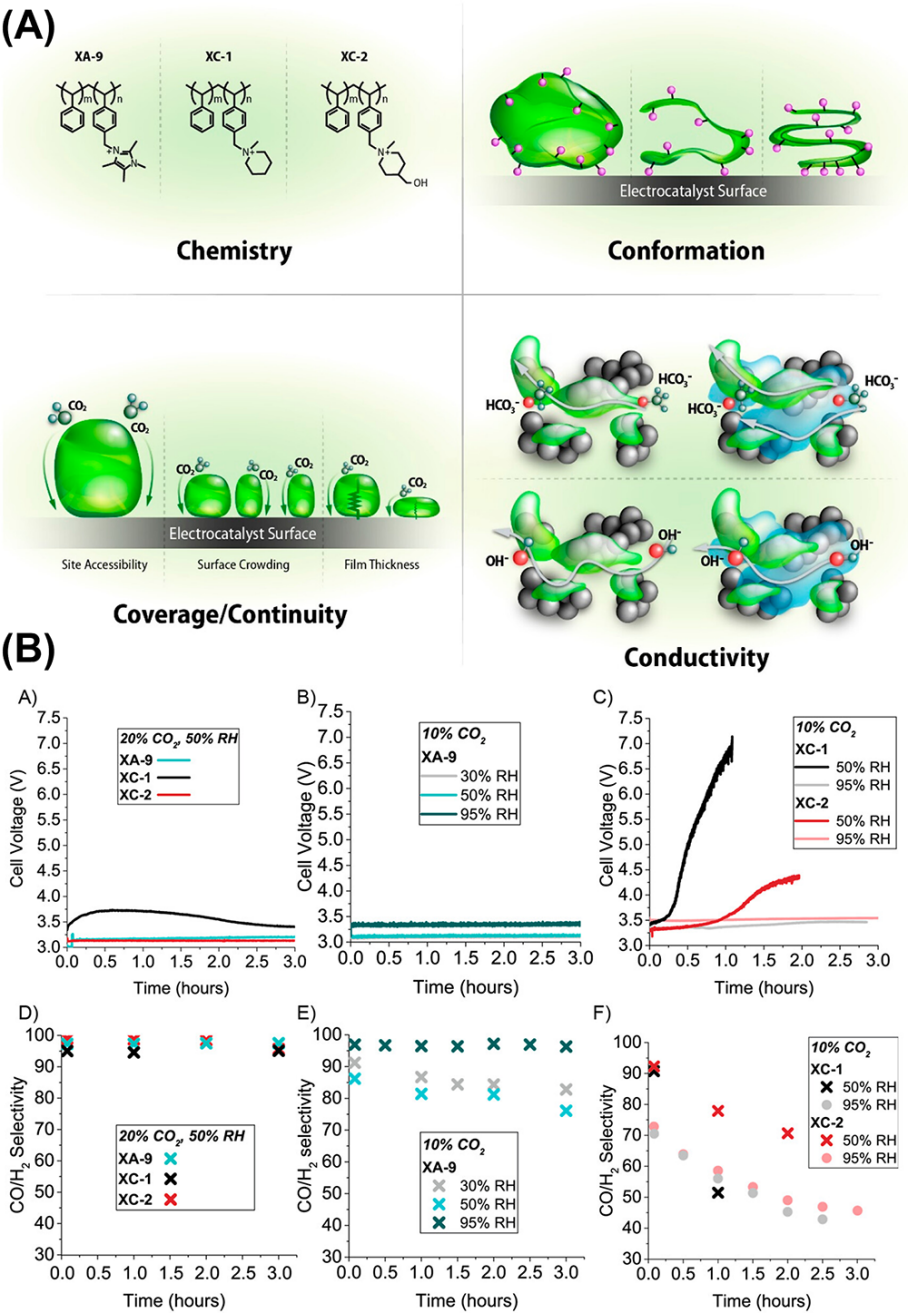
(A) A schematic illustrating the various impacts that ionomer properties such as chemistry, conformation, and coverage can have on the electrochemical properties of the electrode and the local reaction environment. (B) Effect of different ionomers on CO_2_ electrolyzer performance. Adapted from ref 92. Copyright 2024 American Chemical Society.

The deeper understanding of interfacial chemistry has been crucial for reframing durability not as a late-stage engineering problem, but as a set of fundamental chemical challenges rooted in transport phenomena and materials stability that need to be addressed in early stage research.^95^ Traditionally treated as an engineering problem, degradation is increasingly recognized as a chemically driven phenomenon governed by the stability and reactivity of interfaces under nonequilibrium conditions. The coupling of electrochemical potentials, local pH gradients, and reactive intermediates gives rise to self-evolving catalyst structures and complex transport failures. A major advance, driven by *operando* characterization studies,^96,97^ highlights that catalysts are not static entities. Under the harsh, reducing potentials of CO_2_RR, metal surfaces are dynamic. A key finding on Cu is that the reaction may not even occur on the as-synthesized perfect (100) or (111) facets. Instead, the strong adsorption of the *CO intermediate provides a thermodynamic driving force for the catalyst to reconstruct, forming a high density of active steps and kinks. The true active site is now believed to be a dynamic structure created during the reaction, often at sites adjacent to these defects.^98^ This insight helps explain why disordered, high-defect materials like OD-Cu show such high C2+ selectivity.

A significant fraction of catastrophic cell failures in low-temperature CO_2_ electrolyzers can be traced to chemical transport imbalances rather than mechanical failure modes. These degradation phenomena originate from the coupled migration of ions, water, and reactive species across porous and polymeric interfaces, which collectively determine the stability of the local reaction environment. Among the most pervasive chemical degradation pathways is salt precipitation, a consequence of ion crossover and carbonate accumulation. In AEM systems, cations such as K^+^ from the anolyte migrate across the membrane and encounter the high local concentrations of carbonate and bicarbonate species generated at the cathode. KHCO_3_ nucleates and grows within the GDL pores which obstructs gas pathways, impedes CO_2_ access to active sites, and can lead to abrupt, irreversible cell failure.^78^ The local water content (λ, or water molecules per ionic group) in the ionomer is a critical descriptor for designing an efficient catalyst.^91^ Maintaining the appropriate λ requires balancing electro-osmotic drag, which pulls water from anode to cathode, with back-diffusion, which drives water in the opposite direction resulting in either flooding or dehydration of the GDE. Flooding (high λ) occurs when excess hydration leads to liquid water accumulation within GDL pores, blocking gaseous CO_2_ transport and suppressing catalytic activity. Dehydration (low λ) takes place when insufficient hydration dries the membrane and ionomer, reducing ionic conductivity and inducing mechanical cracking due to differential swelling stresses. This balance of water uptake is not only a physical property but also a chemical descriptor of selectivity, as local water activity directly modulates CO adsorption strength and C-C coupling probability. A representative data set in Figure 4 illustrates this relationship, showing how ionomer hydration levels influence ethylene FE at 200 mA cm^–2^. Advances in understanding these coupled transport phenomena have guided the rational design of hydrophobic GDL architectures and the implementation of controlled humidification and thermal management strategies, enabling sustained operation and improved long-term stability. A comparative summary of the degradation mechanisms, trade-offs, and mitigation strategies across state-of-the-art CO_2_RR architectures is tabulated in the *Supporting Information*.

**Figure 4:**
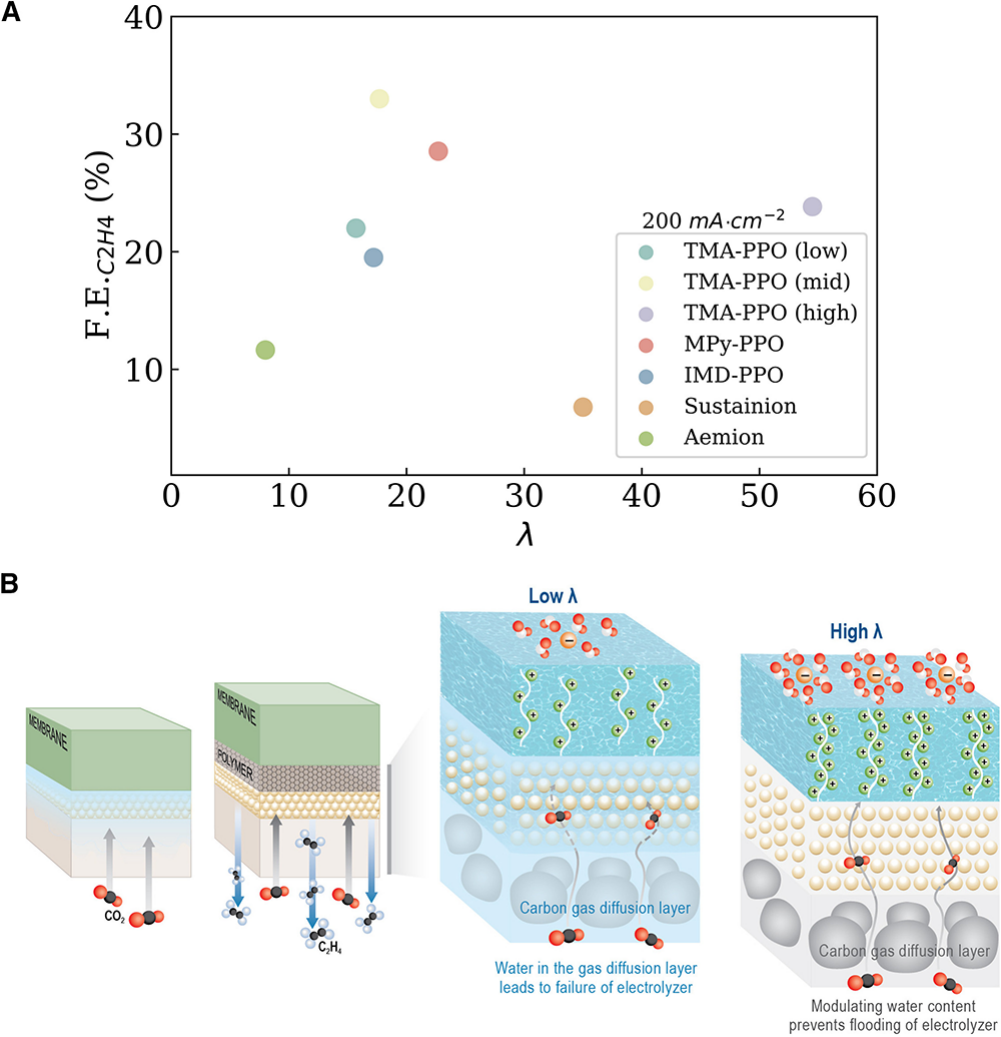
Influence of ionomer λ on selectivity and flooding behavior. (A) Ethylene F.E. at 200 mA/cm^2^ for all ionomers in this study as a function of λ. (B) Schematic illustration of how GDL floods without an ionomer binder and when λ is low. Adapted with permission from ref 91. Copyright 2025 Elsevier.

The fundamental chemical insights gained over the last five years have translated directly into tangible and, in some cases, transformative improvements in electrolyzer performance, durability, and viability. The progress is not the result of a single breakthrough but rather the synergistic effect of advancements in architecture, interfacial control, and materials design. Recent progress illustrates how a molecular-level understanding of electrochemical phenomena can yield system-level gains. The quantitative chemical analysis of the carbonate problem prompted the rapid emergence of BPM and PEM architectures, enabling CO_2_ utilization efficiencies well beyond the ≈ 50% SPC values typically observed in anion-exchange systems, substantially lowering the carbon-recycle load and projected energy intensity of downstream separations. The mechanistic understanding of the cation effect resulted in a practical design strategy for acidic environments. By engineering high local alkali-cation concentrations at the cathode-electrolyte interface, researchers mitigated competitive HER,^83^ and also developed PEM CO_2_ electrolyzers with > 90% FE for formic acid and durability exceeding 5,000 h at > 600 mA cm^–2^ with hydrogen oxidation reaction (HOR) at the anode.^99^ These results open avenues for exploring acidic systems for scale-up development as an industrially viable platform. Finally, recognition that ionomers actively mediate local reaction environments has spurred the development of bifunctional binders that couple ionic conductivity with catalytic modulation. Such materials have enabled CO_2_ coelectrolysis at high current densities exceeding 300 mA cm^–2^ while retaining high selectivity, marking a pivotal advance toward economically scalable electrolyzer stacks.

Despite these achievements, the field now faces a new frontier of chemical stability and kinetic limitations. The durability of membranes and ionomers remains the critical bottleneck for commercialization. Each architecture faces distinct degradation pathways. In AEMs, nucleophilic attack by concentrated OH^–^ leads to backbone cleavage and quaternary-ammonium loss. PEMs suffer oxidative radical attack under high anodic potentials, while BPMs endure extreme electric-field gradients and gas-evolution stress at the cation/anion interface, often resulting in delamination. Within the GDE, the ionomer experiences compressive and shear stresses from gas evolution, swelling from water uptake, transient flooding, and salt crystallization, leading to pore collapse, loss of adhesion to catalyst particles, and degradation of the three-phase boundary. Although substantial progress has been made in identifying highly active catalysts for the CO_2_-to-CO step, their turnover frequencies now approach those of state-of-the-art OER catalysts at comparable overpotentials that suggests that the kinetics of the first two-electron reduction step are no longer the primary bottleneck. In contrast, the formation of multicarbon products remains orders of magnitude slower. As highlighted in Figure 5, TOFs for ethylene remains far below those accessible for CO formation, reflecting the intrinsically more complex, multistep C-C coupling chemistry. This kinetic gap is further amplified by competing pathways and branching between ethylene, ethanol, and higher oxygenates, as well as the challenge in accessing routes to C_3+_ products under practical operating conditions. Slow C-C coupling kinetics, pathway bifurcation, and selectivity limitations lead to low FEs and necessitate higher overpotentials, both of which negatively impact overall energy efficiency. This emphasizes the need for catalysts and electrolytes that co-optimize charge transfer, solvation, and intermediate stabilization under industrial current densities.

**Figure 5:**
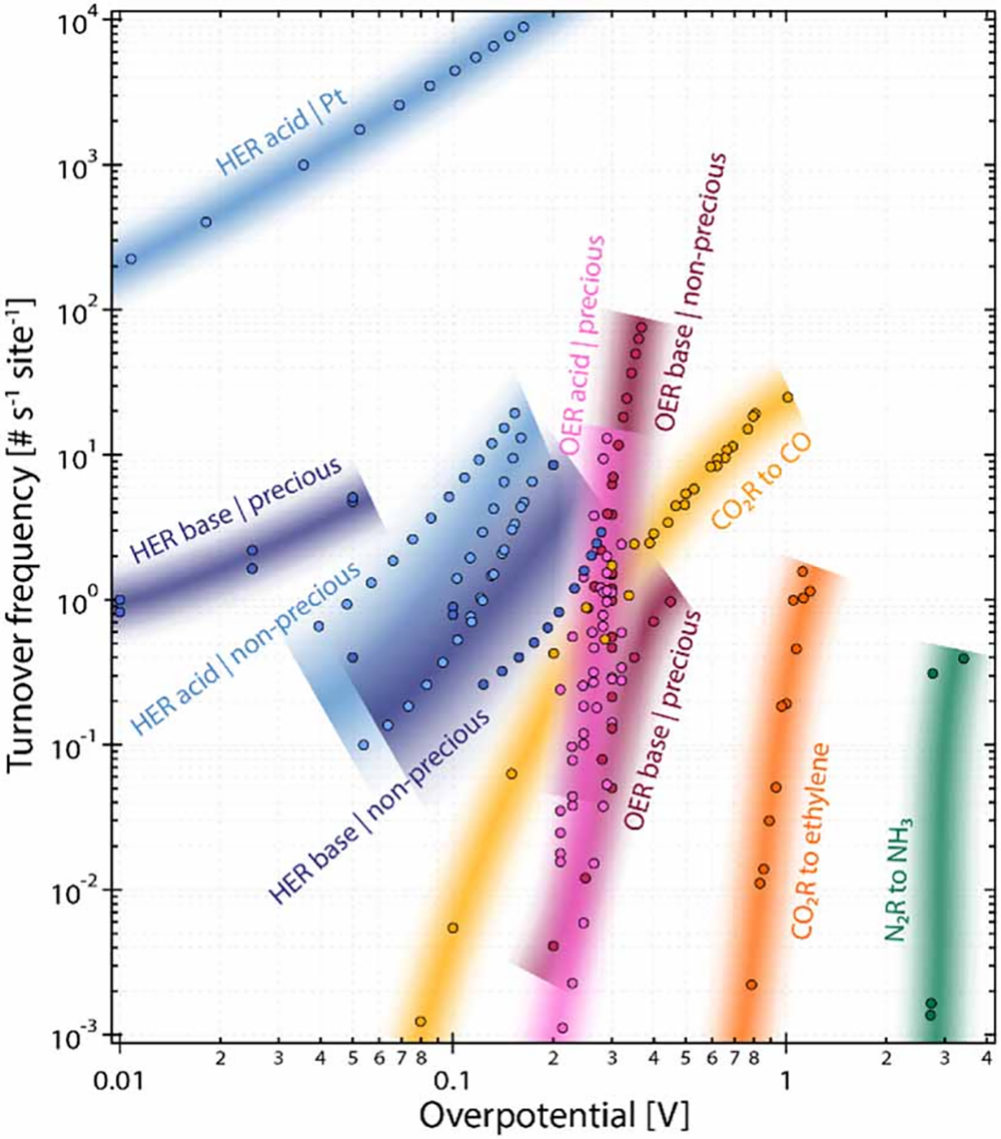
Turnover frequency (TOF) of state-of-the-art catalysts for CO_2_RR to CO, CO_2_RR to ethylene, and other significant electrocatalytic reactions plotted against the overpotential relative to the thermodynamically required potential for the respective reactions. Shadings are a guide to the eye. Reproduced with permission from ref 100. Copyright 2022 IOP Publishing Limited.

Addressing these intertwined issues requires more than next-generation polymers and ionomers with high conductivity, tunable hydration, and long-term stability. An emerging priority is the science of device integration, which focuses on materials compatibility and interface behavior under industrially relevant conditions. This perspective shifts how fundamental studies should be conducted: even in H-cells or half-cells, researchers must consider the actual catalyst–polymer electrolyte interfaces and the reactive species, gradients, and chemical potentials present in MEA-based devices. Designing basic science experiments keeping this in mind ensures that mechanistic insights translate more reliably to full devices.

Moreover, another frontier to focus on is the science of scale-up. Practical reactors introduce down-the-channel gradients, catalytic nonuniformities, reactant distribution effects, and variations in water activity and local chemical potential that are often absent in benchtop systems. These emergent behaviors strongly influence selectivity, stability, and transport. Developing systematic model platforms that reproduce these mesoscale conditions will be essential for identifying mechanisms that only arise at high current densities and industrially relevant scales. Together, these aspects will be central to bridging laboratory advances with commercial CO_2_ electrolysis technologies.

Low-temperature CO_2_ electrolysis has evolved from a catalyst-centric discipline into a chemistry-informed systems science. The shift from searching for active metals to understanding interfacial chemistry, ion transport, and polymer stability has yielded tangible gains in carbon efficiency, selectivity, and durability. The next transformative step will significantly depend on rationally designed, stable polymer electrolytes that can withstand the coupled electrical, chemical, and mechanical stresses of continuous operation. Solving this challenge will close the gap between laboratory discovery and commercial deployment for electrochemical CO_2_ conversion.

### 2.2 High-Temperature Electrolysis

High temperature electrolysis represents a fundamentally distinct pathway for electrochemical CO_2_ conversion, unique both in its elevated operating temperature—generally ranging from 500 to 1000 °C—but also in the underlying CO_2_ reduction chemistry.^101^ The origins of high temperature solid oxide electrochemical cell (SOEC) application in CO_2_ conversion are generally linked to work led by NASA beginning in the 1960s as a means of providing both life support and propellant in space.^102,103^ In the time since, SOEC utilization has expanded to more recently include CO_2_ conversion for applications in industrial chemicals and fuels production (i.e., Power-to-X).^104^

Shown in Figure 6, two primary reaction pathways have been considered for CO_2_ electrolysis in SOECs, depending on reactor design and operation. In oxygen-conducting configurations (O-SOECs) electrochemical reduction of CO_2_ is performed at the negatively charged cathode (fuel electrode), forming carbon monoxide (CO) and oxygen ions (O^2–^) shown in Equation 1. In the presence of H_2_O (i.e., coelectrolysis mode) H_2_O can similarly be converted to H_2_ and O^2–^ (Equation 2). The oxygen ions generated from reduction on the cathode subsequently migrate through a porous ceramic-based solid electrolyte that isolates and conducts ions between the two electrodes, recombining at the positively charged anode (oxygen electrode) to form molecular oxygen (O_2_, Equation 3).

**Figure 6:**
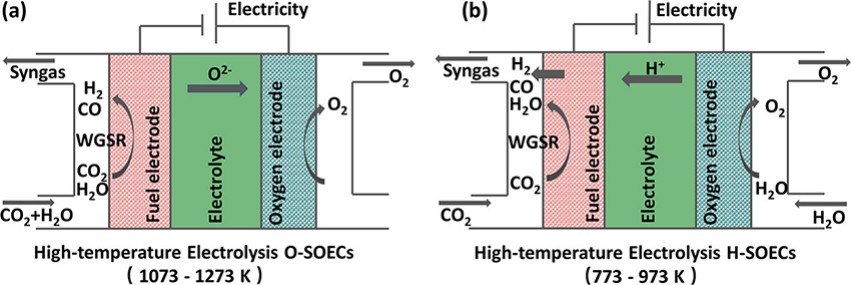
(a) Oxygen-conducting and (b) proton-conducting solid oxide electrochemical cells for CO_2_ conversion. Reproduced from ref 101. Copyright 2024 American Chemical Society.

Alternatively, in proton conducting SOECs (H-SOECs), H_2_O is exclusively fed to the anode whereby molecular oxygen and protons (H^+^) are formed (Equation 4). The generated protons then cross the electrolyte to the cathode, facilitating the reduction of CO_2_ to products. Unlike in O-SOEC operation where products are limited to CO for pure CO_2_ feeds or CO + H_2_ in the presence of H_2_O, the transfer of protons in H-SOEC combined with less extreme operating temperatures (500 – 700 °C) allows for a more diverse product set at the fuel electrode, including CO, CH_4_, H_2_O, and H_2_ depending on specific reaction conditions and water-gas shift activity (Equation 5). These SOEC-derived products, in particular syngas, are viewed as promising feedstocks for the chemical industry to support a variety of high-volume applications such as methanol synthesis, Fischer–Tropsch liquids, or other industrially vetted catalytic processes to generate liquid fuels and valuable chemicals.

1 $$CO_{2}+2e^{-}\to CO+O^{2-}$$

2 $$H_{2}O+2e^{-}\to H_{2}+O^{2-}$$

3 $$2O^{2-}\to O_{2}+4e^{-}$$

4 $$2H_{2}O\to O_{2}+4H^{+}+4e^{-}$$

5 $$CO_{2}+4H^{+}+4e^{-}\to CO+H_{2}O+H_{2}$$

As noted, a key defining attribute of SOECs is the elevated temperature regime at which they operate, offering a unique advantage in the way energy is sourced for CO_2_ conversion. Indeed, as highlighted by the thermodynamics of direct conversion of CO_2_ in Figure 7, although the total energy demand (i.e., enthalpy of formation ΔH_f_) for the conversion to CO is nearly constant across the typical temperature SOEC range, thermodynamic principles state that the energy can be supplied either in the form of electrical energy (Gibbs free energy of formation, ΔG_f_) or in the form of heat (TΔS) as shown in Equation 6.^30^ Since the entropic term ΔS is positive, as reaction temperature, T, increases, the amount of electrical energy required decreases proportionally. For example, at 25 °C the electrical energy component ΔG comprises approximately 90% of the total energy demand ΔH_f_, yet at 800 °C only 67%.^30^

**Figure 7:**
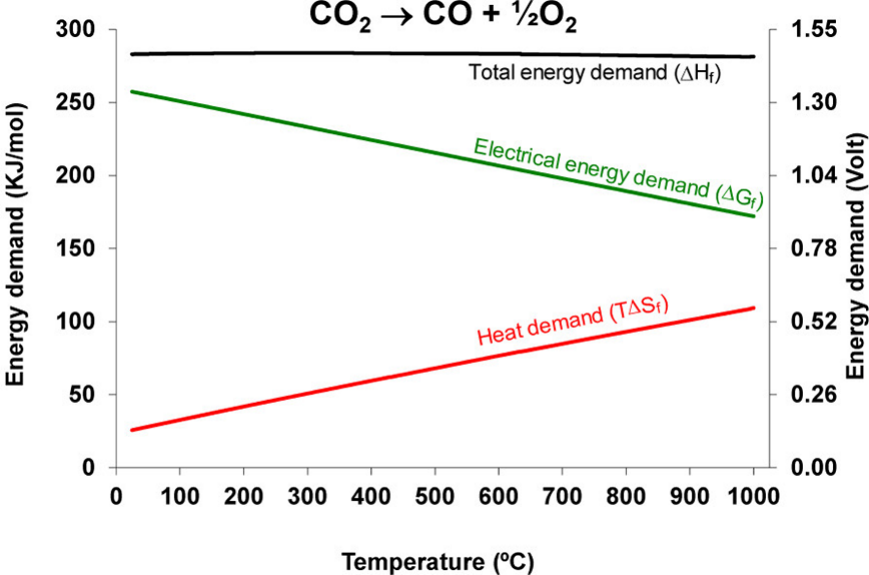
Thermodynamics of CO_2_ electrolysis as a function of temperature. Reproduced from ref 105. Copyright 2014 American Chemical Society.

Consequently, since heat can be significantly cheaper to source than electricity, this relationship between heat and electrical energy demand has important implications to SOEC operation and can be leveraged to the advantage of industry practitioners.^103^ Specifically, through utilization of the joule heat produced internally from ohmic resistances during electrolysis and/or opportunities in utilizing colocated waste process heat (e.g., from a refinery or nuclear power plant), electrical energy demand can be significantly reduced often without the need for on-purpose external heat. Leveraging these principles, CO_2_ conversion energy efficiency has been shown to exceed 95%,^105^ a significant advantage compared to low temperature processes that meet the entirety of the energy demand through external electrical inputs. Further, at these elevated temperatures, SOEC chemistry benefits from thermally enhanced reaction kinetics, reducing the overpotentials required to drive CO_2_ conversion at meaningful rates thereby improving productivity. However, a tradeoff of high temperature operation is C-C bond formation becomes thermodynamically unfavorable, translating to a product slate limited to only C_1_ products (e.g., CO, CH_4_) compared to the more diverse low temperature CO_2_ electrolysis product set comprising a variety of C_1_, C_2_ and C_3+_ products.^9^

6 ΔH=ΔG+TΔS

As the potential use cases and general interest in SOECs has expanded in recent years to include CO_2_ conversion for fuels and chemicals synthesis, the field has largely consolidated R&D efforts around two central themes: (1) materials development for high performance electrodes and electrolytes, and (2) understanding and overcoming degradation mechanisms to improve long-term system durability.^104^ Indeed, there is a large body of work and published reviews covering both topics falling outside the intended scope of this review. For a comprehensive summary of pre-2020 literature, we direct readers to the following reviews.^103–106^ Key takeaways from this early work across the three primary components of the SOEC (e.g., electrolyte, fuel electrode, oxygen electrode) and their respective stability are discussed below.

A functional electrolyte for SOECs must exhibit negligible electronic conductivity to prevent short-circuiting, high ionic conductivity to facilitate rapid transport of O^2–^ or H^+^ species, and strong chemical resistance to oxidizing and reducing environments. Additionally, electrolytes must also match the thermal expansion properties of the adjacent fuel and oxygen electrodes and possess sufficient mechanical strength to resist embrittlement and fracturing, all while being as thin as possible to reduce ohmic losses.^103^ Early testing of electrolyte materials and respective dopants has identified Z_r_O_2_ stabilized with Y_2_O_3_ or “yttria-stabilized zirconia” (YSZ) as the leading electrolyte choice in O^2–^ conducting O-SOECs. Specifically, earlier studies commonly cited electrolytes comprising a 8 mol% blend of Y_2_O_3_ with ZrO_2_ or (8YSZ), providing a strong blend of high ionic conductivity and performance across the target SOEC operating temperature range and harsh reducing environment.^105^ H-SOEC research has similarly consolidated around the use of barium cerate-zirconate (BCZY) based electrolytes and other metal doped variants to balance proton conductivity, chemical stability against CO_2_ and H_2_O, and mechanical strength.^105^

The purpose of the fuel electrode is to provide thermally stable active sites that facilitate the reduction of CO_2_ into value-added products. High-performing electrodes are typically comprised of porous media designed to facilitate the simultaneous transport of electrons, ions, and CO_2_ gas to active catalyst sites at the “triple phase boundary” (TPB)—the critical junction where these three phases intersect. The most common material architecture used in fuel electrodes is a metal ceramic composite known as a “cermet”, with Ni-YSZ representing the benchmark for much of the pre-2020 era. The widespread adoption of Ni-YSZ electrodes is motivated by its combination of synergistic properties including high electronic conduction without the use of rare earth metals, high activity toward C = O bond cleavage, and rigid structural properties. Indeed, the use of cermets is a key to the mechanical stability of the entirety of the electrode stack as the ratio of metal to ceramic can be tuned to match the thermal expansion coefficient of the electrolyte, minimizing thermal stress and risk of delamination or cracking during operation and cycling.

On the oxygen electrode, materials compatible with high operating temperatures in a highly oxidizing environment are required. Most early studies investigated the use of mixed-conducting ABO_3‑δ_ perovskites.^105^ The ABO_3‑δ_ nomenclature contains key structural information about the perovskite materials and its electronic properties. Specifically, the A-site is characterized by a large-radius ion coordinated by 12 oxygen ions, often comprised of elements La^3+^, Sr^2+^, or Ba^2+^. The B-site cation is smaller in radius and coordinated by 6 oxygen ions usually comprised of a transition metal ion like Co^3+^, Fe^3+^, or Mn^3+^.^107^ The “O” term denotes oxygen sites in the crystal structure and -δ reflects the oxygen nonstochiometric nature (i.e., intentional oxygen vacancies) created by substituting ions of different valency into the lattice structure.

These oxygen vacancies are deliberately induced during synthesis to create the highly desirable property of mixed ionic and electronic conductivity (MIEC). Unlike the Ni-YSZ cermets on the fuel electrode which is a composite of separate electronic and ionic conducting phases, the MIEC ABO_3‑δ_ perovskite does both in a single material. This significantly expands the electrochemical reaction zone beyond the TPB to instead effectively cover the entire surface of the electrode.^104^ Commonly cited oxygen electrode compositions include lanthanum-based materials such as lanthanum strontium manganite (LSM) or lanthanum strontium cobalt ferrite (LSCF), both known for their high catalytic activity for oxygen evolution and compatibility with the electrolyte.^108,109^ In applications involving LSCF, a small buffer layer of gadolinium-doped ceria (GDC) is also typically inserted as a thin reaction barrier between the YSZ electrolyte and the oxygen electrode to prevent unwanted reactions between the two layers at high temperature.^104^

Despite the relatively high initial performance of these early benchmark materials (e.g., Ni-YSZ | YSZ | GDC-LSCF), fundamental challenges surrounding long-term stability and degradation have slowed commercialization and scale-up. While the baseline Ni-YSZ fuel electrode demonstrated excellent conduction properties, the high partial pressures of CO present on the fuel electrode in turn made it prone to carbon deposition (coking) from the Boudouard reaction (2CO ⇄ C_(S)_ + CO_2_).^110^ Further, at temperatures required to maintain conductivity (e.g., 800 – 850 °C), nickel was also shown to be susceptible to redox instability leading to particle aggregation and “coarsening”.^111^ Both phenomena, coking and aggregation, contribute to the blocking of TPB active sites and progressively deteriorate the performance of the SOEC over time. Indeed, the fuel electrode has been shown to be the primary source of degradation across SOEC experiments.^107,110^

On the oxygen electrode, high partial pressures of gases (e.g., oxygen) have similarly been linked to stability issues whereby O_2_ bubble formation at the electrolyte-electrode interface was observed to lead to delamination and long-term irreversible damage and increase in ohmic resistance.^103^ Additionally, during long-term stability studies cation migration in the perovskite structure and formation of secondary insulating phases were shown to further dampen performance. These issues combined with stack-level challenges of accumulation of contaminants (e.g., Cr, Si, B, S),^103^ interconnected corrosion, glass crystallization has motivated the next phase of SOEC research and shift from emphasis on initial performance metrics to sustained long-term durability.^104^

In attempt to address some of these noted challenges, recent advancements (2020–2025) have emphasized materials development as a means to overcome performance limitations and tradeoffs between catalytic activity vs stability. Specifically, in the benchmark Ni-YSZ fuel electrode, the metallic and ceramic phases are physically mixed as a composite resulting in metal particles adhered to the surface of the support material via weak physical interactions. As revealed by earlier studies, this form of physical metal–support interaction and lack of strong bonding leads to high surface mobility of nickel and eventual aggregation at high temperatures.^105,112^ Consequently, recent research has investigated alternative cathode materials with enhanced long-term stability. Perovskite oxides—like those used on the oxygen electrode—have gained attention as a potential replacement for Ni-YSZ due to their excellent stability, structural tunability, and thermal compatibility with commercial electrolytes.^113^ However, the use of perovskites alone is hindered by their intrinsically low catalytic activity toward CO_2_ reduction relative to the metallic nickel baseline.^113,114^ To overcome this activity-stability tradeoff, emerging cathode development has centered around different pathways for modifying the parent perovskite framework to increase the number of reaction sites and their activity toward CO_2_ conversion. These efforts typically fall within three categories: lattice doping, composites of two or more electrode materials, or surface engineering modifications (see: Figure 8).^107^

**Figure 8:**
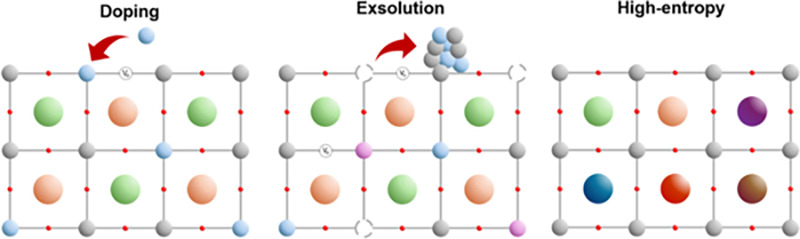
Illustrations of perovskite enhancements via doping, exsolution, or high-entropy synthesis. Adapted with permission from ref 107. Copyright 2025 John Wiley and Sons.

Perovskite doping is the intentional exchange of ions within the parent lattice. A-site doping involves the substitution of A-site cations (e.g., La^3+^) with divalent cations (e.g., Ca^2+^, Ce^2+^, Sr^2+^) to change the local valency within the crystal lattice and drive the creation of oxygen vacancies to maintain charge neutrality.^115^ A common example is the transition of LaFeO_3_ → La_1‑x_Sr_x_FeO_3_ (LSF) where a fraction (x) of La^3+^ is replaced with Sr^2+^. Not only do oxygen vacations play a critical role in CO_2_ conversion chemistry by providing sites for CO_2_ adsorption and conversion to CO but depending on the specific dopants used, ionic conductivity, thermal stability, and durability properties may also be enhanced (see: Figures 9 and 10). Similarly, B-site doping is the deliberate substitution of catalytically active transition metals (e.g., Ni, Fe, Co, etc.) into the B-site of the perovskite crystal lattice to maximize electronic conductivity and provide the necessary active sites to drive CO_2_ reduction chemistry.

**Figure 9:**
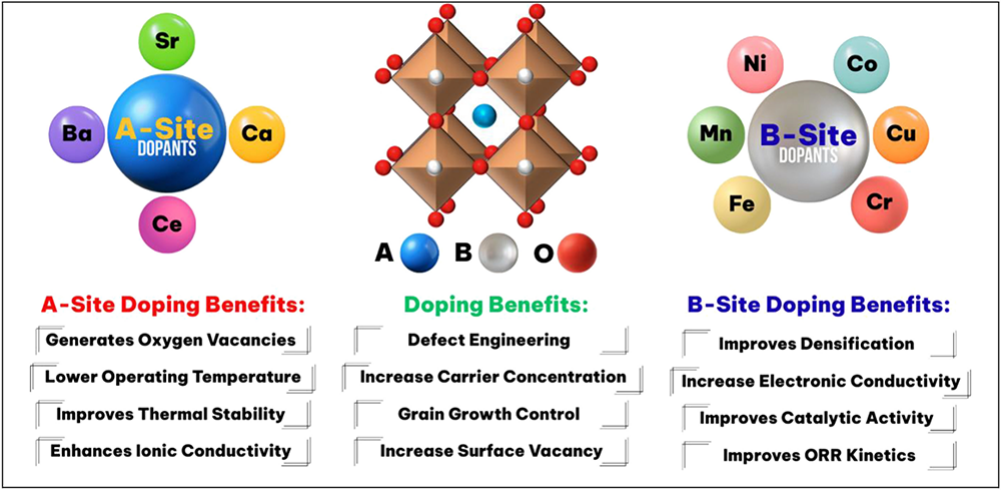
A-site and B-site doping of ABO3 perovskites in SOEC electrodes. Reproduced with permission from ref 115. Copyright 2026 Elsevier.

**Figure 10:**
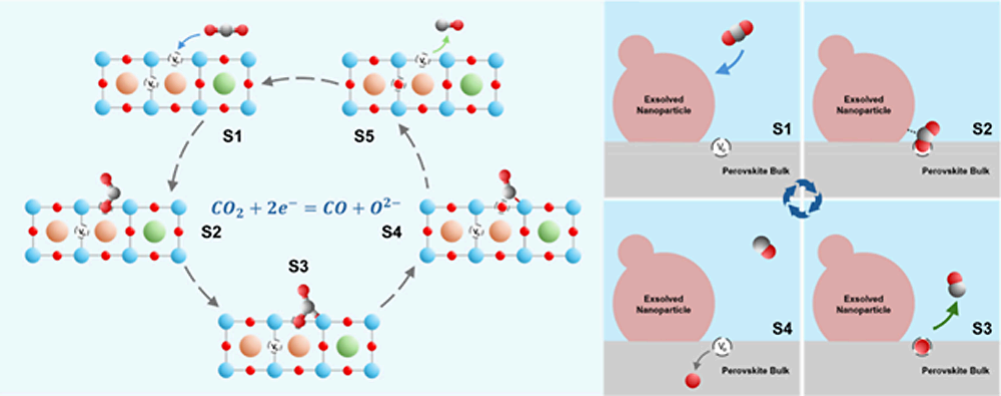
Mechanisms of CO_2_ conversion over exsolved-based perovskite cathodes. Reproduced with permission from ref 107. Copyright 2025 John Wiley and Sons.

In an extension of traditional A/B-site doping strategies, other emerging research has focused on the development of “high-entropy perovskites” (HEP) as another means of overcoming the stability-activity tradeoffs of conventional electrodes. Unlike in conventional doping where a relative minor fraction of the host lattice undergoes cation substitution (e.g., La^3+^ for Sr^2+^), HEPs incorporate multiple cations (5+) at near equimolar ratios to induce radical changes inside the host lattice and maximize configuration entropy (Figure 8). Increasing the lattice entropy acts to thermodynamically stabilize the perovskite structure, suppressing the formation of secondary phases or cation migration at elevated temperatures that are often observed in lower entropy configurations. Further, in addition to enhanced stability, the presence of multiple cationic species and the associated lattice distortion contributes to a diverse set of active sites that facilitates CO_2_ adsorption and reduction to CO.^116^ Recent promising examples of materials tested for CO_2_ conversion include Sr_2_Fe_1.0_Ti_0.2_Cr_0.2_Mn_0.2_Mo_0.2_Ni_0.2_O_6‑δ_,^116,117^ La_0.2_­Sr_0.2_­Pr_0.2_­Ba_0.2_­Ca_0.2_­FeO_3‑δ_,^118^ and La_0.2_­Pr_0.2_­Sm_0.2_­Sr_0.2_­Ca_0.2_­Fe_0.9_­Ni_0.1_­O_3‑δ_,^119^ reporting current densities in excess of 1.5 A cm^–2^ while also showing durability in excess of 100h.

As a complement to these bulk lattice modification strategies, active research has also been devoted to developing “exsolution” synthesis techniques to modify the perovskite surface structure for long-term stability. Specifically, if A/B-doped perovskites are treated with a reducing atmosphere (e.g., H_2_, CO), the formation of oxygen vacancies destabilizes the perovskite framework, causing the reducible metal ions (e.g., Ni^2+^) at the B-sites to migrate from the bulk to the surface where they undergo chemical reduction into their metallic state (e.g., Ni^2+^ → Ni^0^) to maintain thermodynamic equilibrium in the lattice (Figure 10). With the metal particles “growing” from within the lattice, they become effectively socketed and chemically anchored within the perovskite skeleton thereby strengthening the integrity of the TPB. This is fundamentally different and a critical distinction from conventional synthesis techniques whereby the metal nanoparticles are more weakly bound on the surface. Thus, the exsolution method offers a pathway to preserve the high initial catalytic activity of the metal while suppressing a known degradation mechanism.^120^

Indeed, as highlighted by Han and co-workers in Figure 11,^107^ activity in perovskite-based fuel electrodes has garnered significant interest in recent years. While research is still ongoing to pinpoint the optimal combinations of A/B-site dopants, material blends, and surface modifications, data published to date paints an optimistic picture for overcoming the initial activity-stability tradeoff issue. A compilation of reported data highlights relatively low impedance of doped perovskites is possible at commercially relevant current densities while also showing stability in lab scale testing in excess of 100h.^107^

**Figure 11:**
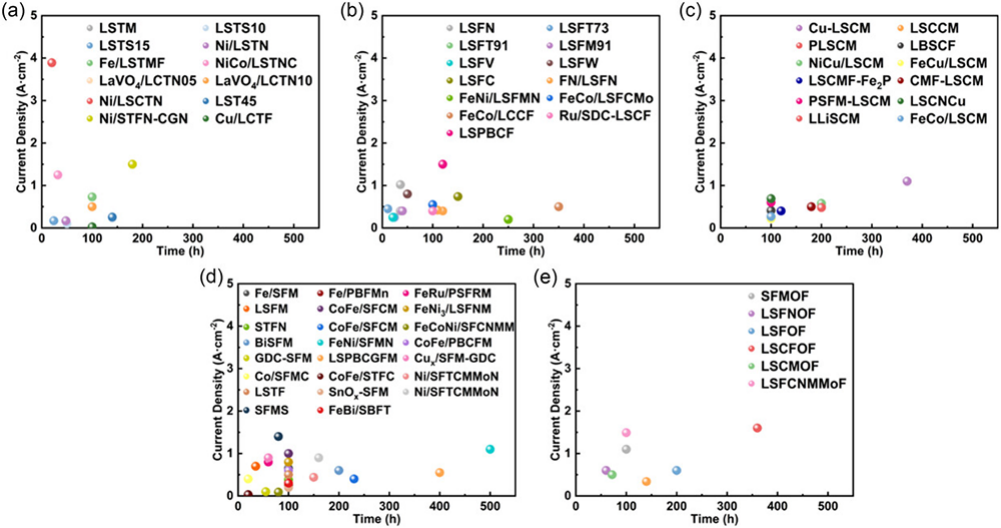
Electrochemical stability summary of (a) LST-based perovskites, (b) LSF-based perovskites, (c) LSCM-based perovskites, (d) double perovskites, and (e) anion-site modified cathodes. Reproduced with permission from ref 107. Copyright 2025 John Wiley and Sons.

Although SOEC energy efficiency remains excellent and is a standout across CO_2_-conversion pathways, further future research is needed. Despite these recent strides in perovskite tuning and initial performance gains, the mechanistic link between dopant chemistry, lattice distortion, vacancy energetics, and long-term kinetics remain poorly understood and should be a focus of ongoing R&D.^115^ Extended long-term stability testing beyond the 100–300 h commonly cited and into the thousands of hours will be another critical next step to demonstrating the potential for these novel materials in commercial applications. Advances in in-situ characterization techniques and computational modeling will be essential in guiding the rational design of next generation solid oxide electrolysis cells. With continued innovation to develop more robust and cost-effective materials, improved reactor designs, and integrating SOEC systems with existing industrial processes to test systems under real-world conditions, high temperature CO_2_ electrolysis has the potential to become a key technology for sustainable carbon utilization, enabling the production of renewable fuels and chemicals while contributing to global decarbonization efforts.

### 2.3 Non-Thermal Plasma

Nonthermal plasma (NTP) technologies provide an electron-mediated approach to activate and convert the stable CO_2_ molecule. NTP is generated by applying an electromagnetic field to a gas which energizes electrons that activate ground-state gas molecules through excitation, ionization, and dissociation processes.^121^ The resulting partially ionized gas includes reactive species such as electrons, ions, radicals, and vibrationally and electronically excited species that can promote thermodynamically and kinetically unfavorable gas-phase and surface reactions under conditions far from those accessible by conventional thermal catalytic chemistry.^122^ NTP is characterized by its deviation from local thermodynamic equilibrium, wherein the electron temperatures are higher than those of the bulk gas ions and neutral species. Consequently, NTP is exceptionally suited for driving thermodynamically unfavorable reactions of CO_2_ at more mild conditions. The highly energetic electrons (1–10 eV range) can break the C = O bond or reduce energy barriers for reaction without substantially heating the bulk gas as in thermal methods or requiring other reactants, although additional reactants can lead to dramatic changes in chemistry as will be discussed in Section 3.2. In this contribution, we will focus on NTP, from cold to warm discharges, rather than thermal plasmas that are in local thermodynamic equilibrium, to take advantage of the deviations from equilibrium and the interesting chemistry that arises, as well as to enable integration with a catalyst to promote selectivity within the chemical pathways.

Depending on the electrode geometry and configuration as well as power source, multiple NTP reactor types can be created including dielectric barrier discharges (DBDs), gliding arc (GA), and microwave (MW) plasmas which are most studied for CO_2_ conversion, although other types have been investigated as well (e.g., radio frequency, atmospheric pressure glow, corona, spark, and nanosecond pulsed discharges).^123^ Catalysts are readily integrated within the plasma zone of DBD reactors that have gas temperatures ranging from 300 to 400 K,^124^ enabling increased selectivity control through plasma catalysis, although, despite high conversion, the energy efficiency of DBD plasmas is lower compared to other plasma systems such as GA or MW.^125^ These reactors, while still considered nonthermal due to their nonequilibrium nature, have higher average gas temperatures ranging from 1000–3000 K for MW and 1000–1500 K for GA,^124^ which affect CO_2_ reactivity. MW and GA also have extreme temperature gradients, with core temperatures that can exceed 6000 and 3000 K, respectively, in the core region, with significantly cooler temperatures in the afterglow region.^126,127^ The details of these NTP systems in reference to CO_2_ conversion have been extensively covered in the literature,^125,128–132^ so here we will focus on conveying key aspects salient to understanding the chemistry of NTP CO_2_ conversion. For direct CO_2_ splitting, there are three primary plasma-mediated reaction pathways. These processes are covered in greater detail elsewhere,^133^ but they include (i) CO_2_ splitting by electronic excitation which is not very efficient due to the mismatch between the lowest electronic level of CO_2_ and the dissociation enthalpy of CO_2_ (5.5 eV), (ii) CO_2_ splitting via vibrational excitation which is more energy efficient, and (iii) CO_2_ splitting by thermal dissociation at high plasma temperature. Electronic excitation is prevalent in DBD processes with the proportion of vibrationally excited species increasing in warmer plasmas such as GA or MW, due to different reduced electric fields (i.e., electric field divided by gas number density (E/N), expressed in Td) of the different discharge types. Typical E/N values in DBDs are above 100 Td, yielding electron impact dissociation, while vibrational excitation requires lower E/N (below 50 Td), like in warm plasmas.^134^ While the energy efficiency of CO_2_ splitting by vibrational excitation is higher than that of splitting by electronic excitation, the higher temperature of plasmas with larger vibrationally excited populations can change the options for integration with materials such as catalysts which have the potential to improve the selectivity of NTP catalysis processes,^134–137^ especially those with additional reactants, as will be discussed in Section 3.2. An example of the reaction pathways for CO_2_ splitting based on kinetic modeling by Mohanan et. al is shown in Figure 12 for a gas phase discharge at 300 K, with two different specific energy inputs (SEIs), demonstrating how much influence energy input can have on the reaction rates of different conversion pathways. Mohanan^138^ also showed how reaction pathways are modified at higher gas temperatures (up to 1050 K) highlighting the differences in mechanisms between cold and warm discharges.

**Figure 12:**
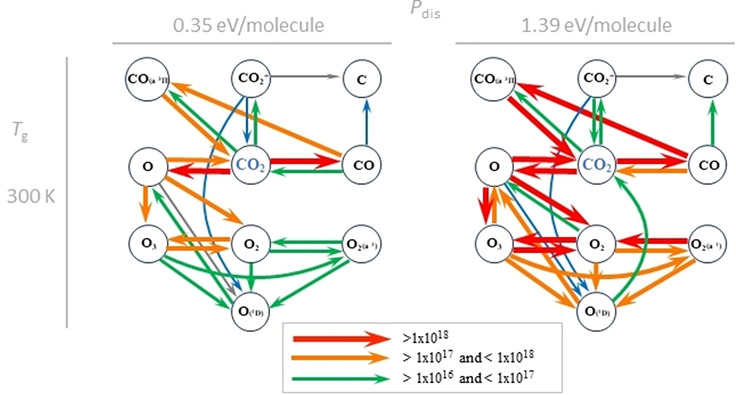
CO_2_ conversion pathways in an NTP reactor at 300 K at two different input energies, with production pathway magnitudes as gray < blue < green < orange < red. Reproduced with permission from ref 138. Copyright 2024 Chemistry Europe.

Many excellent studies have evaluated the impact of operational parameters such as reactor configuration, electrode material, dielectric material, discharge gap, applied frequency, applied power, gas flow rate, dilution gas (e.g., Ar, He), and reactor temperature and pressure on NTP-assisted CO_2_ splitting and others have highlighted the role of packing media including catalysts, and these are collectively reviewed and discussed in the literature.^137,139,140^ Integration of catalyst materials in DBD reactors is of particular interest as it provides opportunities to overcome the inverse relationship between energy efficiency and conversion and enhance both concurrently,^141^ for example by providing stable surface oxygen vacancies in Mo-doped CeO_2_ that can promote CO_2_ dissociative adsorption to improve conversion.^142^ Gao, et al., describe the integration of a NiCo-CuO catalyst into a DBD plasma system to enhance the uniformity of the discharge and the energy distribution of discharge filaments, highlighting the impact of the catalyst material on the plasma environment.^143^

Warm plasmas, including GA and MW discharges, are capable of much higher conversions and energy efficiencies, initially ascribed to the primary CO_2_ vibration splitting pathway. More recently, it has been suggested that the high temperatures and accompanying thermal CO_2_ decomposition rather than electron-mediated reactions drive the high efficiencies.^138^ However, vibrational excitation may be important for very low pressure MWs at limited conversions.^144^ Nonetheless, some researchers argue that particular reactor geometries, such as a modified GA with vortex flow (GA plasmatron) have reached conversion and energy efficiency limits.^145^ Therefore, strategies for further performance improvements include limiting back reactions to CO_2_.^133^ In contrast to in-plasma catalysis that is suitable for the lower temperatures of DBD reactors, catalyst integration directly in the warm (1000 K+) plasma zone is not compatible with traditional catalyst materials, as the high temperatures can cause catalyst deactivation.^132^ However, materials that can quench back reactions by reacting oxygen species with a postplasma carbon bed or reduced metal oxide may be introduced in the postplasma region to improve the conversion and efficiency.^146^ For example, Girard-Sahun et. al demonstrated nearly double conversion (7.6% to 12.6%) with energy efficiency increasing from 27.9% to 45.4% by introducing a postdischarge carbon bed in a GA plasmatron system.^147^ Other approaches to limit back reactions in warm plasmas include quenching the high temperature gas,^148,149^ the utilization of pulsed systems,^150^ or the integration of membranes.^151,152^

While progress has been made in improving conversion and efficiency of NTP based CO_2_ splitting, there are still many challenges to scale these reactors to higher TRL. While details are provided in Figure 20 and Figure 21, the primary challenges faced by cold DBDs and warm GA/MW are inherently different due to the various CO_2_ dissociation mechanisms (electronic vs vibrational). Additionally, the opportunities to integrate (catalyst) materials directly into the plasma are different due to the local temperatures produced. Researchers have pointed out that while improvements through geometry modifications or catalyst integration could be impactful, they are not necessarily critical to commercial success given that plasma processes may not compete in the same markets as existing large-scale processes as they are more suited to modular distributed systems.^153^ However, another promising avenue for NTP upgrading of CO_2_ targets higher value products such as C1 hydrocarbons. These products are formed in conjunction with secondary reactants such as H_2_, hydrocarbons, or water and will be discussed in Section 3.2.^125,128^

### 2.4 Photochemical Reduction

Photochemistry provides a distinct route to CO_2_ conversion by using photons to access excited-state pathways that are not available under purely thermal or electrochemical conditions.^154^ Light absorption enables these catalysts to stabilize certain reactive intermediates that steer selectivity, opening novel routes to value-added fuels and chemicals—including emerging C_2_ + products^155,156^ —powered by photons. Light can further be used to lower apparent activation energies,^157^ allowing reactions to proceed under comparatively mild conditions—an especially valuable advantage for endothermic processes such as dry reforming of methane or the reverse-water gas shift (RWGS). Recent years have shifted the field from broad materials exploration toward a sharper focus on structure–property relationships, defect chemistry, and microenvironment design that enable selective, efficient, and durable CO_2_ conversion.

At its core, photochemical CO_2_ conversion couples light absorption with catalytic chemistry that enable photons to activate charge carriers rather than thermally excite reactants. In practice, this typically occurs in liquid slurries or gas–solid photoreactors, where for example a semiconductor or plasmonic catalyst is irradiated by simulated or natural sunlight. In liquid-phase systems, CO_2_ is sparged or dissolved into an aqueous or mixed solvent containing a suspension of photocatalyst powder, allowing photogenerated electrons and holes to drive reduction and oxidation half-reactions at the solid–liquid interface. In gas-phase systems, CO_2_ flows over a fixed or fluidized photocatalyst bed under illumination, often co–fed with H_2_ or CH_4_.^158^ Photoelectrochemical (PEC) cells combine light absorption with applied bias. Many incorporate membrane-separation analogous to electrolyzers but are driven primarily by sunlight rather than electricity. Across all architectures, the key commonality is the generation of photogenerated charge carriers that migrate to the catalyst surface to activate and reduce CO_2_, often through intermediates such as *CO_2_^–^, *COOH, or *CO.

The mechanistic foundations of photocatalytic CO_2_ reduction were firmly established well before 2020, encompassing several major classes of materials. Classical semiconductors such as TiO_2_, SrTiO_3_, g-C_3_N_4_, and GaP operate by promoting electrons from the valence to the conduction band upon light absorption, where photoexcited electrons reduce adsorbed CO_2_ and holes drive complementary oxidation reactions (Figure 13a).^159,160^ Single-atom catalysts based on transition-metal complexes and photosensitizer–catalyst dyads,^161^ in both homogeneous and heterogeneous forms, offer precise control over coordination environments and have proven highly effective in tuning product selectivity.^162^ More recently, plasmonic nanostructures, particularly those based on Au, Ag, and Cu, have gained attention for their exceptional ability to harvest light and open new activation channels through localized surface plasmon resonance (LSPR)—the collective oscillation of conduction electrons under optical excitation. These pathways include hot-carrier injection into antibonding orbitals of adsorbed CO_2_, near-field effects that can induce carrier separation at interfaces, and multielectron transfer accelerated by photothermal heating (Figure 13b). Although the relative contributions of these effects remain challenging to deconvolute experimentally,^163^ their interplay can produce reactivity and selectivity patterns distinct from both thermal catalysis and conventional semiconductor photocatalysis.^164^

**Figure 13:**
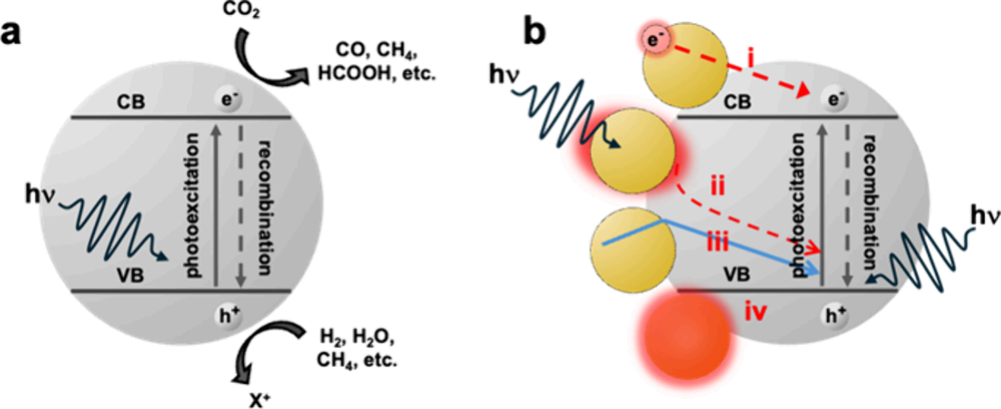
(a) Activation of a semiconductor photocatalyst. (b) Activation of a semiconductor with plasmonic metal NP. Potential mechanisms driving plasmon-mediated semiconductor photocatalysis include (i) hot electron transfer, (ii) near-field enhancement, (iii) resonant scattering, and (iv) local heating. Adapted with permission from ref 165. Copyright 2025, Springer Nature.

What has evolved since 2020 is not a wholesale reinvention of the field, but rather a continued refinement of the underlying chemistry and an ever-sharper ability to pinpoint reactive intermediates and mechanisms that drive selectivity. For example, in semiconductor photocatalysts, defect chemistry has emerged as a deliberate design lever. Oxygen vacancies in metal oxides—once viewed as detrimental—are now recognized for trapping photogenerated electrons, promoting charge separation, and suppressing recombination.^166^ Although full reduction of CO_2_ to CH_4_ is thermodynamically favored, kinetic limitations often bias selectivity toward partial reduction and CO desorption. Electrons stabilized at TiO_2_ oxygen vacancies, however, were recently observed to persist for *minutes* after illumination, extending their lifetime for multielectron transfer and substantially enhancing methane production.^167^ A related study coupled oxygen-deficient TiO_2_ with gold quantum dots to enable the oxidative coupling of methane (OCM) using CO_2_ as a soft oxidant.^168^ Mechanistic analysis suggested that CO_2_ adsorbed at Au–V_0_–Ti interfacial sites through dual Au–C and Ti–O coordination simultaneously facilitated CO_2_ reduction to *COOH while also lowering the barrier for C–H bond activation in methane. This synergy between CO_2_ activation and methane oxidation illustrates how tailoring defect and interface chemistry can direct complex photoredox cycles.

In recent years, efforts to exert finer chemical control over photocatalytic microenvironments have intensified, with covalent^169^ and metal–organic frameworks^170^ (COFs and MOFs) emerging as versatile platforms for integrating light absorption, charge transport, and catalysis within a single architecture. Their modular composition enables systematic variation of linker conjugation, metal–ligand geometry, and pore polarity—parameters that directly influence CO_2_ adsorption strength, charge separation, and intermediate stabilization. Single-atom^171^ and bimetallic sites^172^ confined within these frameworks have proven especially powerful for probing structure–function relationships: changes in coordination number or ligand field can shift reduction potentials, stabilize key *COOH or *CO intermediates, and steer selectivity toward CO, formate, or methanol. Operando spectroscopy and DFT studies increasingly show that site symmetry and electronic coupling between light-harvesting linkers and catalytically active nodes control the balance between electron delocalization and localization, defining both activity and product distribution. Collectively, this growing body of work demonstrates how tailoring local coordination environments and framework electronic structure provides a rational, molecular-level route to tune photocatalytic reactivity and selectivity.^173^

A major advance in photocatalytic CO_2_ reduction—particularly in tunability and scalability—has been the emergence of heterometallic plasmonic antenna–reactor (AR) architectures.^174^ In these systems, earth-abundant plasmonic “antenna” nanoparticles (e.g., Cu, Al) harvest light and transfer the resulting electromagnetic energy or hot carriers to adjacent catalytic “reactor” sites (e.g., Ru, Pd, Ni) that mediate bond activation (Figure 14).^175^ By decoupling light absorption from active-site chemistry, the AR concept enables independent optimization of optical and catalytic properties—a key step toward rational photocatalyst design. Mechanistically, the plasmonic antenna concentrates the local electromagnetic field and generates energetic charge carriers that migrate to nearby metal or oxide reactor sites, where they drive reactions such as CO_2_ reduction, C–H activation, and H_2_ evolution. In addition to promoting activity, these energetic carriers can also enhance catalyst stability by facilitating the desorption of poisoning species and mitigating sintering—limitations that often plague thermocatalytic systems.^176^ Representative Cu–Ru AR catalysts, for example, have demonstrated selective light-driven dry reforming of methane (CH_4_ + CO_2_ → 2CO + 2H_2_) with near-quantitative syngas yields and minimal coking under conditions where purely thermal operation typically deactivates and consumes H_2_ via RWGS.^177^ The resulting light-assisted reforming cycle cleaves C–H bonds more efficiently, continuously removes surface carbon, and channels photogenerated electrons into productive syngas formation rather than unselective side reactions.

**Figure 14:**
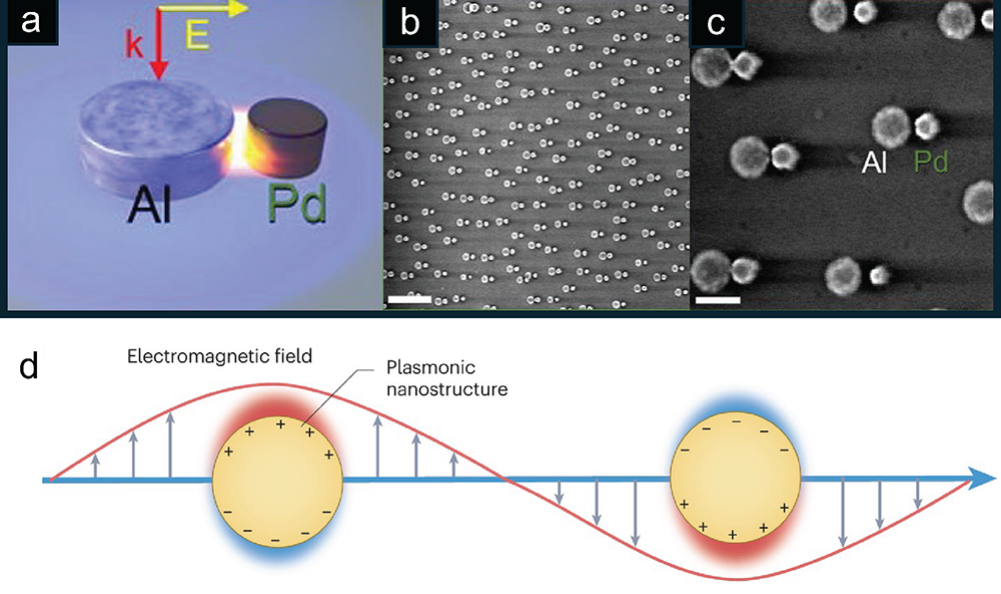
Planar nanodisk heterodimer complexes for plasmonic photocatalysis. (a) Schematics of forced plasmon modes excited on Al–Pd dimer; red and yellow arrows represent the wave vector and electrical field of the incident light, respectively. (b,c) SEM images of Al–Pd heterodimers, all with small gaps (∼5 nm on average) between the Al antenna and Pd reactors. Scale bars: (b) 500 nm and (c) 100 nm. Reproduced from ref 175. Copyright 2024 American Chemical Society. (d) Excitation of surface plasmons gives rise to near-fields in a plasmonic nanoparticle. Adapted with permission from ref 178. Copyright 2023, Springer Nature.

The same principles have been extended to other transformations of CO_2_, including methanation,^179^ RWGS,^180,181^ and selective C_2_ product formation.^182^ Across these chemistries, AR systems consistently demonstrate higher selectivity and lower onset temperatures compared to their thermal counterparts. Beyond the laboratory, these advances have translated to early pilot demonstrations: industrial-scale photoreactors employing AR catalysts have achieved continuous syngas production and ammonia-cracking operation at throughputs exceeding 200 kg day^–1^,^183^ underscoring the potential of AR designs as a scalable route to light-driven chemical manufacturing.

Coupling plasmonic photocatalysis with an applied electrochemical bias in photoelectrochemical (PEC) configurations merges the advantages of photochemical excitation and electrocatalytic control. By integrating optical energy input with electronic driving force, these hybrid systems lower kinetic barriers while maintaining steady-state currents and tunable redox environments. In CO_2_ electroreduction (CO_2_ER), sluggish CO_2_ activation and high overpotentials often limit efficiency, while competing hydrogen evolution (HER) suppresses selectivity. Plasmonic excitation directly addresses these challenges by generating energetic hot electrons that populate antibonding orbitals of *CO_2_, thereby reducing the barrier for *CO_2_•^–^ formation and accelerating proton-coupled electron-transfer (PCET) steps.^184^ Under constant potential, illumination can modulate product distributions—shifting selectivity toward deeper reduction pathways such as methanol^185^ or favoring C_2_^+^ coupling reactions otherwise inaccessible under dark electrolysis conditions.^186^

Beyond performance gains, these light-biased electrochemical systems also serve as a mechanistic bridge between purely photochemical and electrocatalytic regimes, allowing direct interrogation of photoinduced charge-transfer dynamics under controlled potential.^187^ Collectively, PEC platforms highlight how optical excitation can be used not only to reduce overpotentials and expand product scope, but to actively shape electron-transfer kinetics and interfacial energetics—providing a unifying framework for integrating light harvesting with catalytic selectivity control.

Taken together, these advances mark a decisive maturation in our molecular understanding of photocatalytic CO_2_ conversion. Over the past few years, increasingly sophisticated mechanistic tools—spanning ultrafast spectroscopy, operando surface probes, and theory-driven modeling—have illuminated how light absorption, charge transfer, and catalytic chemistry can be coherently coupled to steer reactivity. This deeper insight has begun to translate into measurable improvements: higher apparent efficiencies, tunable product distributions through defect and interface engineering, and mechanistic data sets now guiding machine-learning–assisted discovery.^188^ At the same time, early pilot demonstrations indicate that photochemical systems are beginning to transition from laboratory-scale discovery toward practical, light and solar-driven chemical manufacturing.

Still, fundamental chemical challenges persist. At the heart of photocatalytic CO_2_ conversion lies the need to orchestrate multielectron, multiproton transfer reactions across heterogeneous interfaces where carrier lifetimes are short and recombination pathways numerous. Disentangling the intertwined roles of hot carriers, photothermal effects, and defect states under dynamic illumination remains a major frontier, as these processes can evolve on ultrafast timescales and shift with surface reconstruction. Precise control over band alignment, and interfacial dipoles is required to modulate electron transfer energetics and suppress side reactions such as hydrogen evolution or partial CO desorption. Equally critical is understanding how local coordination, adsorbate geometry, and solvation can shape reaction intermediates—from *CO_2_^–^ and *COOH to C–C coupling motifs—and how these evolve under real operating potentials and photon flux. Catalyst deactivation through oxidative passivation or metal migration continues to constrain durability in real world systems, while structure–property relationships governing long-term photochemical stability are only beginning to be understood. Addressing these issues will continue to require deep integration of *in situ* spectroscopy, electronic-structure theory, and synthetic chemistry to establish quantitative correlations between surface composition, excited-state dynamics, and product selectivity.

### 2.5 Microbial Electrosynthesis

Microbial electrosynthesis (MES) is a hybrid bioelectrochemical technology that harnesses the metabolic capabilities of electroactive microorganisms to convert electrical energy and carbon dioxide into value-added organic compounds (for extensive review, please refer to (Rabaey, 2010).^189^ Operating at the interface of microbiology, electrochemistry, and environmental engineering, MES offers a sustainable platform for carbon capture and conversion, with potential applications in renewable energy storage, bioremediation, and biomanufacturing. MES employs chemolithoautotrophic microbes, often from the *Geobacter* or *Clostridium* genera, coupled with electrical energy to enable cathodic CO_2_ reduction into target products, ranging from short chain fatty acids (SCFAs) to alcohols.

MES has conventionally faced several key challenges that currently limit its scalability and efficiency, and in turn, economic viability and sustainability. From a biological perspective, the mechanisms of direct and mediated extracellular electron transfer (EET) at the cathode – wherein cells accept electrons from the cathode via physical contact using outer membrane redox proteins (e.g., cytochromes), conductive appendages (“nanowires”), or redox active biofilm matrices (Figure 15)– are slow, due to low exchange current densities at biocathodes, limited turnover of redox enzymes, poor biofilm conductivity, long electron transport distances through heterogeneous biofilms, mass transfer constraints for CO_2_ and nutrients, and/or competition with abiotic reactions. Further, these mechanisms remain incompletely understood; dominant routes of electron entry across the cell envelope under different conditions are unknown and there remains uncertainty surrounding the identity and regulation of key redox proteins in cathodic uptake. Additional uncertainties surround the rate limiting steps from the cathode surface to cytoplasmic ferredoxin/NAD­(P)H and the conditions under which direct electron transfer outcompetes mediated (H_2_/formate) pathways.

**Figure 15:**
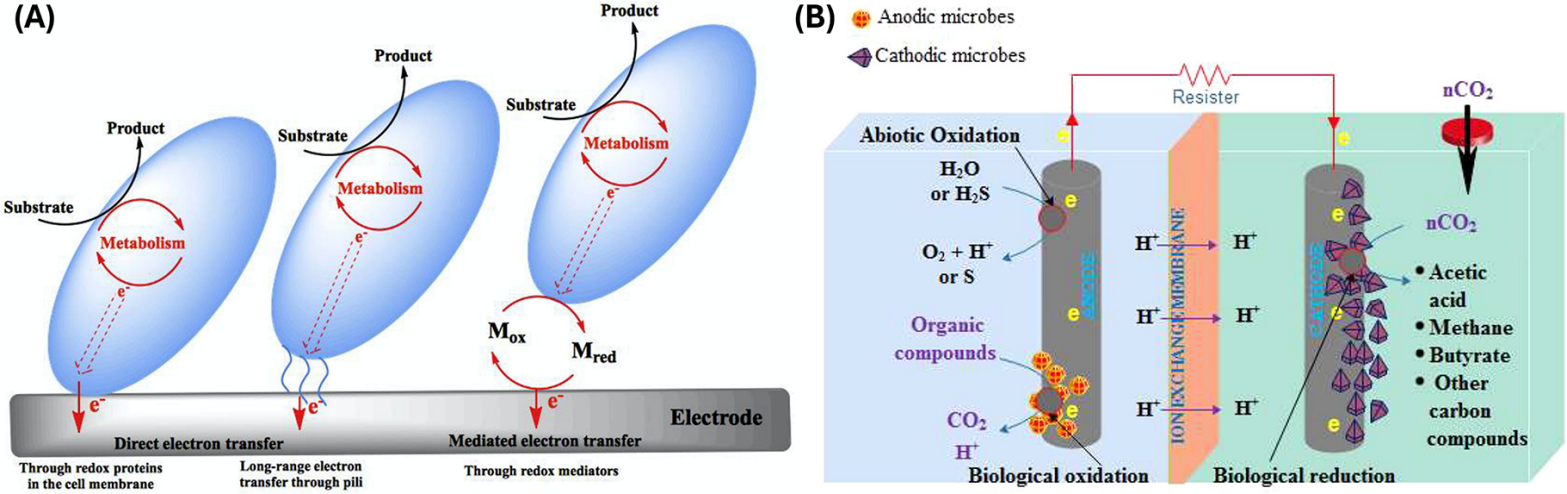
MES electron transfer mechanisms and potential product suite. (A) Mechanisms of electron transfer in microbial cells include (1) direct contact via cell membrane bound c-type cytochromes, (2) mediated electron transfer via extracellular redox-active electron shuttles (mediators), and (3) long-range electron transfer though pili in a bacterial microorganism. Reproduced from ref 190. Copyright 2020 American Chemical Society. (B) Schematic diagram of the microbial electrosynthesis carbon dioxide sequestration process and synthesis of value-added products. Reproduced from ref 191. Copyright 2023 American Chemical Society.

Additionally, MES systems often rely on complex microbial consortia; however, maintaining stable, selective, and productive communities over long time periods can prove challenging. Unwanted metabolic pathways can divert electrons and carbon flux toward competitive pathways, such as excessive abiotic H_2_ evolution, preferential biomass accumulation rather than product formation, and chain elongation to nontarget SCFAs/alcohols. The above challenges lead to poor production rates and low concentrations of target compounds (e.g., current densities ∼ 0.1–10 A/m^2^, with productivities often in the tens to hundreds of mg/L/day) relative to conventional sugar bioconversion pathways. Additionally, current electrode materials – such as graphite, modified surfaces with graphene, and carbon nanotubes – have high fabrication costs due to high surface area, biocompatibility, and corrosion resistance requirements. Gas–liquid mass transfer presents further hurdles to efficient nutrient and substrate delivery for MES deployment. Combined, these uncertainties currently cap achievable current densities and space-time yields, complicate scale-up and control of selectivity, and often force operation in regimes that favor H_2_/formate mediation rather than true direct EET.

Over the past five years, significant advancements have been made to address these challenges, including enhanced production rates of various chemicals (e.g., acetate), establishment of new microbial catalysts, and optimization of operational conditions (e.g., enhanced biofilm permeability and stabilization) to improve MES performance. For example, substantial improvement in acetate production rates and electron transfer have been achieved in recent years via biofilm modulation. Increasing the cell permeability of biofilms via enzymatic treatment enabled > 2X enhancement to electron transfer efficiency and acetate production.^192^ Similarly, biofilm stabilization into 3D-printed cathodes successfully enabled process intensification resulting in nearly 5X enhancement to acetate production in an algal-mediated MES system.^193^ Indeed, optimizing factors such as pH, temperature, and electrode materials has been crucial in enhancing MES efficiency. For example, studies have identified that neutral to slightly acidic pH levels (5.5–7.0) are optimal for CO_2_ conversion to acetic acid, while lower pH levels may be necessary to suppress unwanted methanogenesis.^194^ These enhancements are hypothesized to be mediated by a series of potential mechanisms including shortened electron transfer distances and improved contact density increase effective electron flux to cells; enhanced conductivity and redox active surfaces (graphene/composites) that facilitate charge transport through biofilms and better coupling to outer membrane cytochromes; and surface functionalization (e.g., polydopamine) promoting cell adhesion, biofilm stabilization, and/or chelation of metals that aid redox enzyme function. Biofilm permeability and optimized thickness can also reduce diffusion barriers for substrates/products, lowering internal mass transfer resistances and increasing overall reaction rates.

Advancements in novel electrode materials have also led to improved MES systems performance in recent years. Incorporation of carbonaceous materials into electrodes has the potential to increase the surface area and electrical conductivity thereof (please refer to Lekshmi, 2023 for extensive review^195^). For example, electrosynthetic formate production was recently achieved by photoassisted MES system by integrating a photoanode and an activated carbon fiber biocathode, enabling production of > 0.5 g/L formate.^196^ Additional advances in carbonaceous electrodes have been demonstrated via the utilization of graphene as a mediator, which has been shown to enhance cell growth and electron transfer rate in myriad microbial systems.^197^ For example, polydopamine-coated graphene aerogel anodes were recently demonstrated to achieve > 30% reduction in system start-up time and > 2X increases in maximum power density in *Geobacter*-mediated systems.^198^

Genetic modification of MES biocatalysts has also proved effective in enhancing system productivity (reviewed extensively in Klein, 2023 ^199^). Overexpression of nanowire proteins in *G. sulfurreducens* led to nearly 3-fold enhancement in in power density relative.^200^ Additional advances in *Geobacter* and *Shewanella* spp. systems have been achieved via isolation and development of novel microbial variants. For example, genetic engineering of *S. carassii*-D_5_ isolated from activated sludge achieved > 1.5X power densities relative to wild-type and > 5X higher than that achieved with the model electroactive microbe, *S. oneidensis* MR-1.^201^ The identification and deployment of new MES biocatalysts hold promise to both electrochemical parameters and expand the range of obtainable products.

While MES holds great long-term potential, outstanding challenges remain (please refer to Prevoteau, 2020 and Jourdin 2021 for extensive review), and the near-term promise of MES is likely in specific niches rather than bulk commodity production due to current limitations in rate, energy efficiency, electrode cost, and scale-up.^202,203^ Indeed, improving the efficiency of CO_2_ reduction, effectively scaling MES technologies, and achieving viable economics remain among the key hurdles facing this technology. Further, maintaining stable microbial communities over long periods can prove challenging, necessitating frequent “catalyst regeneration” cycles. Continued efforts to optimize operational parameters (e.g., temperature, pH, pressure), develop novel reactor and electrode designs, and identify novel microbes with enhanced native or engineered deployment characteristics, present numerous opportunities to continue to improve MES technology.

## 3 H_2_-Mediated CO_2_ Conversion

### 3.1 CO_2_ Hydrogenation

Thermocatalytic CO_2_ hydrogenation has been employed to generate hydrocarbon and oxygenate products. Hydrocarbon products of varying chain length have been targeted in single-reactor approaches, from the simplest, methane, through lower olefins (C_2_-C_3_), across the range of liquid fuel range products (C_5_-C_20+_), and to aromatics. In contrast to the range of C-C coupling chemistry observed for hydrocarbons, the most common oxygenate products retain the C_1_ fragment, methanol, dimethyl ether (DME, via dehydration of methanol), formic acid, and dimethyl carbonate (DMC). This section will summarize recent reports of high performing single-reactor approaches using tandem or multifunctional catalyst systems, highlight new catalyst materials where an understanding of how catalyst active site structure effects C-O bond breaking and C-C bond forming chemistry, and introduce a re-emerging target product, higher alcohol synthesis, that requires new catalysts to breakthrough traditional barriers. Notably, this section will not cover traditional reverse water-gas shift catalysis as an approach to generate carbon monoxide, since catalyst development and process considerations have been recently reviewed.^204–207^ However, approaches that initiate hydrocarbon production using RWGS will be highlighted. Similarly, standalone CO_2_ methanation and CO_2_ to methanol have been recently reviewed,^208–211^ including catalyst and process development aspects, and therefore, reports highlighted in this review with figures of merit to compare across emerging technologies will focus on alternative approaches to traditional processes (see Section 4). In line with the overarching theme for this review article, this section will focus on recent reports that build an understanding of the chemical mechanism for activating the CO_2_ molecule, rather than reviewing the chemistry and catalyst development to direct the reactive intermediate to hydrocarbon or oxygenate products.

Single-reactor approaches to C_2+_ hydrocarbon products via CO_2_ hydrogenation over tandem or multifunctional catalysts fall into two major categories based on the type of intermediate generated from CO_2_: (a) a CO intermediate followed by FT chemistry to light olefins (CO_2_-FT), and (b) a MeOH intermediate. Both the light olefins and the MeOH intermediates can be directed to a variety of hydrocarbon products, including fuel-range molecules and aromatics, typically through acid-catalyzed zeolite/zeotype carbon-pool chemistry. For historical context and advancements in this field made prior to 2020, which is before the scope of this review, the interested reader is directed to a comprehensive review of syngas and CO_2_ conversion to hydrocarbons.^212^ Since 2020, additional perspectives, review articles, and book chapters have also been published, focusing on specific catalyst combinations or products of interest.^213–219^

Of initial interest to the scope of this section is a summary of recent reports that have achieved high per-pass yield, high selectivity, or selectivity to a unique product slate using traditional FT catalyst metals for CO_2_-FT, and systems where these FT catalysts were combined with acidic catalysts to access higher hydrocarbons. Using traditional metals for FT catalysts, Fe and Co, a series of Na-modified bimetallic FeCo catalysts were explored by Li et al. for light olefin production from CO_2_.^220^ At 340 °C and 4.0 MPa, the optimized composition of Na_0.02_Fe_1_Co_1_ exhibited 56.9% CO_2_ conversion with just 3.6% CO selectivity, and 40.5% light olefins selectivity, giving a remarkable 22.2% light olefin yield.

An attractive alternative target product to light olefins (C_2–4_) is C_4+_ linear alpha-olefins (LAOs), which are valuable chemical precursors due to the terminal C = C bond. Xu et al. reported a ternary Fe-Zn-Al spinel catalyst based on an Fe FT catalyst that gave 88.7% LAOs among the C_4+_ products (53.5% selectivity to C_4+_) at 350 °C, 1.5 MPa, and 39.1% CO_2_ conversion.^221^ The ternary catalyst also exhibited 24.7% selectivity to C_2_-C_3_ olefins under these conditions, with a high overall olefin-to-paraffin ratio of 9.9. Modified Fe catalysts have also been studied for direct liquid fuels and aromatics production from CO_2_ hydrogenation. A Na and Cu modified Fe_2_O_3_ catalyst combined with hierarchical nanocrystalline HZSM-5 zeolite reached an aromatic selectivity of 57.7% at 33.3% CO_2_ conversion (320 °C, 3.0 MPa), highlighted further by 94.8% of the liquid product being aromatics.^222^ Building on a previous report demonstrating C_4+_ olefins from CO_2_ hydrogenation with a delafossite CuFeO_2_ catalyst,^223^ Cheng et al. coupled it with HZSM-5 to exhibit aromatics selectivity of 56.4% at high CO_2_ conversion (>50%) and with < 12% combined CO and CH_4_ selectivity.^224^

Finally, improvements in the production rate of liquid fuels from CO_2_ hydrogenation and subsequent acid catalysis has been the focus of recent work. Li et al. investigated the direct hydrogenation of CO_2_ to gasoline-range hydrocarbons and olefins over a series of bifunctional, K-modified FeMn catalysts paired with zeolites and operated at high temperatures, 300–400 °C.^225^ The optimized FeMnK+H-ZSM-5 catalyst provided 70% selectivity to an isoparaffin- and aromatic-rich C_5_-C_11_ hydrocarbon product at 320 °C at approximately 30% CO_2_ conversion. As expected, the hydrocarbon distribution was mainly a function of the micropore size of the zeolite, where the MFI structure gave the desired product slate for liquid fuels. Perhaps the most complex composition for a CO_2_-FT catalyst based on Fe, NaFeGaZr, was reported by Wang and co-workers.^226^ When combined with a hollow HZSM-5 zeolite and operated at 380 °C and 3.0 MPa, an aromatic-rich gasoline was produced with CO_2_ conversion near 45% for 200 h time-on-stream. An overarching theme for the development of these modified Fe-based FT catalysts is the use of an additional metal or metals that affects the formation of the active iron carbide phase, resulting in a composition that produces the light olefins more readily or shifts the selectivity to longer chain olefins, for subsequent conversion to the product of interest.

Similar to the CO_2_-FT approach, here we seek to summarize high selectivity and/or high yield to hydrocarbons from CO_2_ hydrogenation through MeOH using the traditional methanol synthesis catalyst, Cu/ZnO/Al_2_O_3_ “CZA”, and acid catalysts. It is worth noting that due to the mismatch in reaction temperature for MeOH synthesis using CZA (220–250 °C) and subsequent MTH chemistry (320 – 380 °C), many recent reports have utilized the Cu-free mixed oxide, ZnZrO_x_, to initiate methanol production at higher temperatures, and these reports have been recently reviewed.^211^ However, CZA still has its utility to be coupled with hydrocarbon production routes that are active below 300 °C. One report since 2020 stands out that builds upon initial reports that used 2-stage reactors,^227–229^ and extends a catalyst system that demonstrated CO_2_ coconversion with syngas.^230^ To et al. employed a stacked-bed composite catalyst of CZA with acidic alumina positioned above a Cu-modified BEA zeolite (Cu/BEA) for hydrocarbon synthesis in a single reactor. Relatively mild conditions of 210 °C and 3 MPa provided 13% CO_2_ conversion with high selectivity to C_4+_ hydrocarbons (96.4% within the hydrocarbon product, 35.3% overall) suitable for liquified petroleum gas (LPG) and gasoline fuels.^231^

The second topic of interest to this section is novel catalyst compositions for CO_2_ activation that are challenging the traditional wisdom by directing the chemistry through alternative mechanisms, leading to breakthrough performance for activity and/or selectivity. In CO_2_-FT, Co catalysts are a natural choice based on their known activity and selectivity for long carbon-chain products from analogous CO conversion. The particle size, oxidation state, crystal phase, and support are known to influence FT catalysis performance for Co catalysts,^232,233^ and interestingly, the rock salt phase of cobalt oxide on TiO_2_, CoO/TiO_2_, was identified to be more active than its metallic counterpart in 2014.^234^ In 2022, ten Have and co-workers uncovered the mechanistic aspects of CoO/TiO_2_ that differentiate it from metallic Co.^235^ The authors studied both CoO and metallic Co on reducible (TiO_2_, CeO_2_) and nonreducible (SiO_2_, Al_2_O_3_) supports. Only for the TiO_2_ support was CoO found to be more active than its metallic Co counterpart. Using sophisticated operando spectroscopy techniques, modulated excitation (ME) diffuse reflectance infrared Fourier transform spectroscopy (DRIFTS) with phase-sensitive detection (PSD), enabled the authors to differentiate the mechanism for CO_2_ activation. For metallic Co catalysts, the direct dissociation mechanism was followed, where CO_2_ dissociates to CO, which is the primary intermediate for either desorption or further dissociation to C_ads_ and subsequent chain-growth. On the contrary, CoO/TiO_2_ proceeded through the H-assisted mechanism, where CO_2_ forms carbonates, formates, or formyl intermediates followed by subsequent hydrogenation and chain-growth. Further, the authors investigated kinetic parameters, and an opposite relationship with H_2_ partial pressure was found, where CoO/TiO_2_ had a + 1.24 H_2_ reaction order and Co/TiO_2_ had a – 1.15 H_2_ reaction order. This reaction order analysis supports the H-assisted mechanism for the CoO/TiO_2_ catalyst. Finally, the authors used the mechanistic understanding to demonstrate that the CoO/TiO_2_ catalyst could outperform traditional metallic Co analogues and that H-assisted mechanism is a more favorable pathway to produce long-chain hydrocarbons than the direct dissociation mechanism. Feeding a mixture of CO and CO_2_, which may be expected in an industrial CO_2_ hydrogenation process that utilizes a recycle stream, a 2.8× increase in overall C_2+_ yield was achieved over the CoO/TiO_2_ catalyst compared to its reduced analog.

As presented above, Fe catalysts are similarly well-studied for CO_2_-FT to hydrocarbon products. Identifying that high CO selectivity was a limiting factor for Fe-based CO_2_-FT catalysts, Song and co-workers hypothesized that promotion of the catalyst with Cu could address this shortcoming. Targeting aromatics production, a composite catalyst of Cu-promoted Fe_2_O_3_ and HZSM-5 was developed, termed Cu-Fe_2_O_3_/HZSM-5. Meeting its intended goals, FT chemistry was enhanced over Cu-Fe_2_O_3_, decreasing CO selectivity to just 3.15% while achieving 56.61% selectivity to aromatics at high CO_2_ conversion of 57.30%. Through mechanistic studies, the authors identified a cooperative effect between the Cu-Fe_2_O_3_ catalyst component and the zeolite to effect the observed high selectivity to aromatics. They proposed a “H recycling” mechanism, where the dehydrogenation that must occur in the zeolite during aromatization generates “H species”, which could be H_2_ or could be a carbon-based reactive H-carrier. These H species can diffuse to the Cu-Fe_2_O_3_ surface and react with bound CO_2_. Thus, CO_2_ is both the overall reactant and a critical “H consumer” that enables the aromatization chemistry to proceed with high selectivity.

Taking the concept of Fe-oxide based catalysts to initiate CO_2_ conversion in a new direction, Tian and co-workers studied the perovskite, LaFeO_3_, as an alternative to traditional iron oxides paired with HZSM-5 zeolite for aromatics production.^236^ They hypothesized that uncontrolled C-C coupling on the Fe catalyst during FT chemistry complicated the subsequent aromatics chemistry occurring in the zeolite catalyst, limiting aromatics selectivity to < 70% within the hydrocarbon product slate. A critical design parameter for LaFeO_3_ over traditional Fe oxides was the resistance to carburization, where the Fe_5_C_2_ phase resulting from carburization of Fe oxides is a known active phase in FT chemistry for hydrocarbon production. Rather, the structure and energetics of LaFeO_3_ create a high barrier for carburization but still enable efficient CO_2_ hydrogenation. Indeed, when CO_2_ hydrogenation was conducted over the LaFeO_3_ catalyst alone (no zeolite), iron carbide was not formed and the catalyst exhibited little C-C coupling activity, giving 92.5% methane selectivity. Optimizing LaFeO_3_:HZSM-5 mass ratio (2:1) and Si/Al ratio (14) of the zeolite provided an extraordinary aromatics selectivity of nearly 85% at 61.4% CO_2_ conversion, and with CO selectivity of just 9.3%. Further, the catalyst system maintained high activity with extremely low deactivation rate over 1000 h time-on-stream. The mechanistic reasons for the enhanced performance was elucidated using in situ DRIFTS spectroscopy with cofed CO_2_ and H_2_ at 350 °C, where the LaFeO_3_ catalyst exhibited an oxygenate-rich surface comprised of formate, methoxy, and formyl species. In contrast, iron carbide catalysts displayed characteristic peaks for C = C and C-H species in their spectra under the same conditions. A computational assessment indicated that the mechanism through the formate species was more kinetically accessible than through bound CO*, supporting the alternative mechanism offered by the LaFeO_3_ compared to traditional Fe catalysts.

In line with our interest to highlight approaches that challenge the traditional wisdom, here we present reports that look beyond methanol as an intermediate from CO_2_ to fuels and chemicals, and rather, seek to develop catalysts that proceed through value-added higher alcohols (HAs), such as ethanol, propanol, and butanol. HA synthesis is a re-emerging field, where current researchers seek to breakthrough limitations identified in literature as far back as the 1980s that identified sulfide materials as active catalysts for syngas to HAs and a Mo-based catalyst for CO_2_ to HAs.^237^ Near the beginning of the time window of interest to this review, Xu et al. authored a comprehensive review article that outlined the thermodynamics of HA synthesis, the 4 main groups of catalysts being developed, effects of promoters and supports, and importantly, critical aspects of the mechanism that set stage for the past 5 years of research.^238^ Considering the thermodynamics, many CO_2_ hydrogenation reactions in the HA reaction network (RWGS, methanol synthesis, methanation, higher alcohols, higher alkanes) are exothermic, including alkane and alcohol synthesis, and notably excluding RWGS. Alkane products are more thermodynamically favored than alcohols from CO_2_ hydrogenation, setting the overarching HA catalysis challenge: it is truly a “catalyst problem”, necessitating the development of materials to control selectivity through a complex reaction network. Toward that goal, the 4 main categories of catalysts under development are modified MeOH synthesis catalysts (Cu-based), modified FT catalysts (Co, Fe based), noble metal catalysts (mostly Rh-based), and Mo-based catalysts (e.g., MoS_2_, Mo_2_C). Xu summarized the state of technology in 2020 as catalysts based on Cu > noble metals > Co for HA yield, and specifically noted an upper limit of HA selectivity of 35% as a limitation in traditional fixed-bed reactors. Across the types of catalysts, mechanistic considerations outline the challenge to balance the surface population of CO* and CH_x_* to balance chain-growth and hydrogenation to make C_2+_ alcohols instead of making methanol or alkanes. Constructing a catalyst necessitates structural control over (at least) two active sites for CO nondissociative (forming CO*) and dissociative adsorption coupled with balanced hydrogenation activity (forming *CH_x_, but not just CH_4_), and of course, the sites must be colocated (Figure 16). The role of promoters was somewhat unclear, and typically understood on a catalyst-by-catalyst basis, for example, the effect being different for Cu vs Co catalysts. Similarly, the opportunity to explore modulation of the intermediate binding strength, whether through promoters or strong metal–support interaction (SMSI) was identified.

**Figure 16:**
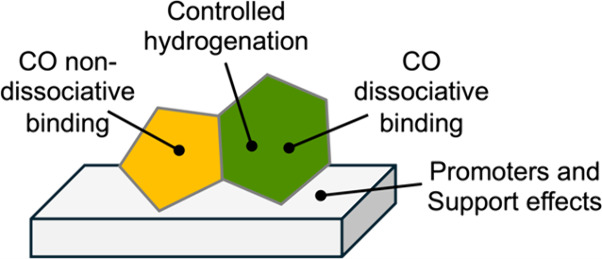
Schematic of the catalyst design challenge for higher alcohols synthesis.

In the 5 years since this review set the stage, the role of promoters remains linked to each type of catalyst, but a few reports provide an understanding of how specific elements act. For example, Na has a structural effect on Co catalysts, where the dispersion of the Co_2_C active phase increased with moderate Na loadings (up to 5 wt%).^239^ The Na sites also affected CO binding strength, and high Na loadings decreased CO binding energy, leading to high CO selectivity in the reaction. For a Rh catalyst, moderate loadings of Na also increased HA selectivity, and as expected, the promotion was deleterious at high Na loadings due to high surface coverage of Na.^240^ Na helped form Rh^+^ sites, which are important catalytic sites that work with Rh^0^ to affect the desired HA synthesis. Co-promotion with Na and Fe on a Rh/CeO_2_ catalyst was also found to be effective, especially for EtOH selectivity (reaching nearly 30%).^241^ Similar to Co_2_C, the promoters enhanced Rh dispersion and affected CO binding strength. For K on a multimetallic catalyst based on Cu and Fe (CuMgZnFe), again a moderate K loading (4.6 wt%) improved HA selectivity and catalyst activity.^242^ Here, the role of K was to achieve the desired balance of CO* and CH_x_* by controlling hydrogenation activity of the catalyst. For another CuFe-based catalyst, the role of Cr and K was investigated.^243^ Rather than attributing the modulated hydrogenation activity to K, the authors showed that Cr modification balanced hydrogenation activity and CO adsorption strength, decreasing CO dissociation and the associated Fe-carbide formation from activated C* species. These reports highlight that although detailed information about the role of promoters can be elucidated, the effects are often multifunctional and intertwined, and thus, design principles for each promoter element do not apply across all catalyst types of interest. Continued research to understand promoters and attempt to build unifying design rules across catalysts, or in contrast understand the different effects across catalyst types, would be a valuable area of research to advance this field.

A review by Tan et al. summarizes notable performance improvements over the past 5 years for the four types of catalysts noted above.^244^ Interestingly, despite recent reports for syngas to HAs over Mo-based catalysts, typically Mo_2_C, only one recent report studied CO_2_ hydrogenation over these promising catalysts.^245–251^ Ye et al. showed that Mo_2_C nanowires having exposed (101) surfaces were active for CO_2_ hydrogenation to methanol in a batch reactor at surprisingly low temperature of 150 °C using 1,4-dioxane solvent and a pressure of 6.0 MPa. The required dual active site for HA synthesis was prepared by depositing atomic Rh sites on the Mo_2_C nanowires, resulting in 44.6% selectivity to ethanol. Through detailed characterization of the Rh/Mo_2_C catalyst, the authors determined that additional modification with K was necessary to balance hydrogenation activity. Optimizing the reaction conditions for the RhK/Mo_2_C catalyst resulted in excellent ethanol selectivity of 72.1% at 150 °C, albeit with low ethanol yield (33.7 umol g^–1^ h^–1^) in the batch reaction.^252^ Given the emerging reports of more mild synthesis methods for Mo-carbide catalysts, including nanomaterials,^253,254^ these represent a promising scaffold to rationally construct the necessary active phases for HA synthesis from CO_2_ by depositing metals and promoters inspired by reports converting syngas.

### 3.2 Nonthermal Plasma CO_2_ Hydrogenation

In addition to direct CO_2_ splitting using NTP, indirect approaches for CO_2_ conversion with secondary reactants such as H_2_, CH_4_, C_2+_ hydrocarbons, H_2_O, etc. are advantageous in that they open the possibility of forming higher value products in a single step, which can help to mitigate the currently limited energy efficiency of common NTP approaches using DBD reactors.^125^ These types of reactions also frequently benefit from catalyst introduction to help guide the reaction pathways. Much of the work thus far has been focused on the synthesis of simple gas phase products such as syngas (CO + H_2_) and CH_4_, as well as low carbon number liquid products including acids, aldehydes, and alcohols, especially over thermochemically inspired catalysts based on transition metals on high surface area metal oxide supports.^124,255–257^

In addition to hydrogenation of CO_2_ via methanation (CO_2_ + 4H_2_ → CH_4_ + 2H_2_O) and reverse water gas shift (RWGS, CO_2_ + H_2_ → CO + H_2_O),^124,258–260^ direct methanol synthesis from CO_2_ hydrogenation stands out as a reaction of interest for NTP catalysis, which has been primarily studied in DBD reactors with catalysts due to the prevalence of syngas formation in warm plasmas.^124,261,262^ Although the reaction is favored at low temperature and high pressure, the kinetic limitation in CO_2_ activation at low temperature and high energy cost for high pressure operation provide an opportunity for NTP. In fact, in contrast to many multistep thermal processes, NTP approaches have demonstrated direct conversion of CO_2_ to methanol under ambient conditions.^263,264^ Many NTP investigations have focused on Cu-based catalysts predicated on those used for thermal reactions, seeking to understand the role of NTP generated species on the reaction pathways, specifically predicting a transition toward the formate route.^265^ Cui et al., have demonstrated a synergistic effect between a 4 wt% Cu/γ-Al_2_O_3_ catalyst and CO_2_/H_2_ plasma, leading to increased methanol selectivity particularly with H_2_O addition.^266^ The combined experimental and computational study highlighted the role of Eley–Rideal (E-R) mechanism in enabling the reaction to occur under atmospheric conditions.

Dry reforming of methane (DRM, CO_2_ + CH_4_ → 2CO + 2H_2_) and other reactions of CO_2_ with CH_4_, which are relevant for biogas (CO_2_ + CH_4_) conversion, for example, have been well studied by the NTP catalysis community,^256,267,268^ The formation of syngas is the most energy efficient in warm plasmas,^269,270^ with improvements arising from implementation of heat management strategies and postplasma catalyst integration,^271–273^ although catalysts can suffer from deactivation due to sintering and carbon deposition.^269,271–276^ Carbon particles have also been shown to impact the plasma characteristics when formed in the plasma region.^277,278^ Selective production of high value oxygenates with DBD plasma-catalyst combinations can overcome the limitations of energy efficiency, and multiple examples of synergistic effects between the NTP and catalyst have been reported^279,280^ giving rise to syngas, as well as other oxygenated hydrocarbons such as alcohols and aldehydes. These complex multireactant/product reactions have begun to benefit from mechanistic insight through both thoughtfully designed catalytic investigations, computational modeling, and in situ gas phase and catalyst surface spectroscopic characterization. A recent combined experimental and chemical kinetics modeling investigation by Qin, et al., provided insight into the gas and surface chemistry occurring in DRM over a Ni/SiO_2_ catalyst as the applied electric field increases to gas breakdown (and thus, plasma formation).^281^ In comparison to thermal catalysis at 500 °C, the application of external fields, both an electric field and NTP, increased the CH_4_ and CO_2_ conversion to syngas and hydrocarbons via the nonequilibrium excitation of the gas molecules. In the case of electric field catalysis, vibrationally excited CO_2_ facilitates dissociative adsorption, whereas in the NTP, reactive radicals, ions, and electronically excited species promote gas-phase and E-R reactions. Similarly, Sun et al., have shown through a combined experimental and computational investigation that both radicals and vibrational species present at the reduced electric field of 50–200 Td (Figure 17, left) accelerate dissociative desorption and E-R reactions in DBD DRM over a Ni/SiO_2_ catalyst.^279^ Furthermore, the E-R reactions enhanced by NTP generated H and O atoms consume carbon deposited on the catalyst surface by stepwise dehydrogenation of CH_4_ as shown in Figure 17 (right). Increased resistance of catalysts to coking during NTP catalysis has been noted as a beneficial feature of these processes.^282^ Similarly, reactions of CO_2_ with C_2+_ alkanes can yield an expanded product slate,^283^ and the addition of O_2_ has been shown to shift the products from syngas toward oxygenates due to the higher concentration of oxygen containing radicals such as O, OH, and HO_2_, from electron impact dissociation followed by reaction with H atoms.^284^

**Figure 17:**
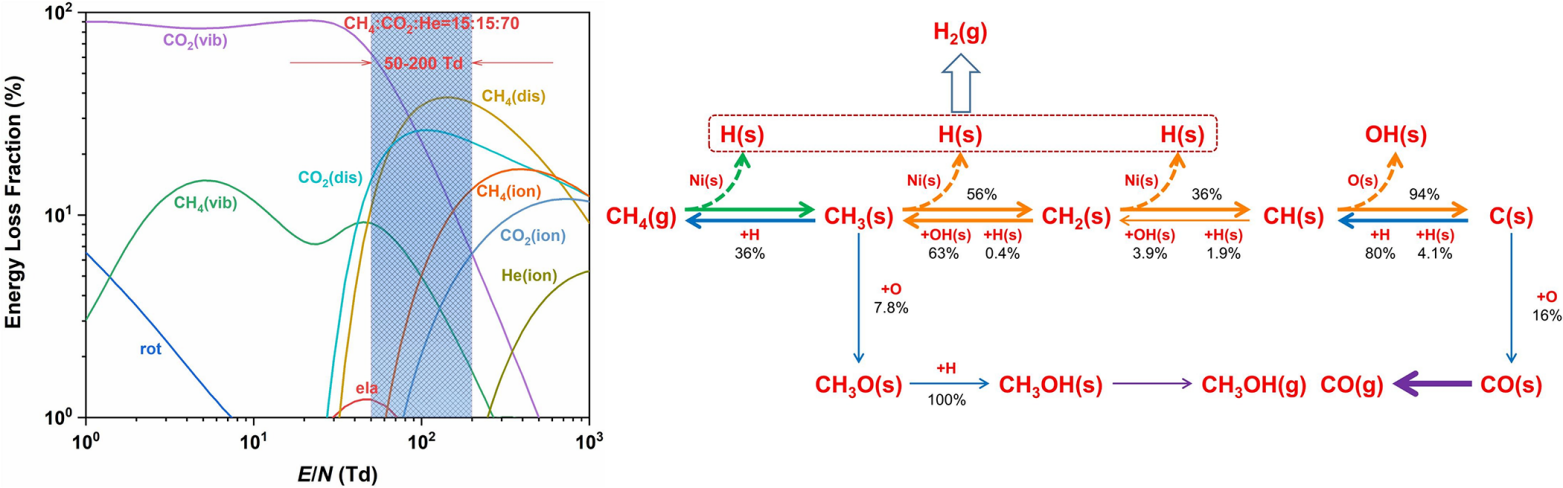
(Left) Fraction of plasma energy deposited into different excitation modes. (Right) Network of surface reaction pathways for carbon deposition C(s) and elimination for plasma-catalytic DRM over a Ni-based catalyst. Reproduced with permission.^279^ Copyright 2024 Elsevier.

Much of the NTP-catalytic CO_2_ hydrogenation literature, highlighted above, has focused the production of C_1_ molecules like CO, CH_4_, and CH_3_OH. Strategies to promote carbon coupling are of significant interest, though, especially given the target of higher value products for less energy efficient NTP systems. Successful thermocatalytic approaches to promote C–C coupling reactions such as adding water to the CO_2_ feedstock,^285^ have also been demonstrated in DBD-catalytic CO_2_ hydrogenation to form ethanol over a Cu_2_O/CeO_2_ catalyst with the assistance of water.^286^ Meng, et al. combined isotope-tracing experiments and DFT calculations to determine that H_2_O is dissociated in the NTP to produce adsorbed OH* and gas-phase OH radicals, both of which promote C–C coupling through CO-H_2_CO bonding and facilitate hydrogenation through proton transfer. Adsorbed H_2_O and OH enhances the desorption of ethanol, further increasing alcohol selectivity. While higher water concentrations favors selectivity to ethanol, lower concentrations (H_2_O/CO_2_ molar ratio below 0.5) facilitate methanol formation and higher CO_2_ conversion, consistent with previous work by this group and others,^266,287^ also highlighting the ability of H_2_O addition to act as a switch controlling alcohol selectivity.

Until this point, the NTP systems reviewed have operated in the gas and vapor phase, with water as a notable reactant. Liquid based conversion approaches, which, while not new, are being applied for a variety of CO_2_ conversion strategies to form valuable products. In gas–liquid systems, which are described in detail elsewhere,^130^ gas-phase generated plasmas can interact with the liquid, most commonly water, to create reactive species such as OH radicals, that can contribute to the formation of liquid products like methanol. In contrast to the commonly observed smaller hydrocarbon and oxygenate products, a recent contribution from Knezevic, et al., has demonstrated hydrocarbon chain growth up to C_40_ (including oxygenated products) from biogas constituents using nonthermal plasma in a bubbling gas–water system.^288^ This approach facilitates continuous removal of the long-chain products by phase separation of the solid products from water as shown in Figure 18 which likely also limits back reaction by the plasma. These solid products were only formed under conditions below 40% CO_2_ and not when 100% CH_4_ was delivered as the reactant, highlighting the role of CO_2_ chemistry in the process with the selectivity being highly dependent on the CO_2_/CH_4_ reactant ratio. Reaction pathways primarily mediated by radical intermediates were proposed for the (oxygenated) hydrocarbon and aromatic product formation (Figure 18). Although this work did not include a catalyst or other scavenging strategies, these approaches could be readily integrated to tune the selectivity of the products.

**Figure 18:**
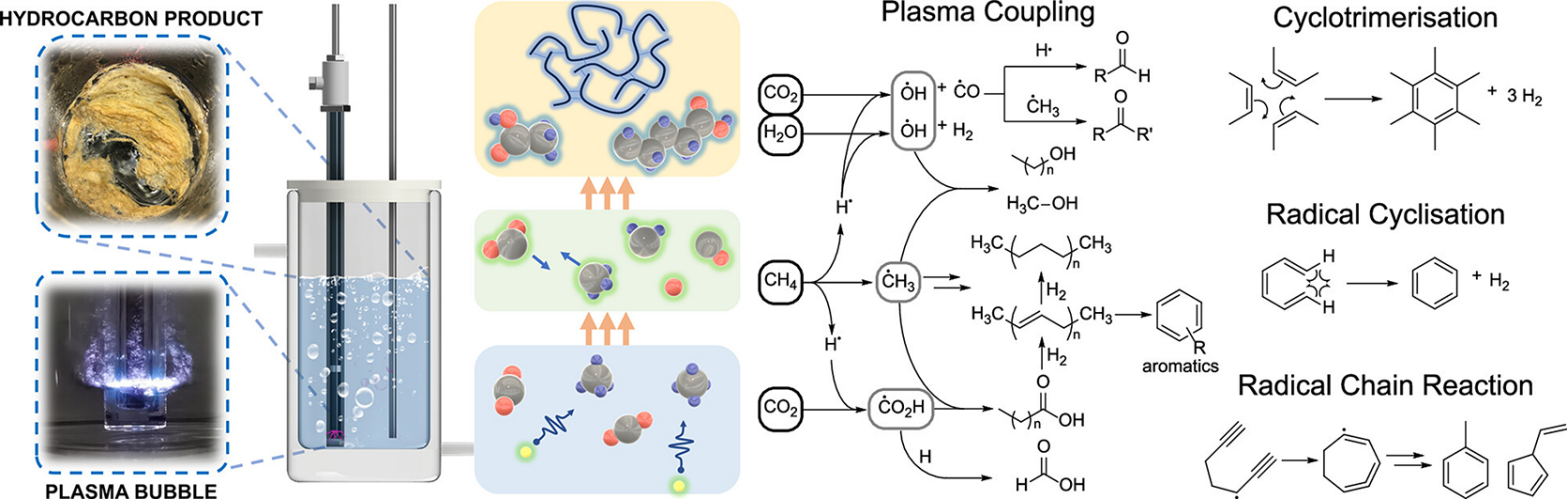
(Left) Schematic of the NTP reactor configuration, plasma bubbles, and solid long-chain hydrocarbon products. (Right) Proposed CO_2_/CH_4_ plasma coupling and aromatic formation pathways. Adapted from ref 288. Copyright 2024 American Chemical Society.

The majority of catalysts investigated for NTP CO_2_ conversion have been inspired by thermocatalytic systems. While these catalysts have provided notable insight into NTP catalytic chemistry, there exists a significant opportunity to tailor catalyst materials to the NTP environment.^135–137,289^ There are a variety of well-studied packing and/or catalyst systems such as ferroelectric materials that have been shown to impact the plasma properties and therefore the plasma chemistry (or chemical species in the plasma), and these and other catalyst features have been extensively discussed.^137,290^ To take advantage of the resulting plasma chemistry, several catalyst design criteria have been explored or proposed including (i) multivalent oxide catalysts that can accommodate oxygen vacancies for CO_2_ activation and/or to scavenge oxygen radicals, (ii) catalysts that can effectively react rather than quench radicals or vibrationally excited species, and (iii) photocatalysts to capture otherwise wasted energy from electronic transitions, although it is still challenging to deconvolute changes that the catalyst has on the plasma properties from the resulting chemistry. For a thorough discussion of these concepts, see a recent perspective from Bogaerts, et al. on plasma-catalyst synergy.^134^ Strategies have been explored both to promote targeted interactions between NTP generated species and catalysts or adsorbates on catalysts through E-R reactions and to selectively enhance the population of NTP activated species that contribute productively to catalytic reactions. As noted above, in DBD, the population of vibrationally excited CO_2_ is low, thereby limiting these types of reactions. Recently, Kim, et al., identified a 10 wt% Pd_2_Ga/SiO_2_ alloy catalyst that enhanced the proportion of vibrationally excited species in a fluidized bed DBD reactor, leading to CO_2_ conversion in the RWGS reaction exceeding the thermodynamic equilibrium.^291^

From a catalyst design perspective, photocatalysts have been found to improve the conversion efficiency in DBD and GA plasma by utilizing the ultraviolet–visible photon energy in the plasma emission spectrum, although there is still debate in the literature as to the impact the utilization of wasted photons can have on the efficiency of the plasma processes, especially for photocatalytic materials commonly integrated into plasma catalytic systems such as TiO_2_ with limited visible light absorption.^292^ Halide perovskites have more recently emerged as photocatalysts for CO_2_ reduction,^293^ due to high absorption coefficients, wide visible light absorption range, and tunable bandgap, spurring interest in this materials system for plasma catalytic applications. There are multiple examples of ABO_3_-type perovskite catalysts in the literature demonstrating enhanced NTP conversion of CO_2_, including heterojunction structures based on CsPbBr_3_ designed to promote separation and limit recombination of photoexcited carriers.^294,295^ Recently, machine learning was applied by Shen, et al., to develop an accurate predictive model for the DBD CO_2_ conversion efficiency for a Cs_2_TeCl_6_ photocatalyst based on experimentally tuned process parameters of catalyst mass, gas flow rate, and discharge power.^296^

Still, research is needed to deconvolute improvements in CO_2_ reactivity related to photoconversion (as well as plasma catalytic conversion in general) from changes to the plasma environment such as increased microdischarge time and density and improved charge uniformity, resulting from differences in the properties of the catalysts. In addition to NTP-photocatalytic systems, plasma technologies are finding their way into multiple hybrid technologies for conversion including electrocatalysis and biocatalysis, research areas that are also discussed in this contribution. These types of plasma hybrid technologies have been recently discussed for different catalytic conversion processes.^130,257,297^ Plasma technologies, particularly those combining multiple reactants/products, as a whole though, are hampered by low selectivity to higher value products beyond syngas for warm plasma systems and low energy efficiencies and selectivities for DBD plasmas. A greater understanding of reaction mechanisms is needed to help guide reactor and catalyst design to promote targeted reactions with high energy efficiency as well as to enable the effective utilization of abundant secondary reactants such as water and nitrogen that can expand the possible product slates.^298,299^

### 3.3 CO_2_ Fermentation

CO_2_ fermentation is a biotechnological process that converts carbon dioxide into value-added products using microorganisms or engineered enzymes (Figure 19). CO_2_ presents an abundant and relative cheap point source substrate. However, unlike sugar fermentation, in which the substrate carries the reducing power needed for product formation, CO_2_ bioconversion routes typically require external energy (e.g., H_2_, electricity, or light) to power product formation. As discussed above, falling renewable power costs and maturing green H_2_ supply chains have led to intensified interest in CO_2_ fermentation pathways. Indeed, recent advances in CO_2_ fermentation have enabled direct bioconversion to produce broad product suites, ranging from biopolymers to diverse fuel precursor molecules. This approach leverages the unique CO_2_-assimilating metabolisms of autotrophic microbes, such as acetogens, methanogens, and phototrophs, or synthetically engineered strains harboring new-to-nature CO_2_ fixation pathways, that utilize CO_2_ as a carbon source. The process is conventionally facilitated through canonical CO_2_ assimilatory pathways, including the Wood-Ljungdahl pathway, Calvin Benson Bassham (CBB) cycle, and the reductive tricarboxylic acid (rTCA) cycle (discussed in detail in Section 4.2).

**Figure 19:**
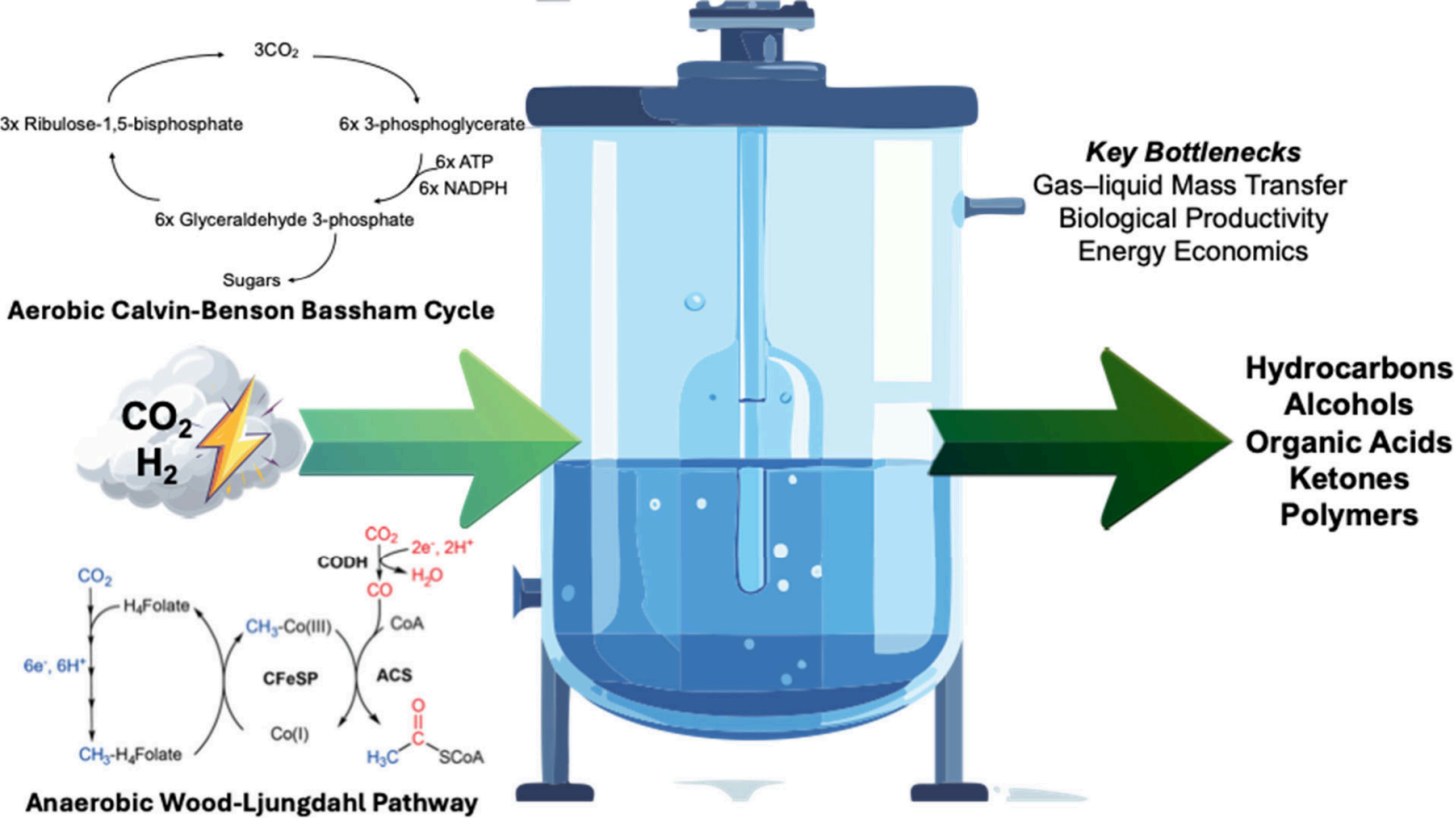
Overview of CO_2_ fermentation platforms for biological carbon conversion. CO_2_ is biologically converted to value-added chemicals and fuel precursors through microbial fermentation processes that couple carbon fixation with externally supplied reducing power. CO_2_ is provided as a gaseous substrate, while reductants such as H_2_, electricity, or light supply the electrons and energy required to drive endergonic reduction reactions. (Inset Left) Microbial carbon fixation is enabled by distinct metabolic pathways, including aerobic Calvin-Benson Bassham (CBB) cycle in phototrophic and chemoautotrophic organisms and the Wood Ljungdahl (WL) pathway in acetogenic bacteria, which channel CO2 in central metabolites such as acetyl-CoA and sugar phosphates. The WL methyl branch is colored blue and the carbonyl branch is colored red. CFeSP – corrinoid iron–sulfur protein, CODH – carbon monoxide dehydrogenase, ACS – acetyl-CoA synthase. WL pathway schematic reprinted from ref 300 (Right). Copyright 2022 American Chemical Society. These intermediates are subsequently converted into a broad product suite encompassing organic acids, alcohols, hydrocarbons, and specialty chemicals. Despite significant progress, key challenges remain, including low CO_2_ mass transfer, dependence on external energy inputs, limited product titers and rates, pathway and host constraints, and scale-up and process-integration barriers.

As of 2020, CO_2_ fermentation had reached pilot and early commercial stages, with ongoing research focused on improving CO_2_ assimilation and biosynthetic metabolic pathways, optimizing reactors for enhanced gas mass transfer, and reducing costs via enhanced process efficiencies to enable large-scale industrial deployment. Indeed, companies like LanzaTech, NovoNutrients, and Solar Foods have begun commercializing CO_2_-based fermentation for biofuels, single-cell proteins, and specialty chemicals. Additionally, integration with carbon capture and utilization (CCU) initiatives was gaining traction, positioning CO_2_ fermentation as a viable solution for reducing industrial emissions. However, despite the technological potential of CO_2_ fermentation, viable deployment continues to face several technical, economic, and biological challenges. CO_2_ is notably a highly oxidized and thermodynamically stable molecule, with relatively low energy content, thus requiring significant energy input to be converted into reduced carbon compounds. This, in turn, necessitates efficient energy sources, such as hydrogen, electricity, or light to help drive assimilation, which can be costly or technically challenging to integrate. Indeed, autotrophic microorganisms often require external reducing agents (e.g., H_2_, formate, or CO) to drive CO_2_ conversion. Ensuring a sustainable and cost-effective supply of these electron donors is crucial for scalability (discussed further in Section 6).

Related, many CO_2_-fixing microorganisms have slow growth rates (e.g., > 3hr doubling time for model acetogens versus ∼ 20–30min for *E. coli*) and 2–3× lower volumetric productivities compared to sugar-utilizing heterotrophic systems. This is due, in part, to mass transfer induced limitations to substrate availability and decreased flux to reduced products relative to conventional sugar bioconversion platforms. Further, while metabolic engineering and synthetic biology approaches have enabled the development of improved CO_2_-fixing strains (Section 4.2), many autotrophic microbes have complex regulatory networks and are less amenable to genetic modifications than model organisms like *E. coli* or *Saccharomyces cerevisiae*. Finally, from a deployment perspective, CO_2_ has low solubility in aqueous media (poor gas–liquid mass transfer), which restricts its bioavailability for microbial uptake. Reactor design improvements, such as gas fermentation systems with enhanced gas solubilization and mass transfer capacity, are needed to overcome this limitation. Addressing these challenges requires interdisciplinary efforts in metabolic engineering, reactor design, process optimization, and policy support to make CO_2_ fermentation a viable strategy for sustainable bioproduction.

A key advance in recent CO_2_ fermentation efforts is the utilization of hydrogen as an energy and/or reductant source and is the basis for several emerging “power-to-x” technologies. Hydrogenotrophic microorganisms have been engineered to convert CO_2_ and H_2_ into fuel molecules, such as ethanol or butanol.^301^ Indeed, *Clostridium autoethanogenum* is an exemplary Clostridia that has recently been metabolically rewired to produce diols, ethylene glycol, and ethyl acetate via targeted metabolic engineering and incorporation of heterologous biosynthetic components, among other high-value metabolic intermediates.^302–305^ By tuning the ratio of CO_2_:hydrogen in these microorganisms’ feed regimes, metabolism can be shifted toward higher yields of desired target products.^304,306^ Metabolic engineering efforts in *Rhodobacter* spp. have enabled photofermentative production of hydrogen, polyhydroxybutyrate, and terpenes from CO_2_.^307–310^ Researchers have also engineered variants of the facultative chemolithotroph, *Cupriavidus necator*, to produce myriad product suites, including methyl ketones, polyhydroxyalkanoates, and isoprenoids (for extensive review, please refer to Panich, 2021 ^311^). Additionally, this organism has been proposed as a chassis for the production of microbial protein from CO_2_ and diverse waste substrates.^312^ There have also been substantial advances in CO_2_/H_2_ fermentation using methanogenic archaea for the production of methane (CH_4_), wherein CO_2_:H_2_ tuning has been shown to impact both archael community structure as well as production of CH_4_.^313,314^

Given the poor solubility of CO_2_ and H_2_ in water, a key hurdle in efficient CO_2_ fermentation strategies is the optimization of gas–liquid mass transfer. Alternative reactor configurations, such as bubble columns and rotating packed bed (RPB) reactors, present opportunities for enhanced mass transfer.^315,316^ Biocatalyst immobilization offer additional opportunities to reduce mass transfer barriers while achieving improved process intensification.^317,318^ Recent studies have employed inverse modeling to define bubble size dynamics, leading to improved predictions of gas holdup and mass transfer rates, and in turn, more efficient reactor designs.^319^ Optimization of design and operating conditions of RPB-based CO_2_ capture systems have also been evaluated, demonstrating cost savings and reduced packing volumes compared to traditional methods, and highlight the potential of RPBs in scaling up CO_2_ fermentation processes.^320^ Experiments using dielectric barrier discharge (DBD) reactors in burst mode have notably shown increased CO_2_ conversion rates and energy efficiency in preliminary studies.^321^ Finally, recent analyses have evaluated gas mass transfer enhancements in external loop reactors, reflective of proprietary configurations deployment in industry (e.g., Lanzatech’s syngas conversion technologies).^322^ Computational fluid dynamics defined the impact of bubble size and product formation (ethanol) upon volumetric mass transfer coefficient and mass transfer capacity, suggesting mass transfer limitations can be alleviated for some gas bioconversion processes. Collectively, recent advances in reactor design portend substantial advances to the field of gas fermentation at large, including specific benefits to poor solubility gaseous substrates, such as CO_2_.

## 4 Emerging Trends in CO_2_ Conversion

### 4.1 Reacting Bound States of CO_2_ via Reactive Carbon Capture (RCC)

An integrated CO_2_ capture and utilization approach known as reactive carbon capture (RCC) has recently emerged as a process to access reduced, value-added products through a process intensification approach that first adsorbs CO_2_ and then reacts the bound species, typically by switching the feed to H_2_, in a single reactor. This process is especially attractive for dilute CO_2_ streams, such as flue gas streams having 5–15% CO_2_ where the capture costs are comparatively higher than concentrated CO_2_ streams (e.g., ethanol fermentation off-gas at 99%).^323–325^ The advantages arise from the ability to circumvent multiple process steps and associated capital expenditures of the typical approach, capture – release – purification – compression – transport – storage, prior to the desired utilization. Considering advantages from the chemistry perspective, adsorption of the CO_2_ molecule at a capture site breaks the linearity and thereby activates the bound carbonate or bicarbonate species for reduction at lower temperatures and pressures. By keeping the bound CO_2_ in the reactor for the reductive desorption step, the RCC approach opens new routes to control selectivity through catalyst design compared to traditional cofed CO_2_ hydrogenation reactions. From the process perspective, the ability to work with dilute CO_2_ streams, including direct air capture, enables modularity in process design to match the scale of CO_2_ source, H_2_ source and product volumes required at the site, going against traditional wisdom to take advantage of economies of scale by always seeking large-scale equipment that may not match all three considerations noted here. Considering the case of methanol production via this approach, Freyman and co-workers demonstrated that there is potential to reduce cost and energy requirements by nearly 50% each compared to a conventional capture and conversion approach, clearly motivating research to realize these savings.^326^

Early reports of RCC focused on methanation in an isothermal reactor, where CO_2_ capture was followed by H_2_ reduction at a single temperature (320 °C) and atmospheric pressure.^327,328^ This group published a series of papers outlining the necessary components of dual function materials (DFMs, also called bifunctional materials, BFM), where adsorbent species, such as CaO and Na_2_O, are coupled with traditional methanation catalysts based on Ru and Ni.^329–333^ Initial reports highlighted the applicability to flue gas streams, and later, the materials were shown to perform well under conditions relevant to direct air capture and conversion (i.e., 420 ppm CO_2_).^334^ These early reports have been recently reviewed,^335^ including a review of the industrial applicability of DFMs.^336^ It is important to note that many fundamental catalysis science aspects remain unexplored for these DFMs, especially the interplay and interface between capture sites (alkali metal oxides) and hydrogenation sites (transition metals). The role of moisture and impurities on CO_2_ binding and reaction mechanisms is an ongoing area of research by this group,^337^ and additional spectroscopic and mechanistic studies would be a welcome addition to this literature. Notably, these early reports utilized alkali and alkaline earth metals as capture agents that can operate at the temperatures needed for methanation (>300 °C). Conceptually, researchers have targeted tethered amines as capture agents due to their utility for CO_2_ capture from air and industrial sources. However, a recent paper presents two case studies that identify the mismatch between capture temperature (<80 °C) and reactive desorption temperature (>200 °C) as a limiting factor for amine-based DFMs, in addition to potential catalyst site poisoning by the amine.^338^ Thermal degradation of amine capture agents at the elevated temperatures required for catalysis further constrains the practical application of amine-based reactive capture systems. Building on the demonstration of an amine-based photoswing direct air capture system,^339^ which established light-driven regeneration as a viable alternative to thermal desorption, Halingstad et al. recently reported a photoreactive capture system that directly couples CO_2_ capture with catalytic conversion.^340^ The design employed broadband-absorbing titanium nitride (TiN) nanoparticles dispersed within a polymeric amine matrix to enable CO_2_ hydrogenation under comparatively mild conditions, achieving conversion of ∼ 70% of captured CO_2_ to CH_4_ across multiple cycles without measurable amine degradation.

Crawford et al. extended the DFM concept to be built upon microporous zeolites, using faujasite (FAU) as the support for Na-based CO_2_ capture sites, termed zeolite dual-function materials, ZFMs.^341^ The construction of Ru-Na/FAU directed the captured CO_2_ to methane and Pt-Na/FAU generated CO, demonstrating selectivity control through the addition of the appropriate catalytic metal. This report utilized a temperature swing for the reactive desorption step, rather than the isothermal approach pioneered by Farrauto, and high yields of 80–100% of the captured CO_2_ were observed. Rather than using different DFM compositions to direct selectivity, reports from the past 5 years have presented the concept of “switchable” catalysis, where the same DFM composition could target methane or CO by the choice of reaction conditions. The concept was first reported in traditional, fixed-bed CO_2_ hydrogenation feeding a 4:1 mixture of H_2_:CO_2_ over a series of Fe- and Ru-promoted Ni/Ce_0.5_Zr_0.5_O_2_ catalysts. The Ru-Ni catalyst demonstrated high selectivity (>80%) for methane at 350 °C and CO at 700 °C, and could be cycled back-and-forth between these two temperatures day-by-day.^342^ Merkouri et al. extended the concept to cyclic RCC operation with either H_2_ or CH_4_ fed in the reactive desorption step to generate CH_4_ via methanation with H_2_, or CO via RWGS or dry reforming of methane (DRM). Here, the more traditional DFM composition employing an alkali CO_2_ capture agent (Na, K, Ca oxides) with a Ni-Ru bimetallic catalyst were supported on a CeO_2_-Al_2_O_3_ support.^343^ The authors identified the NiRuCa formulation as the overall best DFM, providing per-cycle yields of 104 μmol_CH4_/g_DFM_ in methanation, 58 μmol_CO_/g_DFM_ in RWGS and 338 μmol_CO_/g_DFM_ in DRM, which are comparable to the molar yields from methanation reported by Farrauto. Merkouri et al. also performed TEA for a conceptual switchable catalysis process.^344^ The authors reported that methanation was more profitable than targeting methanol synthesis via CO, and a low payback period of just 4 years was determined. As with many CO_2_ hydrogenation approaches, whether traditional fixed-bed cofed hydrogenation or RCC, the cost of electricity and H_2_ are key economic drivers.

More recently, methanol has been targeted as a value-added product directly from CO_2_ via RCC approaches, that is, not through CO generation via RCC with subsequent methanol synthesis. The opportunity was well-motivated through an analysis of a conceptual RCC process, where significant savings in capital expenditures, operating expenses, and total energy input were identified for RCC versus the multistep CO_2_ capture and utilization approaches.^326^ A critical assumption was catalytic performance from the DFM that resembled typical methanol synthesis, presenting the experimental targets that needed to be achieved to realize the benefits. Experimentally, two notable recent papers have made progress toward these cost and energy savings. Using a stacked-bed approach, rather than a DFM, Wirner et al., demonstrated 150 μmol/g capture on a Na/Al_2_O_3_ sorbent sitting above traditional CZA methanol synthesis catalyst, but only 12 μmol_MeOH_/g yield due to low conversion of the captured CO_2_ (20%) with low selectivity to methanol (22%). Jeong-Potter et al. used similar components, but added the alkali capture sites (K, Ca) directly to the CZA catalyst, giving DFM compositions termed Alk/CZA.^345^ K/CZA provided the best performance of this series in RCC cycles, giving 59.0 μmol_MeOH_/g_DFM_ (46% selectivity from captured CO_2_) in a temperature–pressure swing cycle. Importantly, the methane selectivity was just 4.4%. The authors presented important catalysis science concepts to consider for temperature and/or pressure swing operation, such as how the changing ratio of H_2_/CO_2_ and surface populations must be considered to develop accurate mechanistic explanations that can lead to new DFM design. This group also explored the TEA and LCA around the Alk/CZA catalyzed RCC-to-MeOH approach, bringing the experimental data into a similar contextual framework outlined by Freyman.^346^ Using the moderate performance from the CZA parent material reported by Jeong-Potter et al., not the best-performing K/CZA, the TEA identified a path toward cost-competitive MeOH production compared to the multistep CCU approach. As noted above, green H_2_ cost was a key driver. Interestingly, the LCA identified a 21% water consumption savings using the RCC approach over traditional CCU. This was attributed to significantly less water required in RCC for the CO_2_ capture step, which requires steam regeneration in the baseline CCU process used as a comparison.

The above thermal approaches have demonstrated success to a variety of products. Nonetheless, limited throughput caused by deadtime associated with startup, shutdown, and heating/cooling between cycle steps, as well as low efficiency due to desorption of unreacted CO_2_ during heating in some cases is a significant challenge for the advancement of RCC processes. Utilization of NTP to activate bound as well as desorbed CO_2_ has the potential to ease the transition between the capture and conversion steps leading to improvements to overall process efficiency.^151^ Similar NTP-based approaches to activate and convert CO_2_ discussed in Section 2.3 can be applied for NTP RCC with inclusion of a solid sorbent material that is first saturated with CO_2_ from various feedstock streams.^347–349^ Liquid sorbents including ionic liquids (ILs), have been extensively studied for CO_2_ capture and it has recently been demonstrated that the captured CO_2_ can be converted to CO using plasma.^350^ The differences in solubility between CO_2_ and CO in the IL solution may also provide an avenue for product recovery.^351^

As with noncapture processes, other gases, specifically hydrogen sources, can be introduced during the desorption/reaction step.^352^ Li, et al., investigated dry reforming of methane (DRM) in a DBD plasma reactor over the sorbent zeolite 5A. Methane addition during plasma-induced desorption of preadsorbed CO_2_ yielded products including H_2_, CO, hydrocarbons, and the byproduct H_2_O. Although the majority of the observed products with the exception of CO can arise from CH_4_ nonoxidative coupling, the presence of H_2_O in the product stream indicated possible DRM reactions.^353^ All the examples described thus far focus on a sorbent only configuration rather than integration with a DFM, which can provide catalytic reactivity. While many DFMs are suitable only for adsorption of CO_2_, metal organic frameworks (MOFs) such as the Zn-based MOF-177, investigated by Gorky, et al., for RCC in a DBD reactor, can adsorb both CO_2_ and CH_4_, a challenging prospect due to the nonpolarity of CH_4_ that only weakly interacts with most materials.^354^

Outstanding questions in the utilization of NTPs to activate bound CO_2_ following capture from feed streams include mechanistic insights regarding surface versus gas phase reactivity, the role of DFMs in these processes, the impact of heating on desorption/reaction, and the influence of contaminants (e.g., H_2_O, O_2_ from DAC of CO_2_) representative of realistic application of NTP RCC technologies. In summary, RCC is an emerging approach for CO_2_ hydrogenation to fuels and chemicals that offers potential advantages over traditional, large-scale, multistep capture and conversion approaches. A variety of C_1_ products can be accessed, and future work should focus on catalysis science to develop structure-performance relationships that can inform next-generation materials that can breakthrough low per-cycle yields to C_1_ products and potentially enable selectivity to C_2+_ products.

### 4.2 Synthetic CO_2_ Assimilation Pathways

As discussed in Section 3.3., biological upgrading of CO_2_ presents a promising platform for direct conversion of CO_2_ sources but is limited, in part, by energy-intensive and relatively slow kinetic CO_2_ assimilation pathways. Indeed, an array of autotrophic microbes – possessing native metabolic machinery for capture and conversion of CO_2_ – have been developed as biological conversion platforms to produce myriad bioproducts.^303,304,307,311,355–357^ The primary CO_2_ fixation pathways include the CBB cycle, the Wood-Ljungdahl (WL) pathway, the reductive tricarboxylic acid (rTCA) cycle, the 3-hydroxypropionate (3-HP) bicycle, and the archael hydroxypropionate-hydroxybutylate (HP-HB) cycle. Each pathway is characterized by distinct enzymatic mechanisms and energy requirements, with some relying on ATP-driven carboxylation reactions with varying energy requirements and reductant generation potential (Table 2). Such native biological CO_2_ fixation pathways are inherently inefficient, and present large energy demand in the form of ATP, often limiting their deployment potential (for comprehensive review of CO_2_ fixation pathway thermodynamics, please refer to (Zhao, 2021)^358^). Indeed, native CO_2_ fixation pathways face both energy and reducing power constraints, requiring substantial ATP input or reduced cofactors (e.g., NAD­(P)­H, ferredoxin), limiting their efficiency in non-native hosts (Table 2).

**Table 2:** Comparison of Natural and Representative Synthetic* CO_2_ Fixation Pathways^a^

Pathway		No. of Reactions	Product	ATP/CO2 (mol/mol)	NAD(P)H/CO2 (mol/mol)
Calvin cycle	aerobic	11	GA-3P	3	2
3-hydroxypropionate bicycle	aerobic	16	pyruvate	1.67	1.67
Wood–Ljungdahl pathway	anaerobic	8	acetyl-CoA	0.5	2
reductive TCA cycle	anaerobic	9	acetyl-CoA	1	2
dicarboxylate-4–hydroxybutyrate cycle	anaerobic	14	acetyl-CoA	1.5	2
3-hydroxypropionate–4-hydroxybutyrate cycle	aerobic	16	acetyl-CoA	2	2
*CETCH cycle*	*aerobic*	*12*	*glyoxylate*	*1*	*1*
*ASAP*	*aerobic*	*11*	*starch*	*0.5*	*2*
*POAP cycle*	*anaerobic*	*4*	*oxalate*	*1*	*0.5*

a Synthetic pathways are denoted in italic. Reprinted from ref 359. Copyright 2022 American Chemical Society.

Additionally, enzymatic and carbon conversion efficiency as well as oxygen sensitivity hinder key assimilation enzymes. For example, the key assimilatory enzymes ribulose-1,5-bisphosphate carboxylase/oxygenase (RuBisCO) in the CBB cycle and carbon monoxide dehydrogenase (CODH) in the WL pathway, respectively, often suffer from low catalytic efficiency and/or oxygen sensitivity, reducing overall carbon fixation rates.^360,361^ Notably, some CO_2_ fixation pathways, such as the rTCA cycle, require precise regulation to balance intermediate metabolite levels, as excessive accumulation can lead to toxicity.^362^ Indeed, introduction of autotrophic CO_2_ fixation pathways into heterotrophic microorganisms often requires the optimization of cofactor regeneration, redox balance, and central metabolism to support autotrophic growth.^363^ Finally, while CO_2_ fixation pathways have been extensively studied in laboratory conditions, their large-scale implementation in biotechnological applications requires further optimization of enzyme kinetics, thermodynamics, and metabolic flux.

Recently emerging synthetic biology efforts have targeted the development of new-to-nature CO_2_ fixation pathways with enhanced energetics and efficiency to overcome the hurdles associated with conventional CO_2_ assimilation pathways. The first reported synthetic CO_2_ fixation pathway entailed the retrosynthetic development of the crotonyl–coenzyme A (CoA)/ethylmalonyl-CoA/hydroxybutyryl-CoA (CETCH) cycle (REF).^364^ The CETCH cycle is comprised of 17 enzymes derived from nine different organisms spanning all domains of life. Following extensive optimization, the cycle was successfully deployed in vitro, demonstrating nearly 5X higher efficiency and requiring substantially fewer photons per CO_2_ molecule fixed than conventional photoautotrophic pathways. To catalyze the reductive carboxylation of enoyl-CoA esters, the cycle leverages enoyl-CoA reductases (ECR), which are notably absent from all native CO_2_ fixation pathways reported to date. ECR enzymes present promising catalysts as components of synthetic CO_2_ fixation due, in part, to their broad substrate spectrum, oxygen insensitivity, and requirement of NADPH as a sole cofactor. Additionally, as recently noted by Tommasi,^365^ ECR catalytic efficiency is approximately 2× greater than that of RuBisCO.

In the past five years, additional synthetic fixation pathways have been reported, achieving even higher conversion rates and efficiencies, underscoring the promise of synbio-mediated reactive capture strategies. For example, Cai and colleagues developed a hybrid chemo-biochemical pathway for starch synthesis from CO_2_ and hydrogen in a cell-free enzyme-based system. The artificial starch anabolic pathway (ASAP), consisting of 11 reactions, demonstrates nearly order-of-magnitude higher CO_2_-to-starch biosynthetic rate than that observed in maize and 5-fold higher rate than the CETCH cycle.^366^ Another promising example is the tartronyl-CoA (TaCo) pathway, which facilitates direct assimilation of glycolate into central carbon metabolism.^367^ Notably, this pathway enables carbon-positive photorespiration via the development of a new-to-nature enzyme, glycolyl-CoA carboxylase (GCC). The pathway notably facilitates carbon fixation that is independent of RuBisCO oxygen sensitivity limitations, bypassing a key bottleneck in conventional photosynthetic CO_2_ fixation. Finally, in 2025, Santanowski and colleagues introduced the CORE cycle, a synthetic metabolic pathway that converts CO_2_ to formate at aerobic conditions and ambient CO_2_ levels, using NADPH as a sole reductant source, representing yet another advance in the field.^368^ Synthetic CO_2_ fixation pathways continue to emerge with continually improving energetics and efficiency; development of such new-to-nature CO_2_ fixation pathways underscores the power of synthetic biology, wherein heterologous and native metabolic machinery can be paired to establish new-to-nature functionalities. Effective incorporation and optimization into production hosts will be needed to bring to bear the full potential of these pathways.

## 5 Cross-Comparison of CO_2_ Conversion Pathways

With CO_2_ conversion R&D experiencing exponential growth over the past decade, a wide assortment of potential conversion technologies have received attention, spanning technology readiness levels (TRL) from laboratory to commercial scale as discussed in Sections 2 – [Sec sec4]. A natural question that arises from such a diverse set of potential conversion pathways is “under what conditions might a future user seek out one pathway over another?” or “what are the purported advantages or unique aspects of one pathway versus another?”. In Section 5 we offer perspectives around these questions and provide a high-level comparative summary of the seven noted conversion pathways to highlight key cross-cutting technical metrics and their relative advantages and limitations. The intention of this assessment is not to endorse any one technology but rather emphasize key defining attributes of each of the noted conversion pathways along with the potential opportunity space.

The pathway summaries shown below are split across two comprising tables. The first table examines the technical cross-cutting metrics of pathways while the second table highlights qualitative characteristics of each pathway and summarizes commonly cited strengths, challenges, and tradeoffs. In both tables the pathways are loosely ordered by technical maturity with the lowest TRL appearing on the left and progressively moving right to higher TRL.

The cross-cutting technical metrics reported in Figure 20, while offering a snapshot of current performance ranges reflective of recent literature, should not be considered without an understanding of their key caveats and shortcomings which are summarized here. One notable limitation in this approach involves pathway product distribution. Specifically, no one single product is consistently produced across all seven pathways, making an apples-to-apples comparison of technical performance impractical. In response to this limitation, our approach has been to, where appropriate, report a range of published values for each metric across the most commonly cited C_1_-C_2_ products over the last 5 years. For example, the NTP, LTE, and HTE pathways may reflect the performance of synthesizing carbon monoxide whereas the MES and BC data may be more reflective of acetate and methane formation, owing to their specialized biological assimilation chemistry. Our aim is that this approach provides an “order of magnitude” idea of what to expect for a given pathway, which, in reality, varies based on process complexity, targeted end product, and the specific operating conditions of the process.

**Figure 20:**
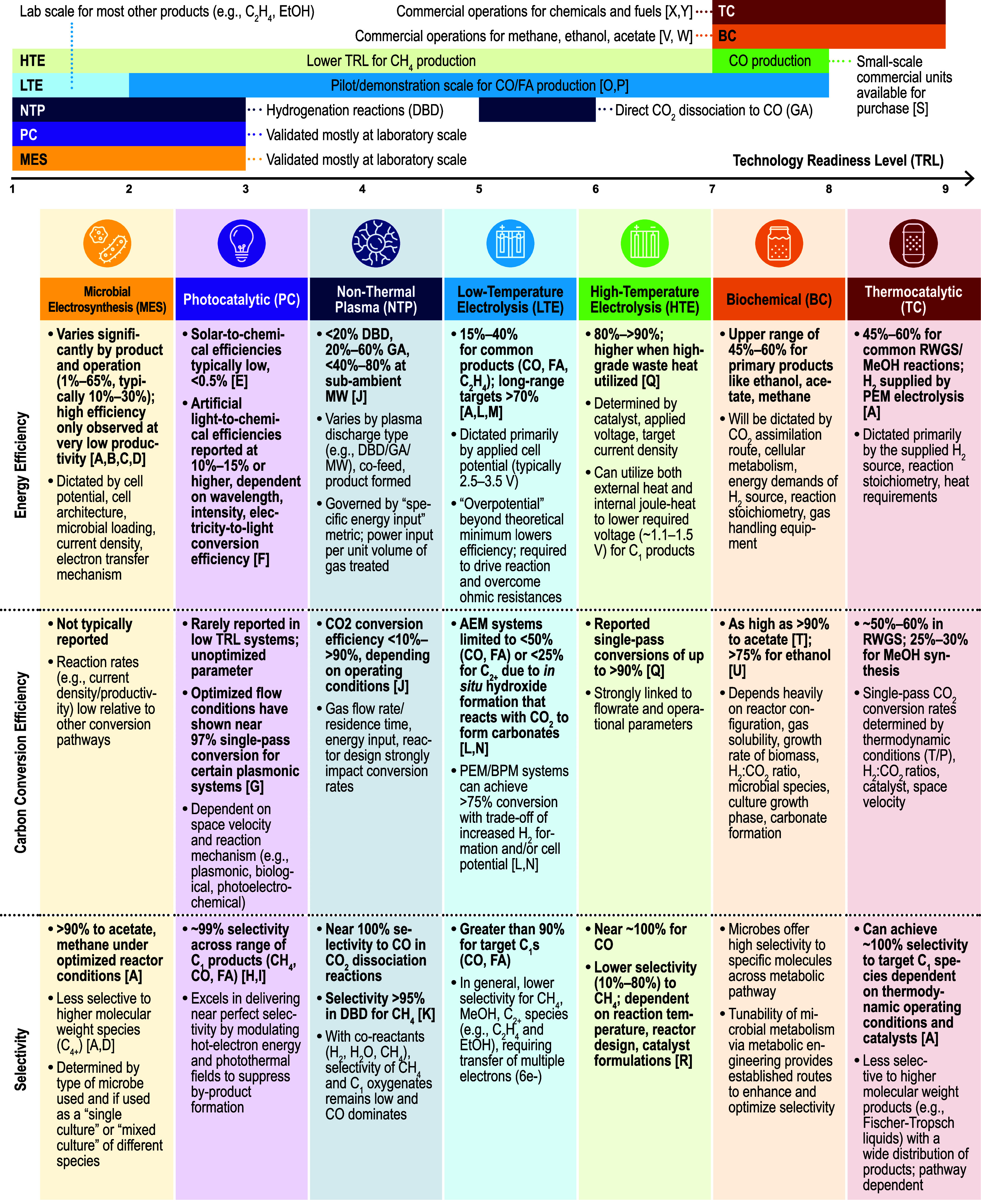
Comparative overview of cross-cutting technical performance metrics. references: [A],^4^ [B],^202^ [C],^370^ [D],^371^ [E],^372^ [F],^177^ [G],^373^ [H],^374^ [I],^375^ [J],^123^ [K],^124^ [L],^376^ [M],^377^ [N],^7^ [O],^378^ [P],^379^ [Q],^380^ [R],^381^ [S],^382^ [T],^383^ [U],^384^ [V],^385^ [W],^386^ [X],^387^ [Y][Bibr ref388]

Further, as performance metrics may be aggregated across multiple studies, care should be always taken when correlating all of these performance metrics together. For example, systems operating at state-of-the-art efficiency may not be able to achieve the reported state-of-the-art durability which can create challenges with appropriately understanding the state of these technologies and representing them in technoeconomic analyses or other high-level representations. Many of the metrics and technologies summarized in Figure 20 are specific to laboratory scale systems yet mature technologies will be manufactured and deployed at scales significantly larger than current R&D scale systems. This need for manufacturing scale up, while qualitative, does serve as an important control on the optimal performance metrics for each conversion technology. The potential need for large-scale deployments of these technologies and the importance of manufacturing economies of scale for capital cost reductions means that R&D must target advances that can be manufactured at scale.^369^ For example, R&D low-temperature electrolyzer membrane electrode assemblies (MEAs) are generally manufactured using a hands-on ultrasonic spray coating process while commercial MEAs are typically manufactured via continuous throughput roll-to-roll processes. New materials and MEA designs for low-temperature electrolyzers must ensure that these advances are relevant to at-scale manufacturing processes.

### 5.1 Energy Efficiency

As stated throughout the review, energy efficiency is one of the key defining metrics of CO_2_ conversion. Starting from a point of no intrinsic energy content (i.e., heating value) and predominately comprised of oxygen, a substantial amount of input energy—in the form of electricity, heat, and/or hydrogen—is required to conduct the necessary reaction steps to produce most CO_2_-derived products at scale. Consequently, the economic viability and competitiveness with incumbent methods is inextricably linked to future assumptions around the cost and availability of the supplied energy and, correspondingly, the efficiency at which it is consumed.

Across the seven considered pathways, energy is generally consumed in one of three ways: directly via electrons (LTE, HTE, NTP, MES), indirectly through low-carbon hydrogen (TC, BC), or via light (PC). For electrolysis and microbial electrosynthesis pathways, energy input is primarily based on the applied cell potential and the subsequent charge delivered to the surface of the electrocatalyst. Shown in Figure 20, for LTE and MES, the applied voltage in these systems typically ranges from 2.0 – 4.0 V, depending on the operating conditions, target product, and desired productivity (i.e., current density). When compared against the theoretical minimum voltages based on thermodynamic principles, these reported voltages are notably higher, needing additional driving force to overcome internal resistances and drive the kinetics at meaningful rates. These inefficiencies lead to reported energy efficiencies in the range of ∼ 10–40%. As these systems mature over time, it is expected that voltages will fall and efficiencies will improve; however, with more mature H_2_O electrolysis systems showing long-term targets of ∼ 70%, it is reasonable to expect that CO_2_-focused electrolysis may find a ceiling around similar, or lower, levels depending on product. A notable standout to the upside is high temperature electrolysis. By operating at high temperature regimes (typically 800 – 850 °C) and through leveraging internally generated joule heat, HTE electrolyzers can operate with a much lower overall voltage demand in the range of ∼ 1.2 – 1.5V, leading to observed energy efficiencies in some cases exceeding 95%.^105^ Like electrolysis, nonthermal plasma pathways similarly experience a wide range of observed energy efficiencies from as low as < 20% to approaching 80% depending on tradeoffs around reactor configuration, specific energy input, residence time of the reactants, and other technical parameters.

In the case of H_2_-mediated CO_2_ conversion, pathway energy efficiency is primarily controlled by two parameters: (1) the efficiency of producing the H_2_ feedstock and (2) the efficiency in which the H_2_ is utilized for CO_2_ conversion. While there are many routes to producing H_2_ (see: Table 4) and their review is outside the scope of this work, commonly cited low-carbon H_2_ sources include PEM and alkaline systems with typical operating energy efficiencies in the range of ∼ 55–65%. From this starting point, it places an inherent cap on energy efficiency from H_2_-mediated CO_2_ conversion processes. Further, in the conversion (e.g., methanol synthesis, ethanol fermentation, methanation, etc.) there are also unavoidable stoichiometric inefficiencies in how H_2_ is ultimately consumed within the reaction pathways. To effectively deoxygenate the CO_2_ feedstock and balance the overall reaction, water is typically formed as a byproduct as shown in Equations 7 – [Disp-formula eq9], where for some products (e.g., methane, ethanol) up to 50% of the incoming H_2_ goes toward the formation of H_2_O that provides little to no value. Therefore, often only a fraction of the incoming H_2_—produced with a typical efficiency of 55–65%—may end up in the final product, further limiting the maximum possible CO_2_ to product energy efficiency. Shown in Figure 20, while also product dependent, estimates across thermo and biocatalysis typically fall in the 45–60% net energy efficiency range.

7 $$CO_{2}+3H_{2}\to CH_{3}OH+H_{2}O$$

8 $$CO_{2}+4H_{2}\to CH_{4}+2H_{2}O$$

9 $$2CO_{2}+6H_{2}\to C_{2}H_{5}OH+3H_{2}O$$

Lastly, the remaining major mechanism for energy transfer considered herein is light, either in the form of direct solar-to-chemical conversion (i.e., natural light) or artificial illumination. Current state of the art data reflected in Figure 20 reveal solar-driven photocatalytic systems typically exhibit overall efficiencies below 1%, whereas artificially illuminated systems can achieve 10–15% light-to-chemical efficiencies, though these values remain highly sensitive to spectral distribution, photon flux, and catalyst architecture. Looking ahead, improvements in light-source efficiency and photonic utilization offer a path toward higher-performing photodriven CO_2_ conversion. Continued advances in highwall-plug efficiency LEDs and better spectral matching between emitters and catalyst absorption bands will reduce optical and electrical losses, while emerging plasmonic, photonic, and nanostructured catalyst designs promise greater light assimilation and more efficient carrier generation.

### 5.2 Carbon Conversion Efficiency

Another vector for consideration in CO_2_ conversion is carbon conversion efficiency. With many of the commonly proposed sources of CO_2_ feedstock being dilute by nature (e.g., < 15% CO_2_), such as direct air and point source industrial capture, the costs to sufficiently concentrate and utilize CO_2_ can be high (see: Figure 26). Achieving high rates of carbon conversion is therefore key to efficiently utilizing the feedstock and minimizing process operating costs associated with purification, recapture, or recycling of unconverted CO_2_. Further, from the perspective of emissions reductions, mitigating the rerelease of unreacted carbon can also carry significant life-cycle implications.

Highlighted in Figure 20, reported single pass conversion efficiency varies widely by pathway, from less than 5% to exceeding 97%. In the lowest TRL pathways (e.g., MES, PC) conversion is often not reported, with current research instead favoring other core “proof-of-concept” metrics such as productivity or energy efficiency. At the other end of the TRL spectrum, technically mature and optimized pathways such as thermocatalysis or biocatalysis commonly report a wide range for carbon conversion, often dictated by intrinsic thermodynamic or biological limitations. For example, in the case of industrially practiced equilibrium reactions such as water-gas shift and methanol synthesis, thermodynamics constrain single-pass conversion rates of these reversible reactions to typically ∼ 50–60% for CO and ∼ 25–30% for MeOH, depending on the specific operating conditions. In the case of biological conversion, the conversion of CO_2_ to products is similarly intrinsically limited, but rather by the metabolism of the microbe itself and the amount carbon diverted away from products to maintain cellular growth and reproduction. Typical conversion values for common biological products range from 75% to 90% for ethanol and acetate, respectively. For other moderate TRL pathways (e.g., LTE, HTE, NTP), carbon conversion is largely a tunable parameter without rigid thermodynamic limits. Rather, single-pass carbon conversion is flexible based on the reactor design, electrolyte chemistry, and other operating conditions of a process. Ultimately, for these pathways the ideal target carbon conversion efficiency is likely to be case dependent, requiring consideration of the interplay between a combination of core operating parameters such as CO_2_ cost, space velocity, equipment sizing, selectivity, and input energy.

### 5.3 Product Selectivity

In general, the most commonly targeted products or intermediates via CO_2_ conversion include C_1_ species such as methane, carbon monoxide, methanol, or formic acid. Not only do these products represent core building blocks across the chemicals and fuels industry, but C_1_ products—especially those containing oxygen—are mechanistically easier to produce compared to multicarbon species. Indeed, each of the seven pathways considered have promising routes to producing C_1_ molecules with selectivity exceeding 90%.

As product carbon number increases, the reaction mechanism(s) required to sequentially combine and link multiple carbon atoms in series becomes increasingly more complex and pathway-specific differences become more readily apparent. Some pathways, such as the high temperature electrolysis pathway, are considered unsuitable to form C_2+_ species altogether due to thermodynamic instabilities arising from high operating temperatures in O-SOECs. For most other pathways, C_2+_ species are possible, albeit at reduced selectivity relative to C_1_ production. A notable example is low temperature electrolysis over Cu electrocatalysts where one study showed mixtures of up to 16 different products can be produced simultaneously, with 11 out of 16 being C_2+_. However, with the selectivity toward most of the observed C_2+_ species being < 1%, economic separation and recovery becomes challenged.^46^ Biochemical conversion pathways, however, are a standout exception to this rule of thumb on selectivity. Leveraging specialized metabolic pathways (e.g., Calvin-Benson Bassham, Wood Ljungdahl, Reductive TCA, etc.), biological routes can assimilate CO_2_ to larger multicarbon products (C_2_ – C_18+_) often with near 100% selectivity. This is a key advantage for biochemical pathways and creates opportunities for process intensification where high molecular weight biopolymer or fuel-range molecules can be produced directly with fewer processing steps.

### 5.4 Pathway Strengths, Active Research Areas, and Scalability

#### 5.4.1 Microbial Electrosynthesis

The field of microbial electrosynthesis sits at the intersection of electro- and biochemistry. This marriage of technologies offers the potential for several advantages including the ability to rapidly cycle on/off to follow electrical loads at periods of low-cost energy, the ability to leverage biological CO_2_ fixation chemistry to directly produce higher molecular weight species with high selectivity, and high­(er) contaminant tolerance and ability to interface with a more diverse set of lower purity substrates than electrolysis alone. Indeed, many of the prevailing proposed use cases of MES (e.g., wastewater upgrading) offer a unique opportunity space for MES relative to other feedstock-sensitive abiotic pathways.

However, owing to its low technical maturity, the MES pathway faces a myriad of both fundamental and technical challenges. The linking of both electrochemical and biochemical reactions makes for a complex network of possible electron transfer mechanisms (e.g., direct and extracellular) where the fundamental underlying mechanism is not yet fully understood and remains an active area of research. Further, MES pathways have so far demonstrated only very low productivity relative to more mature electrolysis processes. Specifically, the best-in-class reported current densities are typically on the scale of only ∼ 10–20 mA/cm^2^ or less whereas commercial electrolysis systems are 2 orders of magnitude higher (>2,000 mA/cm^2^). Thus, without significant technological advancements, the surface area, and consequently capital costs, could be prohibitively high to operate MES at commercially relevant scales and rates. These challenges in productivity stem both from reactor engineering issues (e.g., higher internal resistances of biofilms) and inherent biological limitations around the rate at which microbes can utilize electrons.

#### 5.4.2 Photocatalysis

Photocatalysis represents a unique but low TRL pathway for CO_2_ conversion, offering many similar strengths to that of LTE, MES, and NTP with rapid cycle times, scalable and modular reactor architectures, and near-ambient operating conditions. A key differentiator, however, lies in the ability to utilize light—natural or artificial—as the reducing mechanism. In this manner, photocatalytic pathways are uniquely equipped to transform CO_2_ into products without the typical external electrical input (or H_2_ cofeeds), providing novel opportunities in siting and the ability to utilize the limitless resources of our sun. Further, using next-generation catalysts known as plasmonics, high-intensity light at specifically tailored wavelengths can be utilized to selectively excite photoreactive catalysts to drive chemical reactions versus classical reactions which rely on heating and/or pressurizing the entire reactor volumes.

Significant R&D, however, is still needed to bridge the gap between lab-scale novelties to commercially viable systems. One of the most significant barriers is the typical low single-digit energy efficiency at which light is converted into products, creating headwinds in terms of equipment sizing to reach comparable and relevant production scales. As such, more fundamental research is needed to better understand the complex reaction mechanisms underpinning the light-to-chemical conversion step(s). Long-term durability studies are another critically needed area of research to address known issues related to photocorrosion of nanoparticle catalysts and light-induced phase change of materials that currently present barriers to achieving long-term stability.

#### 5.4.3 Non-Thermal Plasma

Situated between laboratory and pilot scale, NTP is a moderate TRL pathway that leverages the ability to selectively excite electrons to a state of high-energy nonlocal thermodynamic equilibrium, creating ionized gases and short-lived radical species conducive for C = O bond cleavage and CO_2_ conversion. Unlike conventional pathways which employ heat and pressure to drive chemical reactions, NTP can operate at near-ambient conditions using exclusively electrical inputs or can generate heat without an external heat source, as is the case for warm discharges. Certain NTP reactor designs, such as DBD, can also be combined with heterogeneous catalysts in hybrid systems to take advantage of the reactive species inside the plasma plume to drive targeted chemistries with secondary reactants (e.g., H_2_, CH_4,_, C_2+_ hydrocarbons, H_2_O, etc.), although research is needed to steer the selectivity toward desired products beyond CO and H_2_.

Due to the short-lived nature of radicals and highly reactive nature of species produced inside of the plasma zone, one of the major challenges often cited for NTP is rapid recombination of reactants leading to low overall energy efficiency. For this reason, the production of C_2+_ species is similarly challenged due to the number of necessary steps required to sequentially link carbon atoms relative to the lifetime inside the plasma zone. R&D around novel reactor architectures to maximize the lifetime and utility of these reactive species and improve energy efficiency is underway; however, many of the reactor designs tested to date (e.g., gliding arc, microwave) have attributes that make scaling for large-scale deployment challenging (e.g., subambient pressure operation for hitting energy efficiency targets). New developments in reactor designs (e.g., ambient operation of MW systems) shows promise in efficiency improvements that also lend themselves toward scalability.^389^ Further efforts to better understand the tradeoffs between reactor configuration and how best to optimize performance for potential scale up are an area of need. Finally, while NTP scale through a modular configuration in similar methods to LTE or PC (as exemplified by commercially available ozone generators), challenges with low selectivity when producing C_2+_ species can cause costly separations steps that may limit deployment.

#### 5.4.4 Low-Temperature Electrolysis

Low temperature electrolysis represents a moderate TRL pathway for CO_2_ conversion with recent emerging pilot and precommercial activity in the space of C_1_ chemistry. Some of the defining strengths of LTE include the (1) near-ambient operating conditions, (2) the potential for rapid on/off cycling, (3) modular “stack” designs where the size of electrolyzers can easily be scaled up or down to meet product demands, and (4) a wide and tunable product distribution. Indeed, potential product diversity is a key feature of LTE where, through careful manipulation of the applied voltage and metallic electrocatalyst environment, literature reports find that over 20+ unique species are possible across multiple carbon numbers (C_1_-C_4_) and chemical functionalities (alkanes, olefins, alcohols, glycols, acids, ketones, and aldehydes), making it the most diverse product slate of all the direct electron consuming pathways (i.e., LTE, HTE, MES, NTP).

Before these strengths can be fully realized at commercial scale, further R&D and engineering is needed to address several critical needs in LTE. First, although the known list of possible products is large, the selectivity at which C_2+_ species are produced is often low due to complex reaction and coupling mechanisms, contributing to prohibitively expensive purification and recovery costs. Further, to minimize selectivity challenges related to parasitic hydrogen generation side reactions, many studies opt for highly alkaline electrolytes. While successful at suppressing hydrogen formation, the alkaline conditions can result in up to 50–75% of the incoming CO_2_ being consumed in the formation of carbonates, leading to low per-pass carbon conversion and membrane crossover issues. Finally, platinum group metal use and corresponding high capital costs of LTE stacks combined with poor long-term durability of the catalyst and stack componentry creates headwinds that challenge economic viability and act to limit the ability of electrolyzers to operate at low capacity factors otherwise needed to utilize low-cost electricity.

#### 5.4.5 High-Temperature Electrolysis

Of the electrolysis-based pathways, high-temperature solid oxide electrolysis currently represents the most technically mature and commercially accessible option. Indeed, modular SOEC units have been deployed commercially for applications in space exploration and chemical synthesis.^30^ Highlighted in Figure 20 and Figure 21, one of the main advantages cited for HTE pathways is the potential for energy efficiencies approaching 90%—or higher when high-grade waste heat is available (e.g., nuclear waste heat). In the current economic climate where energy demand is expected to grow meaningfully and therefore put upward pressure on energy costs into the foreseeable future, HTE presents a compelling option for minimizing energy costs with a technology that has been largely derisked.

**Figure 21:**
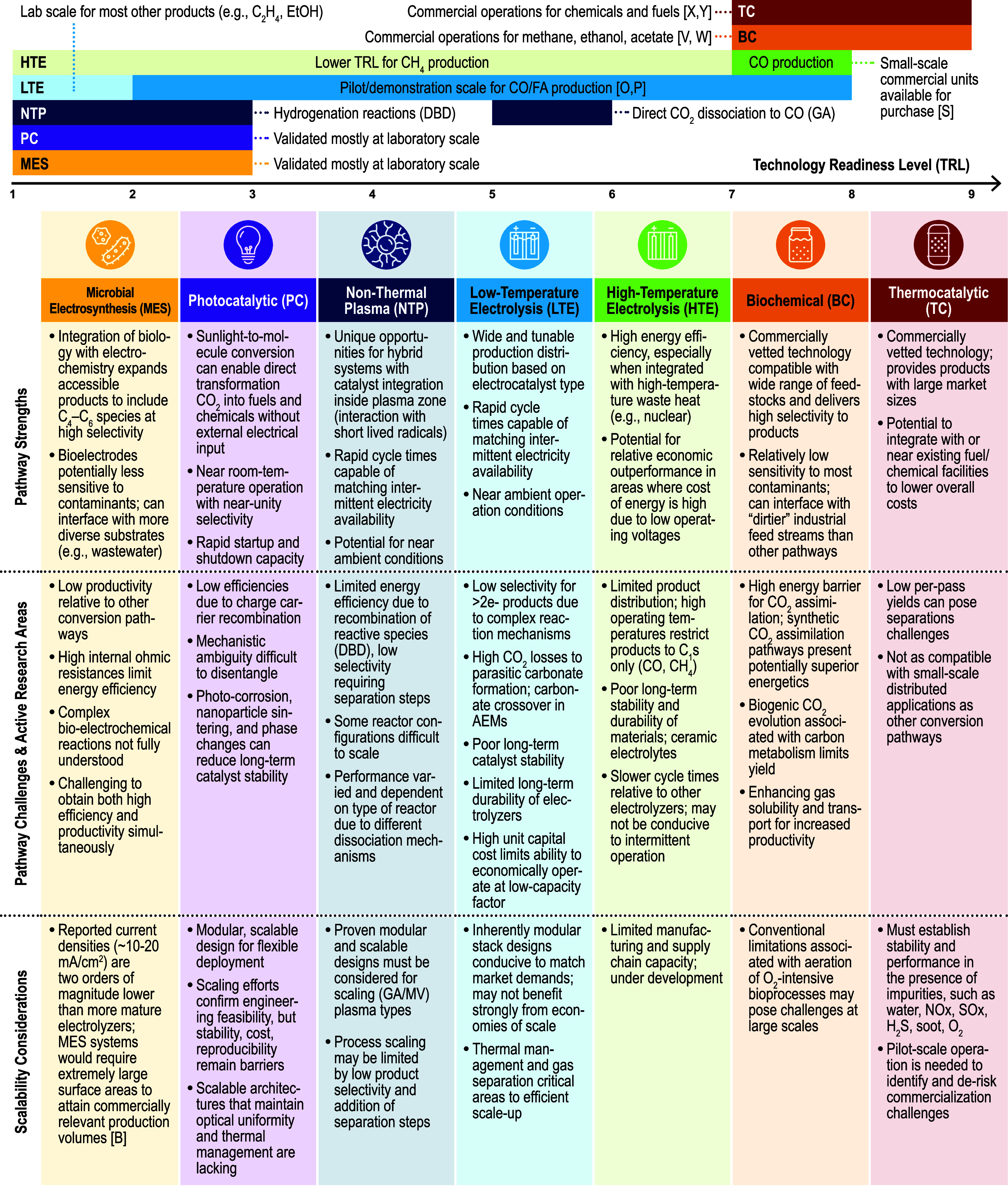
Comparative overview of pathway challenges and opportunities. References: [B][Bibr ref202] [O],^378^ [P],^379^ [S],^382^ [V],^385^ [W],^386^ [X],^387^ [Y][Bibr ref388]

While the uniquely high operating temperatures translates to benefits in operating efficiency, it is not without tradeoffs. One of the most significant drawbacks to high operating temperature is the impact on product formation. Whereas pathways operating at near-ambient conditions have access to a wide and often tunable set of possible products spanning multiple carbon numbers, the high temperature nature of SOECs is thermodynamically unfavorable for C-C bond formation, causing intermediates to rapidly decompose into exclusively C_1_ products, specifically methane and carbon monoxide. Another consequence of high operating temperatures is less flexibility in potential on/off cycling or electrical load following. Whereas lower temperature systems may be more easily cycled to potentially follow daily or hourly fluctuations in the electric grid, high temperature electrolyzers intentionally avoid large or frequent temperature swings to minimize thermal stresses and durability issues with the comprising ceramic and metal componentry. Instead of completely cycling on/off, HTE systems often favor a “hot standby” mode where the electrolyzer is held at elevated temperature without producing a product; however, prolonged duration in this state has a negative impact on energy efficiency.

#### 5.4.6 Biochemical Conversion

Biochemical fermentation represents the highest TRL and commercially vetted biotic CO_2_ conversion pathway. Leveraging similar natural and man-made CO_2_ assimilation pathways as in the case of MES, biocatalysis offers routes to unique and diverse sets of products—in particular acids and alcohols—with high selectivity. Further, being a purely biological route without reliance on metal catalysts/electrocatalysts, biocatalytic systems show the highest resistance to contaminants in feed streams, making it an excellent pathway for pairing with a wide variety of CO_2_ point sources, requiring minimal preprocessing or clean up.

Most of the significant remaining challenges surrounding biological conversion involve hydrogen. Specifically, to convert CO_2_ biologically in the absence of other carbon oxide or energy carrying species (e.g., CO, formic acid, sugar), external H_2_ is typically a required cofeed to supply reducing energy. Thus, the competitiveness of biocatalytic systems is linked to the source of H_2_ and the associated cost, carbon intensity, and efficiency at which it is sourced. Furthermore, ongoing research continues to explore novel ways of increasing gas–liquid mass transfer to ensure effective uptake and utilization of the CO_2_ and H_2_ gases by the microorganisms, a key to enhancing overall productivity.

#### 5.4.7 Thermocatalysis

Across the seven considered pathways for CO_2_ conversion, thermocatalysis represents the most mature and commercially accessible option for near-term adoption. Benefitting from decades of R&D and infrastructure investments by the petroleum industry, a broad suite of thermocatalytic reactions and processes relevant to CO_2_ conversion (e.g., CO_2_ hydrogenation) have been derisked and commercially vetted at scale. Through these pathways, thermocatalysis offers the potential for industry to leverage off-the-shelf ready technologies and access a range of high-volume products spanning alcohols, olefins, and fuels.

Despite the underlying chemical reaction mechanisms involved with CO_2_ thermocatalysis being well understood and largely optimized, TC processes can still face unavoidable thermodynamic barriers. As noted earlier, low per-pass conversion of equilibrium reactions can increase operational complexity and raise costs related to product separation and recycle. Further, with capital equipment costs being strongly correlated to economies of scale, TC processes are most often operated at very large, centralized facilities. Relative to other, more modular CO_2_ conversion designs, this intrinsic scaling behavior makes TC pathways potentially not as competitive in small-scale distributed CO_2_ conversion applications. Finally, unlike the biological systems which can excel at handling raw, or minimally processed waste gas streams, the metallic catalysts used in TC can be more sensitive or easily poisoned by some common contaminants (e.g., sulfur containing compounds) found in CO_2_ point sources. Thus, TC pathways require careful consideration on the source of CO_2_ and strategies to mitigate the impacts of common impurities (e.g., NO_x_, SO_x_, H_2_S, Soot, O_2_, etc.).

## 6 Logistics for CO_2_ Conversion Chemistry and Barriers to Scale Up

Sections 2–5 have focused on emerging and developing chemistries for the conversion of CO_2_ into value-added products. While significant R&D opportunities exist in the realm of performance, scale-up, and long-term durability of these pathways, it is important to also acknowledge the feedstocks these processes require and how the governing costs and logistics of sourcing said feedstocks impact the viability, optimal design, and integration of conversion pathways. These considerations are the focus of Section 6, where we review major feedstocks used in CO_2_ conversion pathways, summarize market and production scale considerations for these technologies, review the current state of commercial adoption of CO_2_ conversion technologies, and conclude with major takeaways and challenges associated with these early stage demonstrations.

### 6.1 Sourcing Electricity

Whether seeking to minimize the cost of production or life cycle impacts of a CO_2_-based product, the source and availability of electricity is nearly always the principal consideration.^18,25,390^ As such, strategies to drive down levelized cost of production through integration of low-cost and low-carbon sources of energy often act as controls on how, when, and where CO_2_ conversion technologies can be deployed. At the level of this review, we find two major considerations for electricity sourcing for CO_2_ conversion: 1) spatial and temporal dynamics of renewable generation and how downstream loads might optimally integrate, and 2) bulk availability of low-cost, low-carbon intensity electricity and competing uses and emerging large loads. This section discusses considerations for electricity sourcing regardless of if the CO_2_ is consumed directly (e.g., in electrochemical/NTP CO_2_ reduction) or indirectly using an energy-dense feedstock (e.g., CO_2_ hydrogenation/fermentation mediated with H_2_ produced via electrochemical water splitting).

Several potential approaches exist for supplying renewable electricity to CO_2_ conversion. Wind and solar photovoltaic (PV) generation are the lowest-cost source of new renewable generation in many locations in the United States, but their generation is variable based on wind speeds and solar irradiance, respectively, factors that shift on a diurnal and seasonal basis for a given location. Balancing direct input energy from renewable generation requires some form of storage either in the form of battery energy storage or physical intermediate storage (e.g., hydrogen storage). Regardless of the approach, these needs increase the overall project capital costs, invoking a tradeoff between capital costs of equipment and operating costs associated with electricity consumption that will be discussed further in Section 6.4. CO_2_ conversion might also be integrated into the electric grid to balance generation and load, but grid integration requires consideration of the carbon intensity of the grid generation mix at a given time and location and subsequent impacts to the carbon intensity of CO_2_ based products. The possible combination of electricity generation and storage technologies is long – there is likely not a one size fits all solution for all locations and conversion pathways as shown in Figure 22, but electricity supply is a critical factor for the feasibility of CO_2_ conversion pathways. In the remainder of this section we discuss broad trends in electricity generation and storage and in grid infrastructure and future large loads in the context of the technologies reviewed in Sections 2–5.

**Figure 22:**
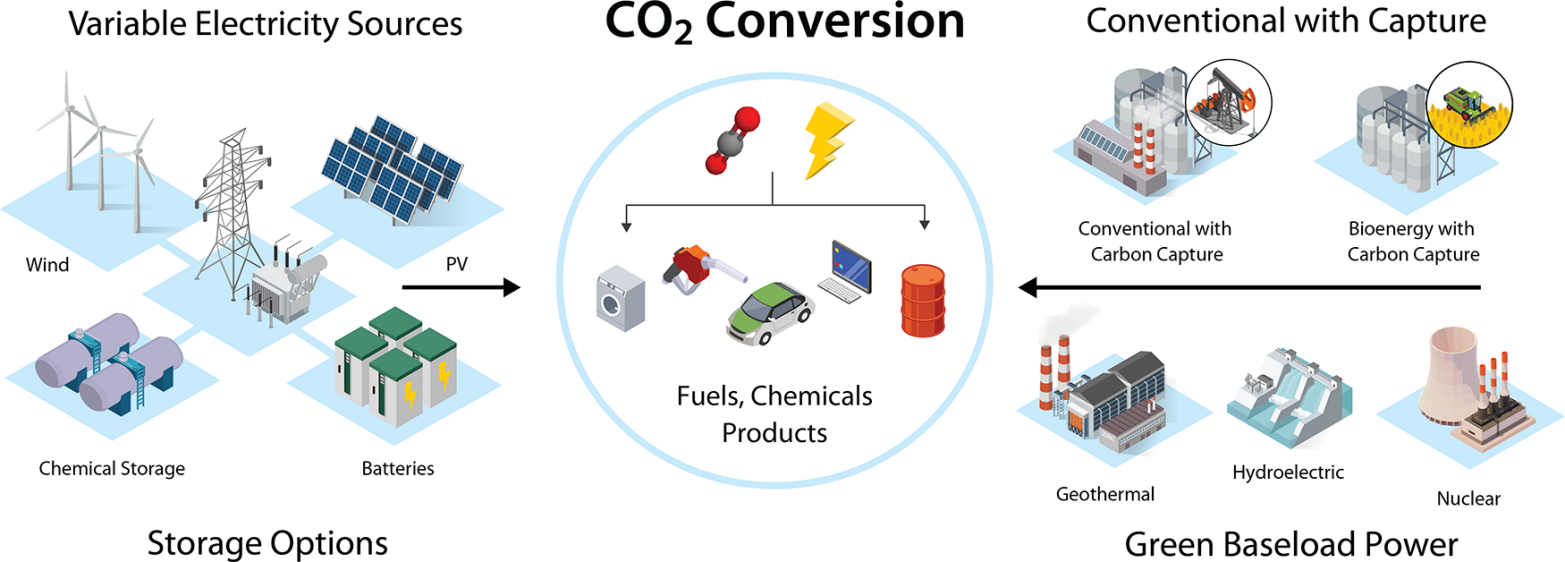
Many modes of supplying electricity for CO_2_ conversion

In the past five years, the cost per unit energy ($/MWh) for new wind and PV has continued to fall^391^ and rates of deployment for these technologies continues to increase and become a larger share of installed generation capacity in many countries.^392^ Deployment of variable renewable energy (VRE) resources is supported by recent decreases in the cost of short-duration battery energy storage that can help balance daily mismatches between generation and load, especially in the case of PV for shifting generated energy later in the day to meet evening peak demands.^393^ These technologies for generation and storage are being deployed at a time when grid operators are anticipating increased loads across sectors, including data centers,^394^ cryptocurrency mining, light-duty transportation electrification,^395^ and production of hydrogen. Understanding these dynamics is critical for CO_2_ conversion pathways that are likely to be large consumers of VRE either directly behind the meter or indirectly through integration with evolving grid infrastructure.

Being potential large consumers of electricity, it is paramount for CO_2_ conversion pathways to understand the macro trends influencing the evolution of grid infrastructure and energy markets because these trends drive how conversion pathways might chose to interact with the evolving electricity system. For example, deployment strategies for in front of the meter (connected with the electric grid) or behind the meter (direct connection with generation technology and isolated from the broader grid) energy supply for CO_2_ conversion will drastically impact how one might choose to operate the system as well as the capital investment required for intermediate storage of energy or products and reactants. Given the multitude of options available and region-specific sensitivities, this review does not set out to prescribe a best-case approach for electricity sourcing. At any rate, the best-case will very likely vary from technology to technology, location to location, and what changes the grid has undergone that impact future large loads.

To provide several illustrative examples of regional and technologically dependent CO_2_ conversion pathways, we summarize selected recent work in this area, but readers should note that this is a rapidly evolving field. Recent U.S. focused work has evaluated different regions as potential hubs for CO_2_ conversion based on the availability of CO_2_ feedstocks and low-cost grid electricity.^396^ Key considerations and takeaways from this work include the importance of regional grid mixes and power markets, variable costs of power purchase agreements (PPAs), dynamic wholesale power markets, and generation assets and changing demand mixes in each region influence the cost of accessing electricity at MW to GW scales. As such, for grid-tied (in front of the meter) CO_2_ conversion technologies, an understanding of regional grid assets, transmission infrastructure, and applicable utility rate structure dictate design components and operational dynamics of the conversion pathway is critical. Considering recent work analyzing behind-the-meter systems producing hydrogen, major implications include the importance of complementary wind and solar PV mixes, increasing generation and therefore electrolyzer capacity factors and capital utilization.^397^ Most large scale CO_2_ conversion technologies powered with behind the meter VRE are likely to require some form or storage to smooth output, either in the form of electrical energy storage or physical product and feedstock storage. The favorability of using either or both storage technologies is determined by local VRE resources, capital costs of system equipment, and flexibility in product offtake.

Regardless of variance in system design and integration strategies, one certainty is that as CO_2_ conversion chemistries continue to mature and are derisked, their scalability and impact in the marketplace will ultimately be tied to the amount of available low-carbon electricity. In Figure 23 future projections for the total global electricity generation in 2030 and 2050 as reported by IEA and IRENA are shown. The data encompasses scenarios (e.g., STEPS/PES) reflective of the stated policies and plans by governments, as well as scenarios (e.g., NZE, 1.5C) corresponding to a deeper electrification of the global economy with the incorporation of constraints on system-wide emissions. Based on these projections, estimates suggest that globally in 2030 the total amount of renewable electricity (incl. nuclear) may range from 20,000 TWh in the conversative scenarios to 31,000 TWh in the aggressive electrification scenarios. The figure for renewables increases in 2050, reaching 47,000 TWh to 86,000 TWh in the conservative and aggressive scenarios, respectively.

**Figure 23:**
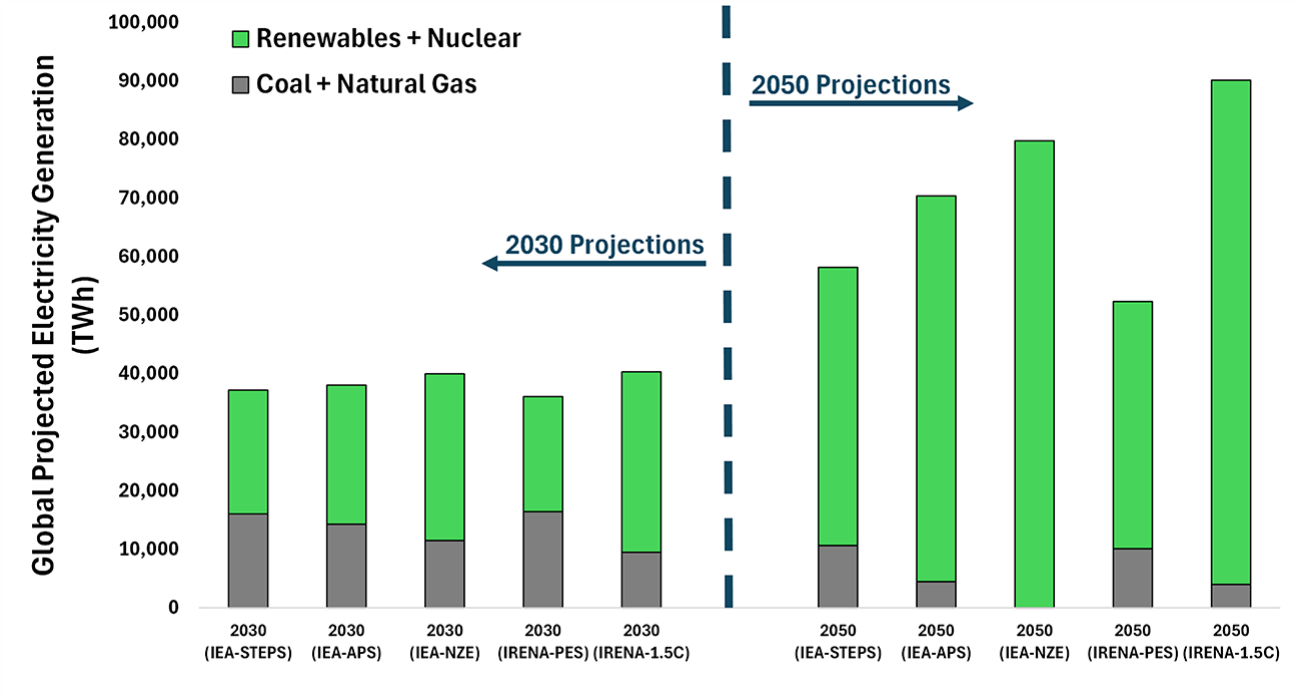
Projected electricity generation by source in 2030 and 2050 as reported by IEA and IRENA. STEPS = Stated Policies Scenario, APS = Annouced Pledges Scenario, NZE = Net Zero Emissions by 2050 Scenario, PES = Planned Energy Scenario, 1.5 = 1.5 Degree Celsius Scenario. Data from refs 398 and 399.

Being still only a fledgling industry, as of 2024 CO_2_ conversion and other “Power-to-X” pathways do not yet significantly contribute to the approximately 30,500 TWh consumed annually (2024) to sustain global business-as-usual activities. Thus, it is important to put in perspective how much of the projected renewable electricity generation would be needed to sustain the current economic status-quo versus the myriads of new use cases, such as CO_2_ conversion chemistry. Shown in Figure 24, when the total generation projections for 2030 and 2050 are compared against current 2024 consumption levels, the data reveal that depending on scenario, between 5,600 and 9,800 TWh may be added by 2030, reaching an additional projected 21,800 to 59,600 TWh by 2050.

**Figure 24:**
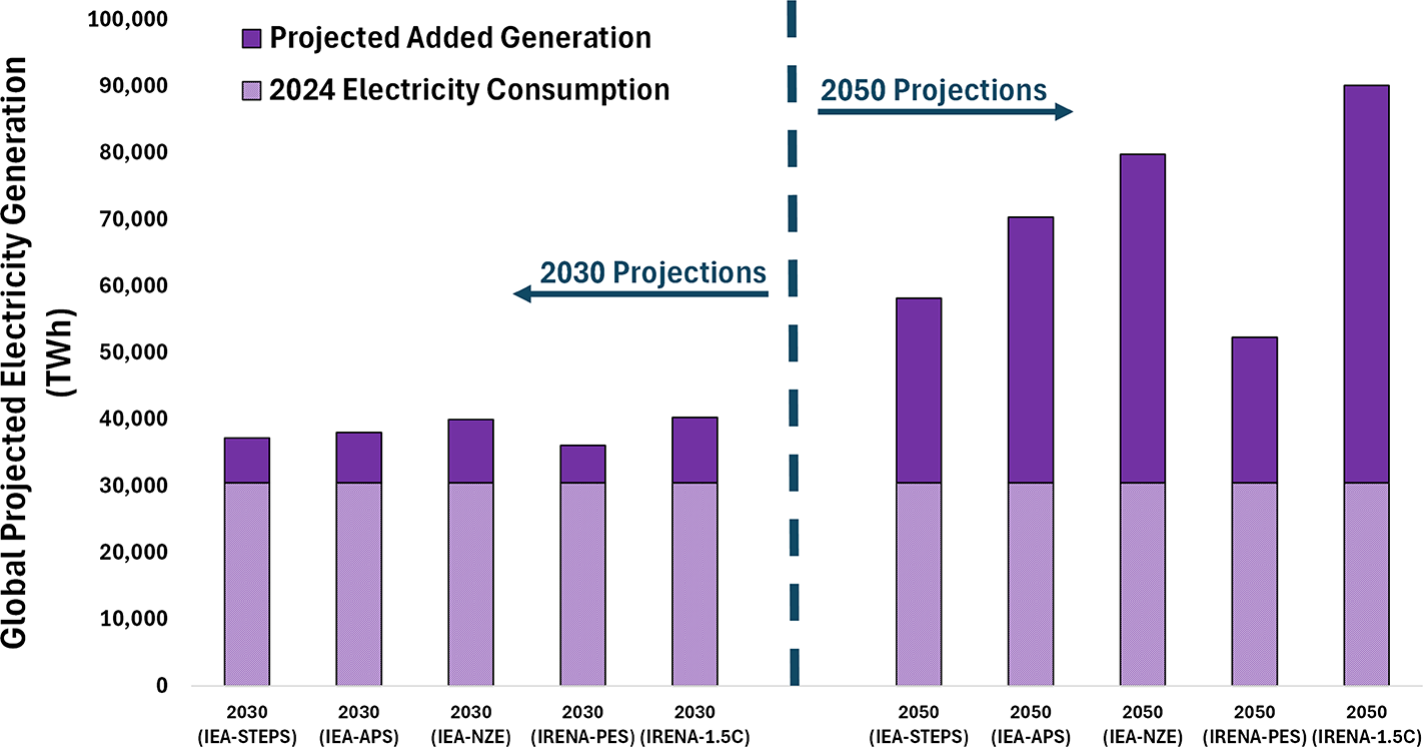
Projected additional electricity generation in 2030 and 2050 relative to 2024 levels. STEPS = Stated Policies Scenario, APS = Annouced Pledges Scenario, NZE = Net Zero Emissions by 2050 Scenario, PES = Planned Energy Scenario, 1.5 = 1.5 Degree Celsius Scenario. Data from refs 398 and 399.

A key question arising from these data is, given the strong demand for electricity across a multitude of different end markets, what fraction of the added electricity generation could feasibly be utilized for CO_2_ conversion, and what impact could that have on the existing chemicals and fuels market? Estimates show that global demand for electricity has accelerated in 2024 and is now expected to grow by an average of 3.4% per year through 2026, driven by strong growth in emerging economies as well as strong growth across data centers, AI, and cryptocurrencies.^400^ In fact, according to the IEA, it is estimated that over roughly the next decade approximately 67 – 81% of new capacity growth will be driven by light duty vehicle electrification, heat pumps, office and residential cooling, and other energy intensive industries (e.g., data centers, crypto mining, industrial heat) depending on the region and electricity scenario.^398^ Across the various electricity scenarios considered by IEA as shown in Figure 25, hydrogen production, an important input feedstock for many “Power-to-X” or “e-fuels” pathways, represents between 3.4% and 23.9% of the global added capacity, depending on scenario and region.

**Figure 25:**
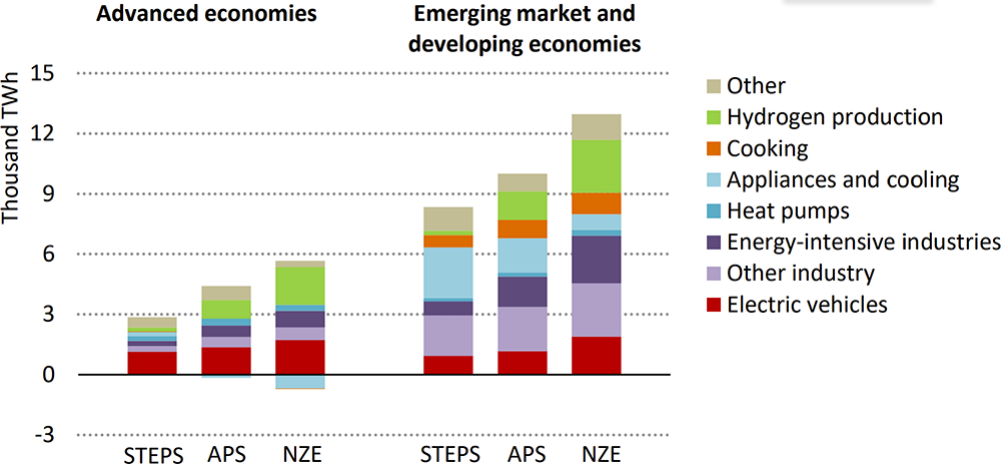
Electricity demand growth by application and IEA scenario from 2023 to 2035. Figure courtesy of IEA. Reproduced from ref 398.

Collectively, if it were assumed that all of the electricity allocated to “hydrogen production” were utilized for e-products, it is possible offer an order-of-magnitude estimate for the possible impacts on chemicals and fuels markets. In Table 3 we show the hypothetical electricity required to meet 100% of the projected 2030 market demand of four commonly cited CO_2_-based products, assuming an overall net 50% energy efficiency process. Note, as shown in Figure 20, actual energy efficiency varies widely between pathway and product. Herein, the 50% estimate is provided as a simplifying and illustrative benchmark only, representative of a hypothetical moderate TRL conversion pathway. Further, while we recognize that displacing 100% of any single chemical market by 2030 is unrealistic, this calculation nevertheless illustrates the potential impact CO_2_ conversion could have. Using this back-of-the-envelope approach reveals that meeting the global demand for any one of these common products in the stated plans and policies scenario (STEPS) would require from 630% to > 36,760% of the total electricity generation projected for “hydrogen generation” from 2024 to 2030. In the announced pledges scenario (APS), the requirements are reduced to 120% and 7,020%. In the most optimiztic NZE scenario, the requirements are 65% to 3,740%. Although only meant to serve as approximations, these data bring to light several important takeaways as it relates to sourcing electricity and the scale up of CO_2_ conversion:

(1) For many of the commonly cited possible products (Table 3), a CO_2_ conversion alternative would represent one of the most energy demanding processes ever recorded for making gaseous and liquid chemicals at the commercial scale. For perspective, at 50% energy efficiency the estimated energy intensity values of CO_2_ conversion products are in some cases > 3× that of modern “energy intensive” processes such as NH_3_ synthesis via Haber Bosch (∼30 GJ/tonne).
(2) Energy intensity can vary widely per product and is influenced by both the conversion specific factors such as applied voltages and selectivity but also the intrinsic chemistry (e.g., number of mols electrons/H_2_ utilized per molecule of product, final oxidation state, amount of retained oxygen, etc.).
(3) Based on current electricity forecasts for growth and utilization, CO_2_-based “e-products” are likely to comprise only a small fraction of fuels and chemicals production by 2030 if relying on existing installed generation capacity. Building dedicated new generation to meet these emerging demands is technically possible, but requires consideration of existing grid and energy infrastructure, and total available renewable resources.
(4) For e-products and CO_2_ conversion to make a significant impact on chemicals and fuels markets in the future, renewable electricity generation capacity and the associated transmission, storage, and other supporting infrastructure will likely need to be expanded meaningfully beyond even the most optimiztic scenarios currently proposed (e.g., deep electrification NZE, 1.5C).^401^

**Table 3:** Estimated Electricity Demand for Four Common CO_2_-Based Products

Product	Energy Intensity (GJ/tonne)^a^	2030 Market Consumption (MMT/y)^b^	2030 Electricity Demand (TWh)^c^	Fraction of 2030 Renewable Electricity for e-products (%, STEPS/APS/NZE)^d^
Ethylene	94.0	400	10,440	4510/860/460
Methanol	43.9	120	1,450	630/120/65
CH_4_ (RNG)	102.1	3,000	85,120	36760/7020/3740
PTL–SAF	101.0	350	9,820	4240/810/430

a Assumes a net energy efficiency of 50%. Values based on thermodynamic calculations.^4^

b Based on 2024 values and CAGR of 3%.

c Calculated from multiplying energy intensity and estimated 2030 market consumption.

d Represents what fraction of the available electricity generation attributed to “hydrogen production and e-products” in the IEA STEPS, APS, and NZE scenarios it would take to produce 100% of the 2030 market consumption of each product. For example, in the case of ethylene in the STEPS scenario, it would take 4510% of the planned electricity growth in e-products to produce 100% of the ethylene market in 2030.

### 6.2 Sourcing H_2_

Hydrogen is a critical chemical building block for many of the CO_2_ conversion processes reviewed here. Trends in hydrogen production, transmission and storage infrastructure build out, and end-uses are important considerations for any downstream users of hydrogen. The economics and life-cycle impact of processes that consume hydrogen often is driven by how, where, and when that hydrogen is produced, where aggregate statistics that capture these aspects are $/kg H_2_ levelized production cost (LCOH) and kg CO_2_eq/kg H_2_ produced, respectively. Along with these two metrics, the availability of hydrogen (i.e., steady state vs dynamic supply) and location of production is a key consideration for processes consuming hydrogen that will be discussed in this section.

There are a variety of possible technologies available for producing hydrogen, with some highlighted in Table 4. While an exhaustive evaluation of these production options is beyond the scope of this review and other literature has thoroughly reviewed production technologies,^402^ factors such as facility siting, operation, and economics do influence downstream users of the hydrogen, including CO_2_ conversion and are discussed here. Namely, the location, operational dynamics, and supporting infrastructure for these hydrogen production technologies are critical considerations for downstream users. For example, hydrogen production from water electrolysis that is powered by renewable electricity could be located on-site with the renewable generation but would need a colocated offtake for hydrogen or storage and transportation to enable moving hydrogen to the offtake. Depending on the offtake requirements, electrolyzer technology, and utility rate structure, hydrogen could also be produced at a steady state or dynamically as determined by renewable output or grid price signals in systems that are grid-tied.

**Table 4:** Summary of Existing and Emerging Hydrogen Production Technologies and High-Level Considerations.

Pathway [-]	Major resource requirement(s) during operational stage[-]	LCOH driver(s) [-]	Variable output notes	Demonstrated production scale [kg H_2_/day]
Low-temperature water electrolysis	Water, electricity	Cost of electricity and electrolyzer capital cost and durability	Variable operation for electrolyzers sourcing energy from behind-the-meter renewables or in dynamic grid pricing schemes	About 8,000 kg H_2_/day^a^
High-temperature water electrolysis	Water, electricity, heat	Cost of electricity, electrolyzer durability and efficiency	Current systems likely operate at steady state, although R&D is focused on electrolyzer stacks that enable dynamic operation	About 2,400 kg H_2_/day^b^
Steam methane reforming	Natural gas, dairy gas, landfill gas	Natural gas price	Generally operates at steady state with high capital utilization	About 50,000 kg H_2_/day
Geologic hydrogen	None	Unknown	Unknown	n/a
Biomass carbon removal and storage (BiCRS)	Woody biomass, municipal solid waste, agricultural residues	Feedstock cost, feedstock preprocessing, gasification performance	Generally operates at steady state with high capital utilization	Not yet commercially active

a Ref 410.

b Ref 411.

CO_2_ conversion technologies are not the only consumers of hydrogen today or in the future. Major consumers of hydrogen today include oil refineries and ammonia synthesis plants,^403^ although many other potential end-uses are under consideration such as medium- and heavy-duty transportation, steelmaking, and hydrogen for long duration energy storage in the electric power sector.^404^ As these end-uses evolve and develop, it is likely that not all of them will have the same willingness-to-pay for hydrogen.^403^ Therefore, the emergence of various markets for hydrogen will potentially depend on the delivered cost of hydrogen, its availability, and cost parity with any existing fuels that may already be used in a process or application. How hydrogen demand and markets evolve to meet increasing demand, and competition among competing end-uses are important considerations for CO_2_ conversion pathways that could be large-scale end-users of this molecule.

The evolution of LCOH from different hydrogen production technologies is a critical factor of consideration for CO_2_ conversion pathways that use hydrogen as a feedstock. Accurate estimates for LCOH in the near term and long-term future are important for informing development of CO_2_ conversion technologies. Quantification of these ranges is beyond the scope of this review, and instead we focus on qualitative cost drivers for major hydrogen production pathways (Table 4). The cost drivers of LCOH depend on the pathway, but for many the largest cost driver is the input feedstock, such as electricity for water electrolyzers, biomass for gasification, and natural gas for steam methane reforming. To provide readers with examples of high level considerations and cost drivers, the following paragraphs discuss considerations for low-temperature water electrolysis and steam methane reforming of natural gas, two of the most prevalent existing and emerging hydrogen pathways.

Technologies such as low- and high-temperature water electrolysis have significant R&D targets set across performance and cost metrics that, if achieved, would likely reduce the LCOH from these technologies.^405^ All of the considerations for electricity sourcing discussed in Section 6.1 apply to electrolyzers, with significant R&D focused on enabling these systems to reduce the cost of electricity and lower total LCOH.^406^ These efforts generally seek to optimize materials, performance, and cost to enable electrolyzers to operate dynamically based on direct VRE power input or dispatch the electrolyzer based on hourly grid pricing signals in wholesale power markets.^407^ Future deployments for water electrolyzers could be either located near low-cost electricity to minimize LCOH for production, or near offtake to minimize hydrogen transport and/or storage costs.

For natural gas based hydrogen production, the cost of natural gas feedstock generally is the largest component of LCOH. Given the large capital investment associated with SMR based hydrogen production and offtake requirements for hydrogen, these facilities tend to be operated at high capital utilization factors, are constructed at large scales, and are located near low-cost natural gas and hydrogen offtakes. For example, existing hydrogen production via steam methane reforming of natural gas for refinery applications is predominantly located in the Gulf Coast of the United States, using large scale facilities close to hydrogen offtakes. For SMR systems that are deployed with carbon capture and sequestration (CCS), the LCOH and total CI for the process will vary and are primarily subject to the cost of natural gas and upstream methane leakage rates and sourcing assumptions (e.g., domestic, imported LNG, etc.), respectively.^408,409^

An understanding of production integration opportunities, competition between end-uses, and evolution of hydrogen transportation and storage could serve consumers of the hydrogen such as CO_2_ conversion. As an example, while we noted that hydrogen produced from steam methane reforming of natural gas has conventionally been colocated with the hydrogen offtake, new infrastructure investments in pipelines and storage and LCOH reductions from water electrolysis could drive changes in future siting and hydrogen availability. It is possible that water electrolyzers could be installed where low-cost electricity is abundant, leveraging hydrogen pipelines to buffer dynamic hydrogen production while moving this molecule to various offtakes. How and where this infrastructure evolves is subject to notable uncertainty but this evolution and any resulting market development is an important consideration for CO_2_ conversion pathways.

### 6.3 Sourcing CO_2_

Beyond critical electricity and energy-carrying feedstocks like hydrogen and in some technology pathways methane, the final ingredient in CO_2_ conversion chemistry is CO_2_ itself, serving as the primary carbon building block for more complex products. Unlike typical commoditized chemical feedstocks that have robust and dedicated supply chains, CO_2_ is differentiated in that historically it has been viewed primarily as a waste byproduct to be released to the atmosphere with little to no market interest outside of smaller specialized cases such as the food and beverage industry or enhanced oil recovery.^412^

One of the current challenges contributing to the significant underutilization of CO_2_ is the cost of capture and concentrating dilute resources, whether they are from point source emissions or the atmosphere. Shown in Table 5, the concentration of available CO_2_ sources ranges from as low as ∼ 425 ppm in the atmosphere to near 100% in the case of select industries such as bioethanol fermentation. More often, however, point source concentrations fall in the 3–15% range. The corresponding cost to capture the CO_2_, shown in Figure 26, correlates with concentration with the high purity sources showing relatively low costs of capture of in range of $20-$30/ton and the more common dilute sources such as waste streams from coal and natural gas power generation at ∼ $50 to more than $150/ton CO_2_. In the most extreme case of dilute direct air capture, reported cost estimates can range from $230 to > $800/tonne depending on scale, technology, and assumed gains in technical performance.^413^

**Table 5:** CO_2_ Concentrations by Source, Modified from Ref 414

Process	CO_2_ Concentration (vol%)
**Power: Coal**	12–15
**Power: Natural Gas**	3–10
**Power: Fuel Oil**	3–8
**Cement Production**	20
**Refinery Operations**	3–13
**Integrated Steel Mills**	15
**Ethylene Production**	12
**Ammonia: Process**	100
**Ammonia: Fuel Combustion**	8
**Ethylene Oxide Production**	100
**Bioenergy**	3–8
**Fermentation**	100
**Atmospheric**	0.000425

**Figure 26:**
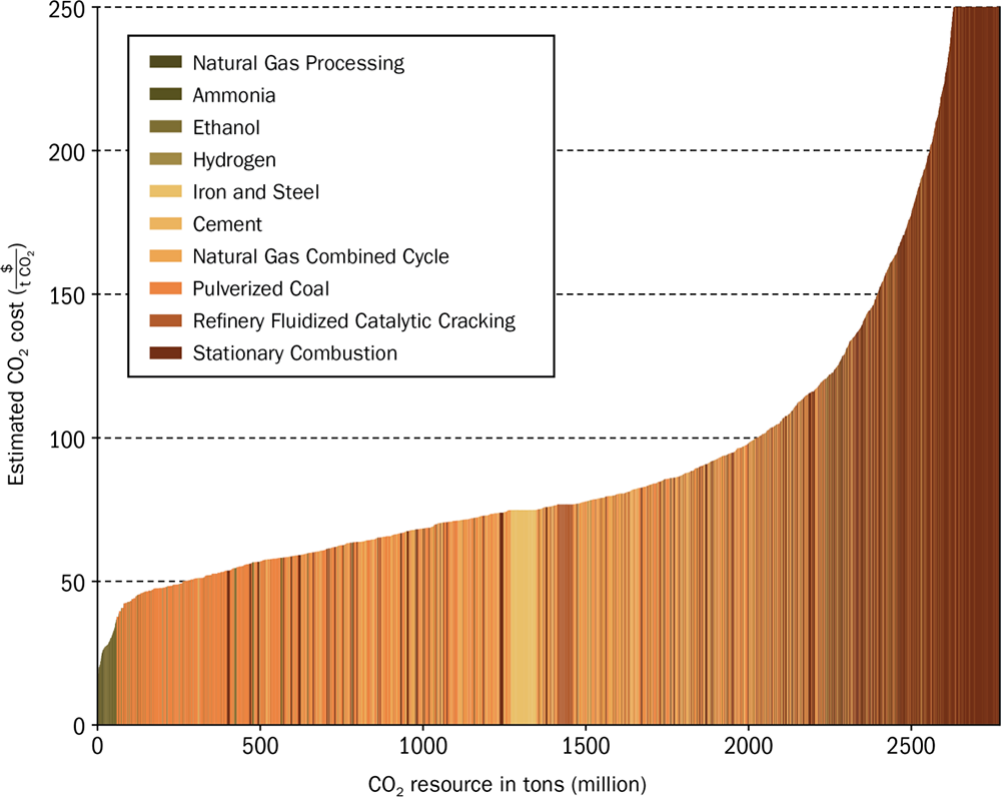
United States supply curve for point source capture of CO_2_ feedstocks. Reproduced from ref 415.

Balancing the availability, purity, and capture dynamics surrounding sourcing of CO_2_ will be key considerations for the CO_2_ conversion community. Over the near-term, first-of-a-kind CO_2_ conversion processes are naturally most likely to favor point sources exhibiting the lowest capture costs, especially those also providing low carbon intensity biogenic CO_2_ such as fermentation plants. However, as shown in Figure 26, the availability of these high purity sources is limited, representing only a minor fraction of the total CO_2_ supply. As CO_2_ conversion processes mature and begin to scale, they are likely to be forced to leverage progressively more dilute sources of CO_2_ at higher expense which could impact economic viability.

### 6.4 Economics and Scale Up of CO_2_ Conversion

Over the past decade interest in CO_2_ conversion has surged, not only in the academic world (see: Figure 1) but also commercially where follow-through from the laboratory to the private sector has seen the deployment of pilot and small commercial scale projects. A *noncomprehensive* list of recent major commissioned, announced, and canceled CO_2_ conversion projects is provided in Table 6.

**Table 6:** Prominent Commissioned, Announced, and Canceled Projects from 2020–2025

Commissioned	Announced	Canceled
**Provider**: HIF Global	**Provider**: Twelve	**Provider**: Orsted
**CO2 Source**: Biogenic from fermentation	**CO2 Source**: Biogenic CO_2_	**Project**: FlagshipONE
**Pathway**: Thermocatalysis	**Pathway**: Electrolysis	**Pathway**: Thermocatalysis
**Product**: e-Methanol/Gasoline	**Product**: Carbon Monoxide/e-SAF	**Product**: e-Methanol
**Capacity**: 130k L/yr	**Capacity**: >40,000 gal/yr	**Capacity**: 55k tonne/yr
**Location**: Punta Arenas, Chile	**Location**: Washington, USA	**Location**: Ornskodsvik, Sweden
**Year**: 2022	**Target Year**: 2025	**Year**: 2024
**ref**:^416^	**ref**:^378^	**Reason**: slower than expected demand for long-term offtakers
		**ref**:^417^
**Provider**: CRI	**Provider**: CRI	**Provider**: Haldor Topsoe
**CO2 Source**: Coke production	**CO2 Source**: Biomass combustion	**Project**: e-CO
**Pathway**: Thermocatalysis	**Pathway**: Thermocatalysis	**Pathway**: SOEC
**Product**: e-Methanol	**Product**: e-Methanol	**Product**: e-CO
**Capacity**: 110k tonnes/yr	**Capacity**: 170k tonnes/yr	**Capacity**: 96 Nm^3^ CO/hr
**Location**: Anyang, China	**Location**: Liaoyuan, China	**Location**: Ohio, USA
**Year**: 2022	**Year**: 2025	**Year**: 2021
**ref**:^387^	**ref**:^418^	**Reason**: Undisclosed
**Provider**: CRI	**Provider**: Liquid Wind	
**CO2 Source**: Waste gases from EO production	**CO2 Source**: Biogenic CO_2_	
**Pathway**: Thermocatalysis	**Pathway**: Thermocatalysis	
**Product**: e-Methanol	**Product**: e-Methanol	
**Capacity**: 100k tonnes/yr	**Capacity**: 130k tonnes/yr	
**Location**: Lianyuangang, China	**Location**: Sundsvall, Sweden	
**Year**: 2023	**Year**: 2027	
**ref**:^419^	**ref**:^420^	
**Provider**: European Energy	**Provider**: Liquid Wind	
**CO2 Source**: Biogas	**CO2 Source**: Biogenic CO_2_	
**Pathway**: Thermocatalysis	**Pathway**: Thermocatalysis	
**Product**: e-Methanol	**Product**: e-Methanol	
**Capacity**: 42k tonnes/yr	**Capacity**: 130k tonnes/yr	
**Location**: Kasso, Denmark	**Location**: Umea, Sweden	
**Year**: 2025	**Year**: 2028	
**ref**:^421^	**ref**:^422^	
**Provider**: Total/Sunfire	**Provider**: Avantium	
**CO2 Source**: Refinery CO_2_	**CO2 Source**: -	
**Pathway**: Thermocatalysis	**Pathway**: Electrocatalysis	
**Product**: e-Methanol	**Product**: PLGA Polymer	
**Capacity**: Demo	**Capacity**: 10 tonnes/yr	
**Location**: Leuna, Germany	**Location**:	
**Year**: 2021	**Year**: 2026+	
**ref**:^423^	**ref**:^424^	
**Provider**: Electrochaea	**Provider**: Reolum	
**CO2 Source**: Biogas	**CO2 Source**: Biogenic from Biomass	
**Pathway**: Biomethanation	**Pathway**: Thermocatalysis	
**Product**: Methane	**Product**: e-Methanol	
**Capacity**: 2.8m Nm^3^ Methane/yr	**Capacity**: 140 kt/yr	
**Location**: Roslev, Denmark	**Location**: Castilla y Leon, Spain	
**Year**: 2024	**Year**: 2027	
**ref**:^386^	**ref**:^425^	
**Provider**: Infinium	**Provider**: HIF Global	
**CO2 Source**: Refinery CO_2_	**CO2 Source**: Bioethanol CO_2_	
**Pathway**: Fischer–Tropsch derivative	**Pathway**: Thermocatalysis	
Product: e-Fuels	**Product**: e-Methanol	
Capacity: 2,220 gal/day	**Capacity**: 700 kt/yr	
**Location**: Texas, USA	**Location**: Paysandu, Uruguay	
**Year**: 2023	**Year**: 2025+	
**ref**:^426^,^427^	**ref**:^428^	
**Provider**: Synhelion		
**CO2 Source**: Biogas		
**Pathway**: Thermal Redox + FT		
**Product**: e-Fuels		
**Capacity**: 1,000s of liters/yr		
**Location**: Julich, Germany		
**Year**: 2024		
**ref**:^429^		

Acknowledging that this is a nonexhaustive list, the projects shown in Table 6 nevertheless reveal several trends in the scale up and deployment of CO_2_ conversion. The listed projects highlight that although there is a notable concentration in thermocatalysis, there is currently no single “winning” strategy. Rather, investments into CO_2_ conversion are being made across a breadth of technologies including electrochemistry, thermocatalysis, biological upgrading, and other hybrid approaches. The near-term targeted products, however, do show initial consolidation specifically around e-methanol and e-fuels (e.g., methanol to gasoline, RWGS to CO followed by Fischer–Tropsch). This near-term focus may be attributed to several factors. First, from a technical standpoint, the synthesis of methanol and/or FT-based fuels from C_1_ (typically CO via RWGS) + H_2_ has a rich history and has been largely derisked. Numerous providers (see: Johnson Matthey eMERALD, Topsoe G2L eFuels), now offer off-the-shelf licensable packages for CO_2_ conversion, providing immediate solutions for first of a kind plants with limited downside technical risk.^388,430^ Further, within the past five years there has been a strong market pull for decarbonization specifically in the marine and aviation sectors which are viewed as “difficult to decarbonize” through electrification alone and expected to remain reliant on liquid energy dense fuels far into the future.^15^ CO_2_-conversion pathways to e-fuels are considered to be well situated to deliver such products at a significantly reduced carbon intensity versus conventional options. Further, e-methanol doubles both as a potential fuel but also as a major platform molecule across the chemicals industry, notably as a precursor to the high-volume olefins markets.

Yet, despite the encouraging developments toward commercialization over the past several years, CO_2_ conversion technologies are expected to face long-term headwinds around breaking into the established chemicals and fuels markets. Conventional markets are dominated by economy-of-scale, large volume industries that have been optimized over multiple decades, presenting high barriers to entry with high initial capital costs and low profit margins.^431^ Consequently, meaningful scale up of CO_2_ conversion has moved at a more modest pace and some of the largest proposed capital projects have recently been terminated, citing a challenging economic environment, uncertainty in policy, and slowing demand.^417^ Encouragingly, recent techno-economic analysis (TEA) studies on CO_2_ conversion find plentiful opportunities to continue to improve on cost and overall economic competitiveness into the future. In Figure 27 and Figure 28 we show published TEA results over the past 5 years (2020–2024) specifically for producing CO and MeOH across the notable conversion pathways. It should be stressed that calculated product costs across TEA studies can vary widely in that each study may utilize different methodologies or assumptions in determining technical performance, input costs, incentives, future scenarios, etc. Our goal herein is to broadly segregate studies by product, conversion technology, and whether or not the assumptions were reflective of the current “state of technology” (SOT) or more optimiztic “future” (FUT) leaning. This approach is intended to provide an order of magnitude estimate for cost projections as well as insights into general trends.

**Figure 27:**
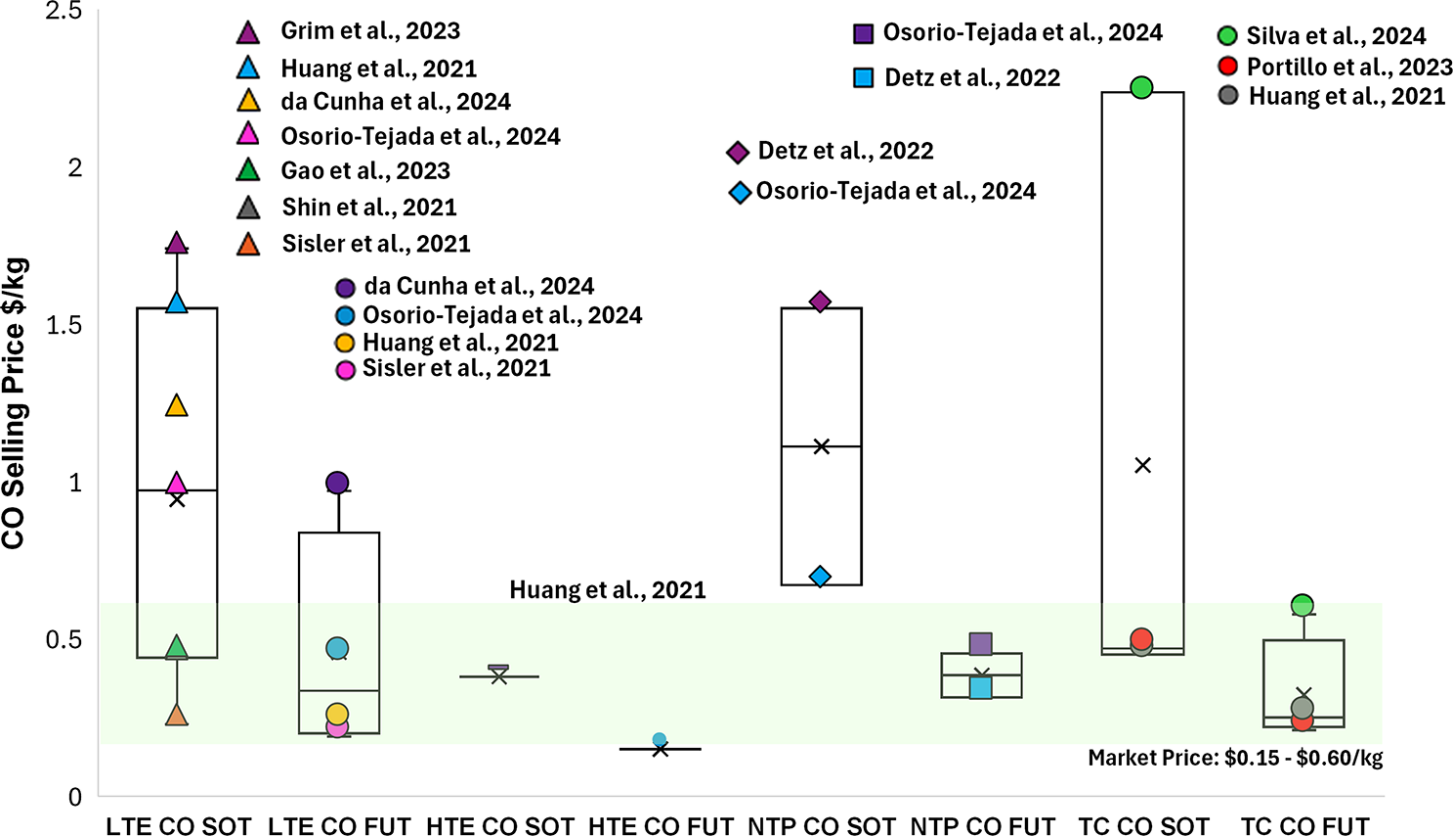
TEA studies on the conversion of CO_2_ to CO (2020–2024). LTE SOT refs:^18^,^24^,^25^,^433–436^ . LTE Fut refs:^18^,^433^,^435^,^436^ . HTE ref:^18^. NTP refs:^435^,^437^ . TC refs:^18^,^438^,^439^.

**Figure 28:**
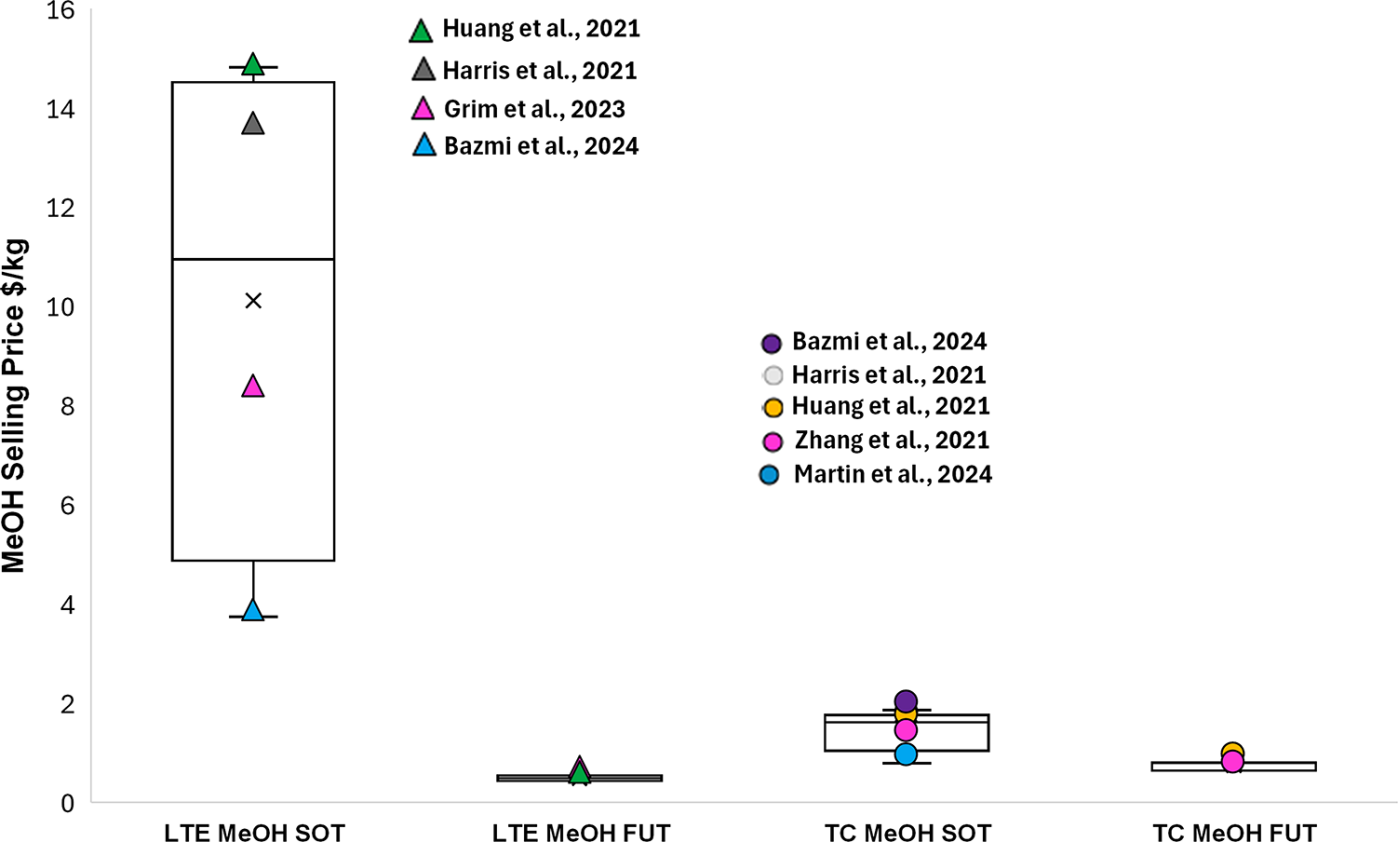
TEA studies on the conversion of CO_2_ to MeOH (2020–2024). LTE refs:^18^,^24^,^440^,^441^ . TC refs:^18^,^346^,^440–442^

In Figure 27, a review of TEA studies on CO production costs points toward a wide range of calculated production costs from ∼ $0.23/kg CO to > $2.00/kg CO. Direct comparison to incumbent CO market cost is muddied both by its different markets (e.g., as bulk synthesis gas constituent versus high purity bottled CO) at different price points, as well as sensitivity to underlying natural gas costs. Nevertheless, Figure 27 reveals that across the various pathways, all studies find that under current SOT conditions, CO_2_-based CO is not yet competitive with the lower end target US market price (c.a., 2019$0.15/kg) representative of a bulk, “over the fence” syngas cost.^432^ However, when considering the top end price range (i.e., $0.60/kg) more closely associated with high-purity gas markets combined with future case technical performance where studies account for elements such as: reducing capital costs of the core conversion technologies (e.g., electrolyzers, plasma reactors, etc.), increasing catalytic performance (e.g., CO_2_ conversion, selectivity, yield, energy efficiency), increasing productivity (e.g., current density, turnover frequency), several studies show the possibility for an economically competitive process.^7^ The studies for CO_2_-based MeOH in Figure 28 highlight a similar trend where the current state of the art exceeds the current market price, in some cases significantly so, such as for the low-temperature electrolysis pathway. These TEA results also support the observations provided in Table 6 highlighting thermocatalysis as a more near-term preferred pathway for MeOH synthesis of the two prominent options. However, unlike the CO data (a two electron process), the future looking data for MeOH (a six electron process) suggest that none of the studies found a pathway to entirely close the gap with the current market price of 2019$0.26/kg, pointing toward a potentially challenging economic perspective.

When all possible vectors for reducing e-product costs are ranked, lowering the input feedstock cost is most consistently cited the number one barrier to CO_2_ conversion economic viability, specifically in the form of sourcing low-cost electricity (or other energy feedstocks).^390,436,443,444^ With energy pricing factoring so significantly into forecasts for CO_2_ economic viability, an often-discussed cost minimization strategy is dynamic operation and load following of the electric grid and renewables generation. This approach relies on cycling key process equipment (e.g., electrolyzers, plasma reactors, etc.) on/off, letting operators take advantage of dislocations in daily electricity pricing and periods of curtailment or overgeneration either to drive the CO_2_ conversion chemistry and/or to produce H_2_ via electrolysis.^432^ While dynamic operation could be a key differentiator between conventional and power-to-X pathways, and the ability to operate selectively during times with the lowest cost of energy may offer opportunities to potentially increase economic competitiveness, the tradeoff with durability, process capacity factor, and capital utilization are critical considerations that should not be neglected.

By intentionally operating a process at low capacity factor, for example, cycling on and off on a daily basis to load follow renewables generation, in effect that plant is producing less goods for the same capital investment and correspondingly the relative impact of the CAPEX, and often product levelized cost, goes up. This effect is illustrated in Figure 29 for e-CO where C. da Cunha and Resasco show that “even when using cheap wholesale solar electricity, high capacity factors are required to make [CO_2_ conversion] economical”.^436^ They further suggest that at current electrolyzer capital costs, analysts should consider neglecting wind or solar utilization altogether, finding that the low capacity factors of direct use (without storage) make economical operation “impossible” even at discounted electricity pricing.^436^ These findings, combined with concerns around the durability impacts of cycling and the associated stresses to the equipment, highlight the critical need for lowering total system capital costs and improving overall durability, both to enable dynamic operation and improving the economic competitiveness of CO_2_ conversion.^18,445^

**Figure 29:**
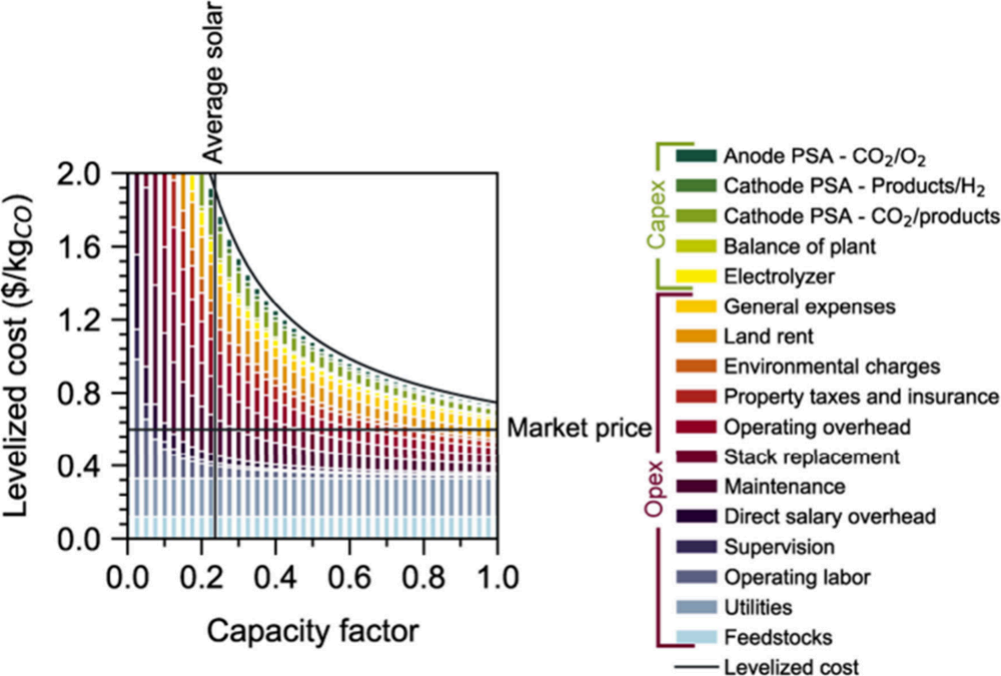
Impact of capacity factor and electricity price on the levelized cost of e-CO. Reproduced from ref 436. Copyright 2024 American Chemical Society.

Clearly, how a CO_2_ conversion pathway is deployed and integrated into the broader energy and chemicals sectors has implications for the importance and relevance of various R&D metrics. Some emerging system designs incorporate a dynamic process step, such as dynamic supply of electricity or production of hydrogen via water electrolysis. Whether downstream steps also operate dynamically or whether storage is used as a buffer has implications for key economic and therefore R&D priorities for the process as a whole. Adding buffer storage either in the form of physical hydrogen storage or electrical battery energy storage increases overall capital costs. In dynamic systems, the total capital cost determines the optimal operating capacity factor, or capital utilization. A lower overall capital cost allows the system to operate at a lower capacity factor, increasing flexibility.^406^ For this hypothetical system R&D that reduces capital costs in other components and durability during dynamic operation would be critical priorities. For a system operated at steady state, overall efficiency may be more relevant than lowering overall capital costs. In other words, the integration and operation strategies for CO_2_ conversion pathways determine optimal capital and operating cost tradeoffs and have significant implications for R&D priorities.

Ultimately, the most successful first-of-a-kind CO_2_ conversion projects to date have generally taken advantage of some combination of policy support, niche markets where consumers are willing to pay a “green premium”, and/or financial certainty through dedicated offtake agreements. Many of the first movers listed in Table 6 have also further derisked these initial investments through utilization of existing brownfield infrastructure thereby minimizing costly upfront capital investments. Other new projects have sought colocation and integration with existing low-cost waste resources such as refining off gases or waste biogas that already contain mixtures of CO/CO_2_/H_2_. Utilizing these waste resources directly as opposed to deliberate on-purpose production of the feedstocks (e.g., H_2_O electrolysis + direct air capture) minimizes feedstock cost and further derisks the investment. Moving forward and over the longer term as companies pursue larger greenfield deployments, the importance of research and development to advance the performance and cost of CO_2_ conversion at scale will become even more critical to demonstrate economic viability for large capital projects with multidecade planned lifetimes.

### 6.5 Environmental Impacts of CO_2_ Conversion at Scale

Life-cycle and other environmental assessments of CO_2_ conversion pathways typically converge on a central theme: independent of the selected pathway and core conversion technology, CO_2_-based products can be produced with a significantly lower carbon intensity than conventional routes, but typically only when powered by energy sources comprising a near zero carbon footprint.^444,446–449^ Acknowledging that CO_2_ conversion is inherently energy intensive (Table 3) and renewable electricity availability is expected to be a hotly contested resource least the next several decades (cf., Figure 23 and Figure 24), two key questions currently being asked by researchers are: (1) can we provide the amount of low-cost, low-carbon electricity needed to drive CO_2_ conversion at large scales, and, if yes, (2) should we use it for the chemicals and fuels industries over other competing use cases?^401^

Life-cycle analyses by Katelhon et al. tackle these critical questions as shown in Figure 30. They report that from an CO_2_e emissions minimization perspective, utilizing renewable electricity resources for heat pumps, electrification of light and medium duty vehicles, and use for direct electric heating (e-boilers) all show a higher climate mitigation potential than producing CO_2_-based e-products, even under the assumption of electricity emissions factor of 0 g CO_2_e/kWh.^401^ In other words, although e-products may offer substantial climate benefits relative to conventional pathways to chemicals and fuels, the calculated net CO_2_e benefit would be lower than in utilizing renewables across other use cases. Thus, from the perspective of maximizing climate benefits, they argue e-products should only be pursued once the other aforementioned options are exhausted. Katelhon et al. also note that these findings apply to grid-connected applications and that for off-grid or “behind the meter” applications of CO_2_ conversion, the results may be different. Specifically, in locations where renewable electricity resources are favorable but grid infrastructure or demand is not nearby, CO_2_ conversion represents an attractive option to utilize these stranded resources and potentially transform regionally bound electricity into more easily transportable products (e.g., energy dense liquids).^401^

**Figure 30:**
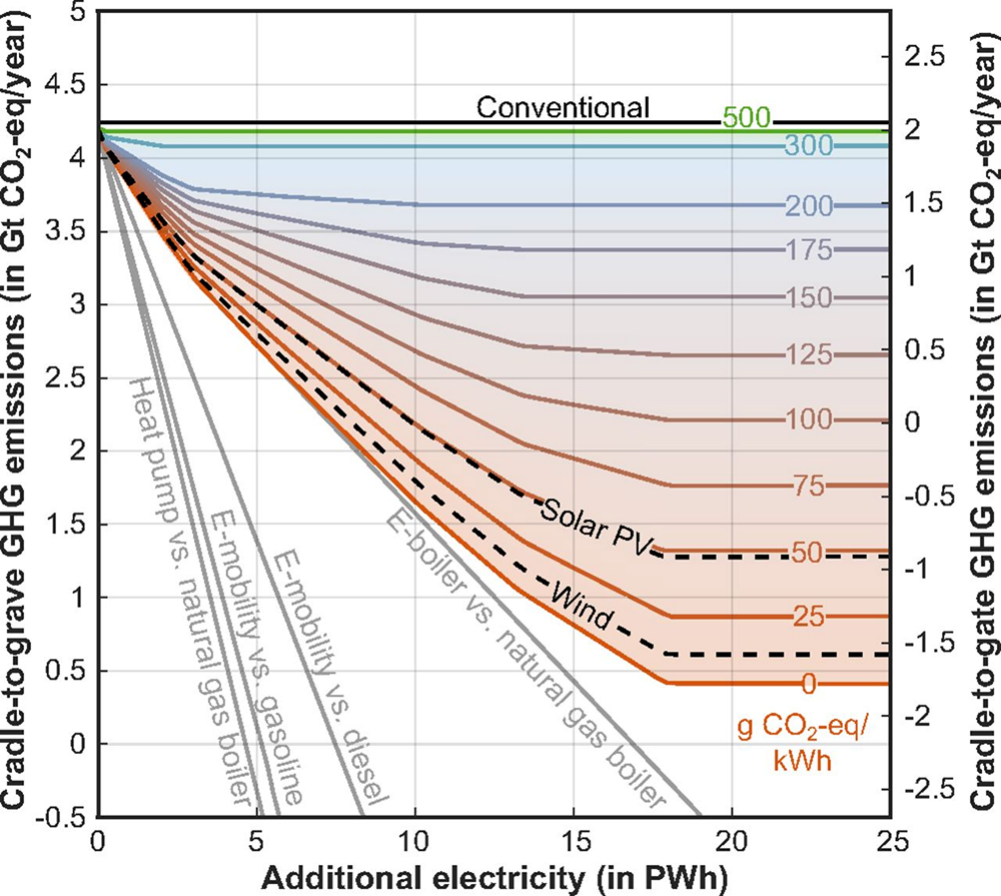
Cradle-to-grave and cradle-to-gate CO_2_e emissions for the chemical industry producing 20 large volume chemicals in 2030 as a function of the amount of additional electricity available and its carbon footprint in grams of CO_2_ equivalent/kilowatt hour. The black solid line represents CO_2_e emissions in the conventional electricity scenario with the colored lines representing various low­(er) carbon electricity scenarios. The light gray lines represent CO_2_e emissions reductions using electricity for e-mobility to substitute gasoline or diesel cars, or for heat generation in a heat pump or an e-boiler to substitute heat from natural gas boilers. Reproduced with permission from ref 401. Copyright 2019, National Academy of Sciences.

A second analysis by de Kleijne et al. in 2022 further supports these conclusions. Their life-cycle analysis converges around the idea that from a climate perspective, making “e-products”—in their case electrolytic H_2_ instead of CO_2_ conversion to products—to have a lower overall benefit than similar commonly cited use cases. Specifically, shown in Figure 31, de Kleijne concludes that utilizing renewable electricity to produce “green” H_2_ avoids fewer CO_2_e emissions than utilizing electricity for transportation, heat pumps, or simply displacing conventional power generation (e.g., coal, natural gas).^450^ This finding and concept of environmental opportunity cost was further corroborated by Ravikumar et al. who studied CO_2_-based methanol production finding, that until the carbon intensity of the electricity grid drops below 82 g CO_2_e/kWh, is it more environmentally beneficial to supply renewable energy to the grid rather that use it to convert CO_2_ into methanol.^451^

**Figure 31:**
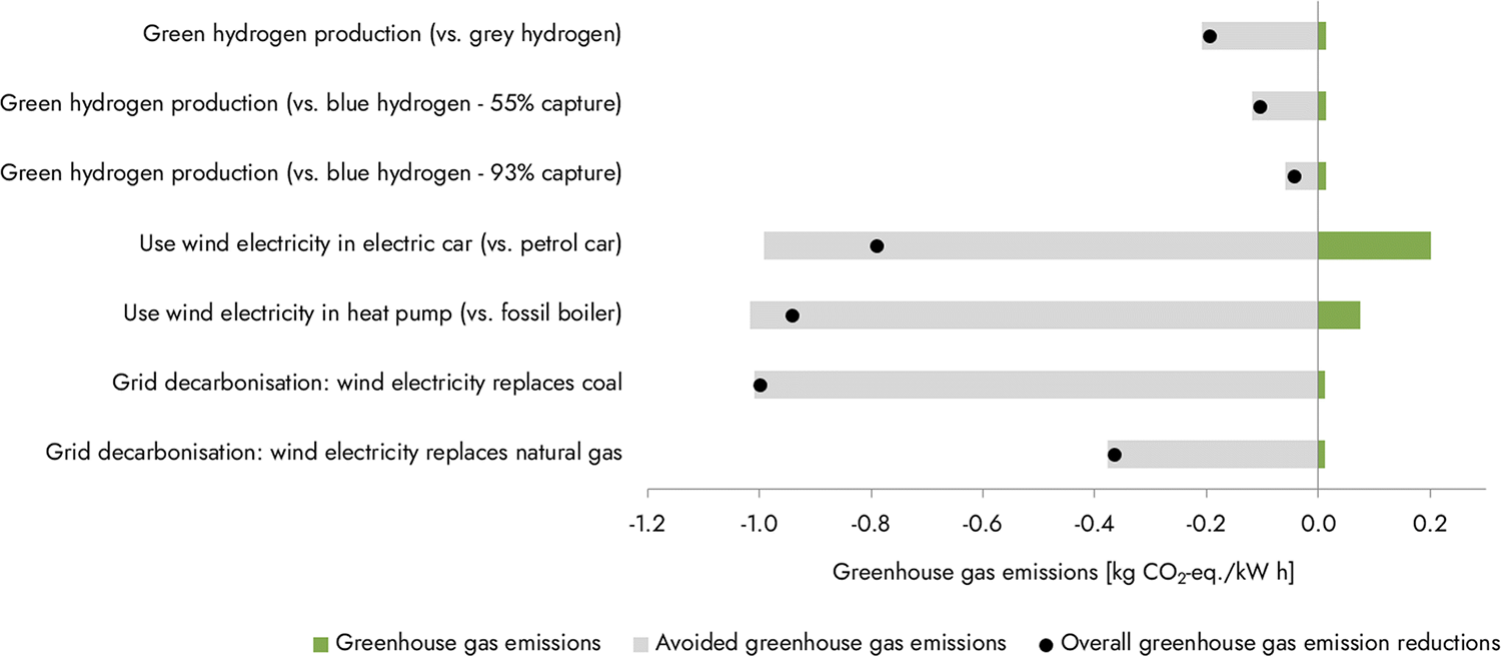
CO_2_ emissions and avoided emissions for different ways of using 1 kWh of offshore wind generation. Reproduced with permission from ref 450. Copyright 2022, Royal Society of Chemistry.

Indeed, from governmental mandates to tax incentives to offering consumers environmentally friendly alternatives in the marketplace, there are many potential reasons why companies have and will continue to pursue CO_2_ conversion. However, these analyses show that owing to their intrinsic energy intensive properties, CO_2_-based e-products carry a significant energy “opportunity cost” and that at least over the near-term, may not always represent the best way to maximize climate benefits, depending on how other industries evolve and decarbonize over time. Nevertheless, over the long-term, the massive quantities of available CO_2_ combined with the insatiable demand for chemicals and fuels points to a future where CO_2_-based products can play a significant role in fostering a circular economy. Consequently, research and development efforts supporting CO_2_ conversion should continue such that once economic, policy, and environmental conditions are favorable for these technologies to take significant market share, the community is ready.

## 7 Conclusions and Outlook

Born out of a desire to stem rising levels of atmospheric CO_2_, the field of CO_2_ conversion and utilization has experienced a period of rapid growth over the past two decades. Offering the ability to repurpose “waste” carbon resources into chemicals and fuels by way of low-carbon electricity, CO_2_ conversion is heralded as a means to bring the benefits of deep electrification to many of the otherwise “difficult to decarbonize” industrial sectors. Further, with the ability to tap into the approximately 3,330 gigatonnes of CO_2_ (i.e., ∼ 900 gigatonnes of carbon) in the atmosphere from any point in the world via direct air capture, CO_2_ conversion technologies can be decoupled from existing infrastructure and other geographical constraints, thereby improving supply chain resiliency, promoting carbon circularity, and offering new opportunities for the chemical storage of stranded energy assets.

In this manuscript we have reviewed the most recent advancements (2020–2025) across prominent pathways for CO_2_ conversion spanning the fields of electrochemistry, photochemistry, biochemistry, plasma, and thermocatalysis. Specifically, we have highlighted how the collective fundamental understanding of chemical mechanisms and challenges for electron-mediated and H_2_-mediated CO_2_ conversion have evolved over the past six years and how these new insights have contributed toward improved technical metrics and scale up of CO_2_ conversion. This review also discussed the outlook on the availability of critical feedstocks for CO_2_ conversion including electricity, H_2_, and CO_2_ itself, finding the availability of low-cost and low-carbon electricity to be the principle limiting factor in widespread adoption of CO_2_ conversion for likely the next several decades. We further discussed the current commercial and economic status of CO_2_ conversion, finding recent commercial activity to be largely dominated by e-methanol and e-fuel production efforts. TEA studies performed during this time predict the cost of these, and other CO_2_-based products, to exceed that of conventional methods over the nearest-term, with some studies finding that if sharp reductions in CAPEX, feedstock costs, and energy use can be realized over time there may be pathways to reaching price points at or below those of conventional pathways used today. Finally, we conclude the review with a discussion on the environmental outlook for CO_2_ conversion, highlighting that the critical ingredient for ultimately lowering emissions will be the carbon footprint of the energy source (e.g., electricity or H_2_). We also highlight the concept of “opportunity cost” recently discussed across life-cycle analyses, noting that due to the significant energy cost associated with CO_2_ conversion, the community must weigh the benefits of diverting low-carbon electricity toward CO_2_ conversion versus the myriads of other potential use cases and the relative benefits of each.

## Supplementary Material



## Notes

Half-cell reactions for CO_2_RR; Anatomy of a CO_2_ conversion electrolyzer; metrics used in CO_2_RR; advantages, challenges, and failure modes of LTE CO_2_RR electrolyzers; comparative review of the chemical advantages, challenges, and reported performance of dominant low-temperature CO_2_ electrolyzer architectures from the 2020–2025 literature; and review of key chemical degradation mechanisms in low-temperature CO_2_ electrolyzers, their performance impacts, and mitigation strategies (PDF)
