# Supplementary material for: The Chemistry of CO2 Conversion: A Review
Source: Chem Rev. 2026 Apr 21;126(9):5028–82. doi: 10.1021/acs.chemrev.5c00361 (PMC13178081; doi:10.1021/acs.chemrev.5c00361)
Supplement: Supplementary file 1 [file cr5c00361_si_001.pdf]

Supporting Information for The Chemistry of CO_2 Conversion: A Review

R. Gary Grim[1]*, Alex Badgett[1], Wade A. Braunecker[1], Michael T. Guarnieri[1], Susan E. Habas[1], Christopher Hahn[2], Kenneth Neyerlin[1], Aditya Prajapati[2], Daniel A. Ruddy[1], Roxanne Walker[1]

1: National Laboratory of the Rockies, 15013 Denver W Pkwy, Golden, CO 80401
2: Lawrence Livermore National Laboratory, 7000 East Ave, Livermore, CA 94550
*Corresponding Author: gary.grim@nlr.gov

1. Half-cell reactions for CO2RR

Table 1: Key half reactions for CO2RR studied in the literature including the parasitic hydrogen evolution reaction (HER) on the cathode and oxygen evolution reaction (OER) on the anode

Sr. no.	Product	Reaction	E_0 (V vs. RHE)
1	CO	$CO_2 + H_2O + 2e^- \rightarrow CO_{(g)} + 2OH^-$	-0.10
2	HCOOH	$CO_2 + 2H_2O - +2e^- \rightarrow HCOOH_{(aq)} + 2OH^-$	-0.12
3	CH_3OH	$CO_2 + 5H_2O + 6e^- \rightarrow CH_3OH_{(aq)} + H_2O + 6OH^-$	0.03
4	CH_4	$CO_2 + 6H_2O + 8e^- \rightarrow CH_{4(g)} + 8OH^-$	0.17
5	CH_3COOH	$2CO_2 + 6H_2O + 8e^- \rightarrow CH_3COOH_{(aq)} + 8OH^-$	0.11
6	C_2H_4	$2CO_2 + 8H_2O + 12e^- \rightarrow C_2H_{4(g)} + 12OH^-$	0.08
7	C_2H_5OH	$2CO_2 + 9H_2O + 12e^- \rightarrow C_2H_5OH_{(aq)} + 12OH^-$	0.09
8	H_2	$2H_2O + 2e^- \rightarrow H_{2(g)} + 2OH^-$	0
9	O_2	$4OH^- \rightarrow O_{2(g)} + 2H_2O + 4e^-$	1.23

2. Anatomy of a CO_2 Conversion Electrolyzer

A typical LTE-MEA is a composite of several key components, each with a specific chemical function as described below:

- **Cathode:** The cathode is the negative electrode and the site of the CO_2 reduction reaction (CO_2RR). It is typically fabricated as a gas diffusion electrode (GDE), a porous structure designed to facilitate the meeting of the three necessary phases: gaseous CO_2 reactant, a liquid electrolyte or water phase for ion transport and proton donation, and the solid electrocatalyst surface. [1] The GDE consists of a catalyst layer containing catalytically active nanoparticles (e.g., silver, copper, tin etc.) supported on a porous, electrically conductive gas diffusion layer (GDL), which is often made of carbon-based substrate (paper or cloth) or PTFE.[2,3] The precise engineering of this three-phase interface is critical for achieving high reaction rates.
- **Anode:** The anode is the positive electrode where an oxidation reaction occurs to complete the electrical circuit. In most LTE systems, this is OER, where water is oxidized to produce oxygen, protons, and electrons (Table 1),. The anode environment is highly oxidative, necessitating the use of corrosion-resistant materials. The catalyst is typically a precious metal oxide, such as iridium oxide (IrO_2) supported on a robust porous transport layer (PTL) made of titanium.[4] The choice of anode materials is a critical factor in the overall durability and cost of the electrolyzer system.

- **Membrane:** Positioned between the cathode and anode is an ion-conducting polymer membrane. Its primary role is to physically separate the two electrode compartments, preventing the mixing of gaseous CO_2 and O_2 while selectively transporting ions to maintain charge balance in the cell. The most common type of membrane used in LTE is the anion exchange membrane (AEM), which is designed to selectively transport hydroxide ions (OH^-) or other anions.[5]
- **Flow Fields:** The MEA is compressed between two conductive plates that have flow-field channels machined into their surfaces. These channels are responsible for uniformly distributing the reactants (gaseous CO_2 to the cathode, liquid anolyte to the anode) across the electrode surfaces and for efficiently removing the product streams.[6]

3. Metrics used in CO_2RR

To evaluate, compare, and advance LTE systems, the research community relies on a set of key performance indicators (KPIs). These metrics provide a quantitative framework for assessing the efficiency, productivity, and overall viability of a given electrolyzer technology.

- Faradaic Efficiency (FE): Expressed as a percentage, the Faradaic efficiency quantifies the selectivity of the electrochemical process. It is defined as the fraction of the total electrical charge (i.e., the number of electrons) passed through the cell that is used to form a specific desired product. A high FE (e.g., >90%) is critical because it minimizes the formation of unwanted byproducts, which simplifies the costly downstream separation and purification processes and maximizes the value derived from the electrical input.5 It is calculated as:

$$FE_{product} = \frac{(n_{product} \times z \times F)}{Q_{total}}$$

  where $n_{product}$ is the number of moles of the product formed, z is the number of electrons transferred per mole of product, F is the Faraday constant, and Q_{total} is the total charge passed.

- **Partial Current Density (j):** Measured in mA/cm^2, the current density represents the rate of the electrochemical reaction per unit of geometric electrode area. The partial current density for a specific product is the total current density multiplied by its FE. High current densities (e.g., >200–500 mA/cm^2 are essential for economic viability as they correspond to high production rates, which reduces the required electrolyzer stack size and, consequently, the capital expenditure (CAPEX) for a given production capacity.[7]

- **Full-Cell Energy Efficiency (EE):** This KPI, also expressed as a percentage, is a measure of the overall energy conversion efficiency of the process. It is the ratio of the standard Gibbs free energy of formation stored in the chemical products to the total electrical energy consumed by the electrolyzer cell. Since electricity is a primary driver of operational expenditure (OPEX), a high EE is paramount for the economic competitiveness of LTE technology. A threshold of approximately 50% EE is often cited as a critical target for products like ethylene and CO to compete with conventional fossil-fuel-based production routes. [1] The EE is calculated as:

$$EE = \frac{E^0 \times FE}{V_{cell}}$$

  where E^0 is the thermodynamic equilibrium potential for the reaction, FE is the Faradaic efficiency for the desired product, and V_{cell} is the actual operating full-cell voltage.

- **Single-Pass Carbon Conversion (SPC):** SPC is the fraction of the CO_2 feedstock fed to the cathode that is converted into chemical products in a single pass through the reactor. This metric is a direct measure of feedstock utilization efficiency. A low SPC implies that a large amount of unreacted CO_2 must be separated from the product stream and recycled back to the electrolyzer inlet. This separation and recycling loop adds significant capital cost, complexity, and a substantial energy penalty to the overall system, undermining its economic feasibility. Industrially relevant benchmarks for SPC are on the order of 30% for C_1 products and 15% for C_2 products.[7]

4. Advantages, Challenges, and Failure modes of LTE CO_2RR electrolyzers

Table 2: Comparative review of the chemical advantages, challenges, and reported performance of dominant low-temperature CO_2 electrolyzer architectures from the 2020–2025 literature.

Architecture	Key Chemical Advantage	Primary Chemical Challenge	Representative Performance (2020-2025 Literature)
**Anion Exchange Membrane (AEM)**	Suppresses HER via local alkalinity, enabling use of PGM-free anode catalysts.	Extensive carbonate formation and crossover. Stoichiometric CO_2 loss, typically ~50% SPC for CO). Salt precipitation and hydroxide-based membrane degradation.	For CO production, FEs >90% at j > 200 mA/cm^2 reported; Typical SPC and EE <50% [8]
**Reverse-Bias Bipolar Membrane (BPM)**	Near-complete suppression of carbonate crossover via junction protonation.	Acidic cathode environment drives strong HER competition, lowering CO_2RR FE; additional voltage (~0.2–0.5 V) required for water dissociation.	SPC values >70% for C_{2+} products demonstrated, though often with reduced FE and higher cell voltages (≈4–5 V) relative to AEM systems.[9] Recent studies use alkali-cation modulation to improve selectivity and suppress HER
**Forward-Bias Bipolar Membrane (BPM)**	Maintains alkaline cathode (suppresses HER); Enables pure water feed, preventing crossover and salt precipitation.	Accumulation of H_2O, CO_2 at the AEL/CEL junction can cause mechanical delamination and catastrophic failure.	CO FE >80% at CO partial current densities >200 mA cm^{-2} and cell voltages <3.0 V in pure-water-fed forward-bias BPM MEAs.[10] Long-term stability remains limited by gas-induced junction damage and delamination. Recent advances in engineering porous junctions have significantly improved durability.
**Proton Exchange Membrane (PEM)**	Complete elimination of carbonate formation, leading to high carbon utilization and simplified system design.	Massive kinetic competition from the HER in the bulk acidic environment. Requires PGM anode catalysts.	Achieved >93% FE for formic acid at 600 mA cm^{-2}, with ≈91% single-pass CO2 conversion and continuous operation for >5,200 h at 2.2 V, by pairing CO_2 reduction with hydrogen oxidation in a PEM cell.[11]

			Acidic “cation-effect” studies have separately shown that alkali cations can suppress HER and promote CO_2RR in strong acid.[12]

Table 3: A review of key chemical degradation mechanisms in low-temperature CO_2 electrolyzers, their performance impacts, and mitigation strategies.

Degradation Mode	Underlying Chemical Mechanism	Affected Component(s)	Impact on KPIs	Mitigation Strategy
**Salt Precipitation**	Cation crossover from anolyte meets locally generated (bi)carbonate at the cathode, exceeding the local solubility limit.	Cathode GDL, Catalyst Layer	Increased cell voltage (ohmic & mass transport loss), decreased FE for CO_2RR, catastrophic failure.	Use cations with higher salt solubility (e.g., Cs^+); operate at elevated temperature (>50 ^oC); design membranes to reduce cation transport.
**GDL/Catalyst Layer Flooding**	Accumulation of liquid water in porous structures, blocking gas transport pathways due to an imbalance between water supply and removal.	GDL, Catalyst Layer	Severe mass transport limitation, leading to a sharp decrease in current density and FE.	Optimized GDL hydrophobicity; Precise control of CO_2 humidification and cell temperature to balance electro-osmotic drag and back-diffusion.
**Membrane Dehydration**	Loss of water from the ionomer/membrane, often due to insufficient humidification or excessive Joule heating.	Membrane, Ionomer	Increased ohmic resistance (lower EE), membrane cracking, and potential for reactant gas crossover.	Proper humidification of feed CO_2 stream; effective thermal management to prevent hotspots; Control of gas and liquid backpressure
**Anodic Carbon Corrosion**	Electrochemical oxidation of carbon to CO/CO_2 at the high oxidative potentials of the anode($C + 2H_2O \rightarrow CO_2 + 4H^+ + 4e^-$)	Anode GDL, Anode Catalyst Support	Structural collapse of the anode, loss of catalyst, increased cell voltage.	Replace carbon-based anode components with corrosion-resistant materials like Ti or Platinized Ti.
**Catalyst Reconstruction**	Atomic restructuring of the catalyst surface under high applied potential, leading to changes in active sites and particle morphology.	Cathode Catalyst	Loss of active surface area (lower current density); alteration of active sites (loss of selectivity/FE).	Catalyst anchoring strategies; operation at lower overpotentials; development of intrinsically more stable catalyst compositions.

References

(1) Lee, H.; Kwon, S.; Park, N.; Cha, S. G.; Lee, E.; Kong, T.-H.; Cha, J.; Kwon, Y. Scalable Low-Temperature CO2 Electrolysis: Current Status and Outlook. *JACS Au* **2024**, *4* (9), 3383–3399. https://doi.org/10.1021/jacsau.4c00583.

(2) Biemolt, J.; Singh, J.; Prats Vergel, G.; Pelzer, H. M.; Burdyny, T. Preventing Salt Formation in Zero-Gap CO2 Electrolyzers by Quantifying Cation Accumulation. *ACS Energy Lett.* **2025**, *10* (2), 807–814. https://doi.org/10.1021/acsenergylett.4c03242.

(3) García de Arquer, F. P.; Dinh, C.-T.; Ozden, A.; Wicks, J.; McCallum, C.; Kirmani, A. R.; Nam, D.-H.; Gabardo, C.; Seifitokaldani, A.; Wang, X.; Li, Y. C.; Li, F.; Edwards, J.; Richter, L. J.; Thorpe, S. J.; Sinton, D.; Sargent, E. H. CO2 Electrolysis to Multicarbon Products at Activities Greater than 1 A Cm−2. *Science* **2020**, *367* (6478), 661–666. https://doi.org/10.1126/science.aay4217.

(4) Vass, Á.; Kormányos, A.; Kószó, Z.; Endrődi, B.; Janáky, C. Anode Catalysts in CO2 Electrolysis: Challenges and Untapped Opportunities. *ACS Catal.* **2022**, *12* (2), 1037–1051. https://doi.org/10.1021/acscatal.1c04978.

(5) Salvatore, D. A.; Gabardo, C. M.; Reyes, A.; O'Brien, C. P.; Holdcroft, S.; Pintauro, P.; Bahar, B.; Hickner, M.; Bae, C.; Sinton, D.; Sargent, E. H.; Berlinguette, C. P. Designing Anion Exchange Membranes for CO2 Electrolysers. *Nat Energy* **2021**, *6* (4), 339–348. https://doi.org/10.1038/s41560-020-00761-x.

(6) Yuan, S.; Wang, R.; Xue, R.; Wu, L.; Zhang, G.; Li, H.; Wang, Q.; Yin, J.; Luo, L.; Shen, S.; An, L.; Yan, X.; Zhang, J. Flow Field Design Matters for High Current Density Zero-Gap CO2 Electrolyzers. *ACS Energy Lett.* **2024**, *9* (12), 5945–5954. https://doi.org/10.1021/acsenergylett.4c02534.

(7) Seger, B.; Kastlunger, G.; Bagger, A.; Scott, S. B. A Perspective on the Reaction Mechanisms of CO2 Electrolysis. *ACS Energy Lett.* **2025**, *10* (5), 2212–2227. https://doi.org/10.1021/acsenergylett.4c03599.

(8) Ramdin, M.; Moultos, O. A.; van den Broeke, L. J. P.; Gonugunta, P.; Taheri, P.; Vlugt, T. J. H. Carbonation in Low-Temperature CO2 Electrolyzers: Causes, Consequences, and Solutions. *Ind. Eng. Chem. Res.* **2023**, *62* (18), 6843–6864. https://doi.org/10.1021/acs.iecr.3c00118.

(9) Xie, K.; Miao, R. K.; Ozden, A.; Liu, S.; Chen, Z.; Dinh, C.-T.; Huang, J. E.; Xu, Q.; Gabardo, C. M.; Lee, G.; Edwards, J. P.; O'Brien, C. P.; Boettcher, S. W.; Sinton, D.; Sargent, E. H. Bipolar Membrane Electrolyzers Enable High Single-Pass CO2 Electroreduction to Multicarbon Products. *Nat Commun* **2022**, *13* (1), 3609. https://doi.org/10.1038/s41467-022-31295-3.

(10) Heßelmann, M.; Lee, J. K.; Chae, S.; Tricker, A.; Keller, R. G.; Wessling, M.; Su, J.; Kushner, D.; Weber, A. Z.; Peng, X. Pure-Water-Fed Forward-Bias Bipolar Membrane CO2 Electrolyzer. *ACS Appl. Mater. Interfaces* **2024**, *16* (19), 24649–24659. https://doi.org/10.1021/acsami.4c02799.

(11) Gu, J.; Liu, S.; Ni, W.; Ren, W.; Haussener, S.; Hu, X. Modulating Electric Field Distribution by Alkali Cations for CO2 Electroreduction in Strongly Acidic Medium. *Nat Catal* **2022**, *5* (4), 268–276. https://doi.org/10.1038/s41929-022-00761-y.

(12) Huang, J. E.; Li, F.; Ozden, A.; Sedighian Rasouli, A.; García de Arquer, F. P.; Liu, S.; Zhang, S.; Luo, M.; Wang, X.; Lum, Y.; Xu, Y.; Bertens, K.; Miao, R. K.; Dinh, C.-T.; Sinton, D.; Sargent, E. H. CO $_2$ Electrolysis to Multicarbon Products in Strong Acid. *Science* **2021**, *372* (6546), 1074–1078. https://doi.org/10.1126/science.abg6582.
